# Supplementary material for: Are the Post-COVID-19 Posttraumatic Stress Disorder (PTSD) Symptoms Justified by the Effects of COVID-19 on Brain Structure? A Systematic Review
Source: J Pers Med. 2023 Jul 15;13(7):1140. doi: 10.3390/jpm13071140 (PMC10381510; doi:10.3390/jpm13071140)
Supplement: Supplementary file 1 [file jpm-13-01140-s001.zip › jpm-2404566-supplementary.pdf]

Supplementary Table S1. Studies included vs. excluded with reasons for exclusion for each study

1	Niu J, Shen L, Huang B, Ye F, Zhao L, Wang H, Deng Y, Tan W. Non-invasive bioluminescence imaging of HCoV-OC43 infection and therapy in the central nervous system of live mice. Antiviral Res. 2020;173:104646. doi: 10.1016/j.antiviral.2019.104646. Epub 2019 Nov 6.	Animal
2	Takyu S, Ahmed AM, Yoshida E, Tashima H, Kumagai M, Yamashita T, Yamaya T. Design study of a brain-dedicated time-of-flight PET system with a hemispherical detector arrangement. Phys Med Biol. 2020;65(3):035012. doi: 10.1088/1361-6560/ab63ee.	No COVID-19
3	Andica C, Kamagata K, Hayashi T, Hagiwara A, Uchida W, Saito Y, Kamiya K, Fujita S, Akashi T, Wada A, Abe M, Kusahara H, Hori M, Aoki S. Scan-rescan and inter-vendor reproducibility of neurite orientation dispersion and density imaging metrics. Neuroradiology. 2020;62(4):483-494. doi: 10.1007/s00234-019-02350-6. Epub 2019 Dec 27.	No COVID-19
4	Olaseni AO, Akinsola OS, Agberotimi SF, Oguntayo R. Psychological distress experiences of Nigerians during Covid-19 pandemic; the gender difference. Soc Sci Humanit Open. 2020;2(1):100052. doi: 10.1016/j.ssaho.2020.100052. Epub 2020 Aug 26.	Unfocused
5	Jung H, Jung SY, Lee MH, Kim MS. Assessing the presence of post-traumatic stress and turnover intention among nurses post-Middle East Respiratory Syndrome outbreak: The importance of supervisor support. Workplace Health Saf. 2020 Jul;68(7):337-345. doi: 10.1177/2165079919897693. Epub 2020 Mar 9.	Unfocused
6	Poyiadji N, Shahin G, Noujaim D, Stone M, Patel S, Griffith B. COVID-19-associated acute hemorrhagic necrotizing encephalopathy: Imaging features. Radiology. 2020;296(2):E119-E120. doi: 10.1148/radiol.2020201187. Epub 2020 Mar 31.	Case
7	Galougahi MK, Ghorbani J, Bakhshayeshkaram M, Naeini AS, Haseli S. Olfactory bulb magnetic resonance imaging in SARS-CoV-2-induced anosmia: The first report. Acad Radiol. 2020;27(6):892-893. doi: 10.1016/j.acra.2020.04.002. Epub 2020 Apr 11.	Case
8	Chevance A, Gourion D, Hoertel N, Llorca PM, Thomas P, Bocher R, Moro MR, Laprévote V, Benyamina A, Fossati P, Masson M, Leaune E, Leboyer M, Gaillard R. Assurer les soins aux patients souffrant de troubles psychiques en France pendant l'épidémie à SARS-CoV-2 [Ensuring mental health care during the SARS-CoV-2 epidemic in France: A narrative review]. Encéphale. 2020;46(3S):S3-S13. doi: 10.1016/j.encep.2020.03.001. Epub 2020 Apr 2.	Review
9	Chevance A, Gourion D, Hoertel N, Llorca PM, Thomas P, Bocher R, Moro MR, Laprévote V, Benyamina A, Fossati P, Masson M, Leaune E, Leboyer M, Gaillard R. Ensuring mental health care during the SARS-CoV-2 epidemic in France: A narrative review. Encéphale. 2020;46(3):193-201. doi: 10.1016/j.encep.2020.04.005. Epub 2020 Apr 22.	Review
10	El-Hage W, Hingray C, Lemogne C, Yrondi A, Brunault P, Bienvenu T, Etain B, Paquet C, Gohier B, Bennabi D, Birmes P, Sauvaget A, Fakra E, Prieto N, Bulteau S, Vidailhet P, Camus V, Leboyer M, Krebs MO, Aouizerate B. Les professionnels de santé face à la pandémie de la maladie à coronavirus (COVID-19) : quels risques pour leur santé mentale ? [Health professionals facing the coronavirus disease 2019 (COVID-19) pandemic: What are the mental health risks?]. Encéphale. 2020;46(3S):S73-S80. doi: 10.1016/j.encep.2020.04.008. Epub 2020 Apr 22.	Review
11	Guo Q, Zheng Y, Shi J, Wang J, Li G, Li C, Fromson JA, Xu Y, Liu X, Xu H, Zhang T, Lu Y, Chen X, Hu H, Tang Y, Yang S, Zhou H, Wang X, Chen H, Wang Z, Yang Z. Immediate psychological distress in quarantined patients with COVID-19 and its association with peripheral inflammation: A mixed-method study. Brain Behav Immun. 2020;88:17-27. doi: 10.1016/j.bbi.2020.05.038. Epub 2020 May 19.	Unrelated
12	Ogier M, Andéol G, Sagui E, Dal Bo G. How to detect and track chronic neurologic sequelae of COVID-19? Use of auditory brainstem responses and neuroimaging for long-term patient follow-up. Brain Behav Immun Health. 2020;5:100081. doi: 10.1016/j.bbih.2020.100081. Epub 2020 May 15.	Review
13	Mahammedi A, Saba L, Vagal A, Leali M, Rossi A, Gaskill M, Sengupta S, Zhang B, Carriero A, Bachir S, Crivelli P, Paschè A, Premi E, Padovani A, Gasparotti R. Imaging of neurologic disease in hospitalized patients with COVID-19: An Italian multicenter retrospective observational study. Radiology. 2020;297(2):E270-E273. doi: 10.1148/radiol.2020201933. Epub 2020 May 21.	Unsuitable
14	Md Noh MSF. Brain imaging findings in COVID-19: What do we know so far? J Neuroradiol. 2020;47(5):329-330. doi: 10.1016/j.neurad.2020.05.004. Epub 2020 May 19.	Review
15	Jain R, Young M, Dogra S, Kennedy H, Nguyen V, Jones S, Bilaloglu S, Hochman K, Raz E, Galetta S, Horwtiz L. COVID-19 related neuroimaging findings: A signal of thromboembolic complications and a strong prognostic marker of poor patient outcome. J Neurol Sci. 2020;414:116923. doi: 10.1016/j.jns.2020.116923. Epub 2020 May 19.	Unsuitable
16	Radmanesh A, Raz E, Zan E, Derman A, Kaminetzky M. Brain imaging use and findings in COVID-19: A single academic center experience in the epicenter of disease in the United States. AJNR Am J Neuroradiol. 2020;41(7):1179-1183. doi: 10.3174/ajnr.A6610. Epub 2020 May 28.	Unsuitable
17	Politi LS, Salsano E, Grimaldi M. Magnetic Resonance Imaging Alteration of the Brain in a Patient With Coronavirus Disease 2019 (COVID-19) and Anosmia. JAMA Neurol. 2020;77(8):1028-1029. doi: 10.1001/jamaneurol.2020.2125.	Case
18	Qi R, Chen W, Liu S, Thompson PM, Zhang LJ, Xia F, Cheng F, Hong A, Surento W, Luo S, Sun ZY, Zhou CS, Li L, Jiang X, Lu GM. Psychological morbidities and fatigue in patients with confirmed COVID-19 during disease outbreak: prevalence and associated biopsychosocial risk factors. medRxiv. 2020 May 11:2020.05.08.20031666. doi: 10.1101/2020.05.08.20031666. Preprint.	Unfocused
19	Tang W, Hu T, Yang L, Xu J. The role of alexithymia in the mental health problems of home-quarantined university students during the COVID-19 pandemic in China. Pers Individ Dif. 2020;165:110131. doi: 10.1016/j.paid.2020.110131. Epub 2020 Jun 5.	Unrelated
20	Nicholson P, Alshafai L, Krings T. Neuroimaging findings in patients with COVID-19. AJNR Am J Neuroradiol. 2020;41(8):1380-1383. doi: 10.3174/ajnr.A6630. Epub 2020 Jun 11.	Case
21	Katal S, Balakrishnan S, Gholamrezanezhad A. Neuroimaging and neurologic findings in COVID-19 and other coronavirus infections: A systematic review in 116 patients. J Neuroradiol. 2021;48(1):43-50. doi: 10.1016/j.neurad.2020.06.007. Epub 2020 Jun 27.	Review
22	Hernández-Fernández F, Sandoval Valencia H, Barbella-Aponte RA, Collado-Jiménez R, Ayo-Martín Ó, Barrena C, Molina-Nuevo JD, García-García J, Lozano-Setién E, Alcahut-Rodriguez C, Martínez-Martín Á, Sánchez-López A, Segura T. Cerebrovascular disease in patients with COVID-19: neuroimaging, histological and clinical description. Brain. 2020 Oct 1;143(10):3089-3103. doi: 10.1093/brain/awaa239.	Unfocused
23	Elhai JD, Yang H, McKay D, Asmundson GJG. COVID-19 anxiety symptoms associated with problematic smartphone use severity in Chinese adults. J Affect Disord. 2020;274:576-582. doi: 10.1016/j.jad.2020.05.080. Epub 2020 May 27.	Unfocused
24	Soldatelli MD, Amaral LFD, Veiga VC, Rojas SSO, Omar S, Marussi VHR. Neurovascular and perfusion imaging findings in coronavirus disease 2019: Case report and literature review.Neuroradiol J. 2020;33(5):368-373. doi: 10.1177/1971400920941652. Epub 2020 Jul 15.	Review
25	Kremer S, Lersy F, Anheim M, Merdji H, Schenck M, Oesterlé H, Bolognini F, Messie J, Khalil A, Gaudemer A, Carré S, Alleg M, Lecocq C, Schmitt E, Anxionnat R, Zhu F, Jager L, Nesser P, Mba YT, Hmeydia G, Benzakoun J, Oppenheim C, Ferré JC, Maamar A, Carsin-Nicol B, Comby PO, Ricolfi F, Thouant P, Boutet C, Fabre X, Forestier G, de Beaurepaire I, Bornet G, Desal H, Boulouis G, Berge J, Kazémi A, Pyatigorskaya N, Lecler A, Saleme S, Edjlali-Goujon M, Kerleroux B, Constans JM, Zorn PE, Mathieu M, Baloglu S, Ardellier FD, Willaume T, Brisset JC, Caillard S, Collange O, Mertes PM, Schneider F, Fafi-Kremer S, Ohana M, Meziani F, Meyer N, Helms J, Cotton F. Neurologic and neuroimaging findings in patients with COVID-19: A retrospective multicenter study. Neurology. 2020;95(13):e1868-e1882. doi: 10.1212/WNL.0000000000010112. Epub 2020 Jul 17.	**Included**
26	Argirò R, Cirelli C, Sgreccia A, Del Sette B, Da Ros V. Cerebral hemorrhage related to vein thrombosis in Covid-19 patients in different Italian hospitals: View point for clinical and imaging implications. J Neurol Sci. 2020;416:117023. doi: 10.1016/j.jns.2020.117023. Epub 2020 Jul 10.	Opinion

27	Caballeros Lam M, de la Fuente Villena A, Hernández Hernández A, García de Yébenes M, Bastarrika Alemañ G. Cardiac magnetic resonance characterization of COVID-19 myocarditis. Rev Esp Cardiol (Engl Ed). 2020;73(10):863-864. doi: 10.1016/j.rec.2020.06.018. Epub 2020 Jul 4.	Unfocused
28	Grimaldi S, Lagarde S, Harlé JR, Boucraut J, Guedj E. Autoimmune encephalitis concomitant with SARS-CoV-2 infection: Insight from ^{18}F-FDG PET imaging and neuronal autoantibodies. J Nucl Med. 2020;61(12):1726-1729. doi: 10.2967/jnumed.120.249292. Epub 2020 Jul 24.	Case
29	Guedj E, Million M, Dudouet P, Tissot-Dupont H, Bregeon F, Cammilleri S, Raoult D. ^{18}F-FDG brain PET hypometabolism in post-SARS-CoV-2 infection: substrate for persistent/delayed disorders? Eur J Nucl Med Mol Imaging. 2021;48(2):592-595. doi: 10.1007/s00259-020-04973-x. Epub 2020 Jul 30.	Case
30	Klironomos S, Tzortzakakis A, Kits A, Öhberg C, Kollia E, Ahoromazdae A, Almqvist H, Aspelin Å, Martin H, Ouellette R, Al-Saadi J, Hasselberg M, Haghgou M, Pedersen M, Petersson S, Finnsson J, Lundberg J, Falk Delgado A, Granberg T. Nervous system involvement in Coronavirus Disease 2019: Results from a retrospective consecutive neuroimaging cohort. Radiology. 2020;297(3):E324-E334. doi: 10.1148/radiol.2020202791. Epub 2020 Jul 30.	**Included**
31	Chetrit A, Lechien JR, Ammar A, Chekkoury-Idrissi Y, Distinguin L, Circiu M, Saussez S, Ballester MC, Vasse M, Berradja N, Hans S, Carlier R, Edjlali M. Magnetic resonance imaging of COVID-19 anosmic patients reveals abnormalities of the olfactory bulb: Preliminary prospective study. J Infect. 2020;81(5):816-846. doi: 10.1016/j.jinf.2020.07.028. Epub 2020 Jul 30.	Case
32	O'Shea A, Parakh A, Hedgire S, Lee SI. Multisystem assessment of the imaging manifestations of coagulopathy in hospitalized patients with coronavirus disease (COVID-19). AJR Am J Roentgenol. 2021;216(4):1088-1098. doi: 10.2214/AJR.20.24132. Epub 2021 Feb 3.	**Included**
33	Halsey R, Priftakis D, Mackenzie S, Wan S, Davis LM, Lilburn D, Thornton A, Papathanasiou N, Gnanasegaran G, Bomanji J. COVID-19 in the act: incidental ^{18}F-FDG PET/CT findings in asymptomatic patients and those with symptoms not primarily correlated with COVID-19 during the United Kingdom coronavirus lockdown. Eur J Nucl Med Mol Imaging. 2021;48(1):269-281. doi: 10.1007/s00259-020-04972-y. Epub 2020 Aug 5.	Unfocused
34	Nawabi J, Morotti A, Wildgruber M, Boulouis G, Kraehling H, Schlunk F, Can E, Kniep H, Thomalla G, Psychogios M, Hamm B, Fiehler J, Hanning U, Sporns P. Clinical and imaging characteristics in patients with SARS-CoV-2 infection and acute intracranial hemorrhage. J Clin Med. 2020;9(8):2543. doi: 10.3390/jcm9082543. Online 2020 Aug 6	Unfocused
35	Nicholson P, Gao M, Radovanovic I, Mendes-Pereira V, Hodaie M, Pikula A, Krings T. Not a string, not a tangle, not an aneurysm: Emerging pattern of large parenchymal bleeding in younger patients associated with abnormal vessels on imaging. Clin Neuroradiol. 2021;31(3):653-659. doi: 10.1007/s00062-020-00944-9. Epub 2020 Aug 19.	No COVID-19
36	Escalard S, Chalumeau V, Escalard C, Redjem H, Delvoye F, Hébert S, Smajda S, Ciccio G, Desilles JP, Mazighi M, Blanc R, Maïer B, Piotin M. Early brain imaging shows increased severity of acute ischemic strokes with large vessel occlusion in COVID-19 patients. Stroke. 2020;51(11):3366-3370. doi: 10.1161/STROKEAHA.120.031011. Epub 2020 Aug 19.	Unfocused
37	Kihira S, Delman BN, Belani P, Stein L, Aggarwal A, Rigney B, Schefflein J, Doshi AH, Pawha PS. Imaging Features of Acute Encephalopathy in Patients with COVID-19: A Case Series. AJNR Am J Neuroradiol. 2020;41(10):1804-1808. doi: 10.3174/ajnr.A6715. Epub 2020 Aug 13.	Case
38	Afolabi BI, Idowu BM, Onigbinde SO. Multimodality imaging of coronavirus disease 2019 (COVID-19): a pictorial essay. Pol J Radiol. 2020;85:e387-e393. doi: 10.5114/pjr.2020.97957. eCollection 2020 Jul 30.	Review
39	Dhillon PS, Pointon K, Lenthall R, Nair S, Subramanian G, McConachie N, Izzath W. Regional mechanical thrombectomy imaging protocol in patients presenting with acute ischemic stroke during the COVID-19 pandemic. AJNR Am J Neuroradiol. 2020;41(10):1849-1855. doi: 10.3174/ajnr.A6754. Epub 2020 Aug 20.	Unsuitable
40	Lin E, Lantos JE, Strauss SB, Phillips CD, Campion TR Jr, Navi BB, Parikh NS, Merkler AE, Mir S, Zhang C, Kamel H, Cusick M, Goyal P, Gupta A. Brain imaging of patients with COVID-19: Findings at an academic institution during the height of the outbreak in New York City. AJNR Am J Neuroradiol. 2020;41(11):2001-2008. doi: 10.3174/ajnr.A6793. Epub 2020 Aug 20.	**Included**
41	Goh GX, Tan K, Ang BSP, Wang LF, Tchoyoson Lim CC. Neuroimaging in zoonotic outbreaks affecting the central nervous system: Are we fighting the last war? AJNR Am J Neuroradiol. 2020;41(10):1760-1767. doi: 10.3174/ajnr.A6727. Epub 2020 Aug 20.	Review
42	Mohammad LM, Botros JA, Chohan MO. Necessity of brain imaging in COVID-19 infected patients presenting with acute neurological deficits. Interdiscip Neurosurg. 2020;22:100883. doi: 10.1016/j.inat.2020.100883. Epub 2020 Aug 13.	Case
43	Cheng P, Xu LZ, Zheng WH, Ng RMK, Zhang L, Li LJ, Li WH. Psychometric property study of the posttraumatic stress disorder checklist for DSM-5 (PCL-5) in Chinese healthcare workers during the outbreak of corona virus disease 2019. J Affect Disord. 2020;277:368-374. doi: 10.1016/j.jad.2020.08.038. Epub 2020 Aug 23.	Unfocused
44	Hagerty SL, Wielgosz J, Kraemer J, Nguyen HV, Loew D, Kaysen D. Best practices for approaching cognitive processing therapy and prolonged exposure during the COVID-19 pandemic. J Trauma Stress. 2020;33(5):623-633. doi: 10.1002/jts.22583. Epub 2020 Aug 31.	Unrelated
45	Delorme C, Paccoud O, Kas A, Hesters A, Bombois S, Shambrook P, Boullet A, Doukhi D, Le Guennec L, Godefroy N, Maatoug R, Fossati P, Millet B, Navarro V, Bruneteau G, Demeret S, Pourcher V; CoCo-Neurosciences study group and COVID SMIT PSL study group. COVID-19-related encephalopathy: a case series with brain FDG-positron-emission tomography/computed tomography findings. Eur J Neurol. 2020;27(12):2651-2657. doi: 10.1111/ene.14478. Epub 2020 Sep 22.	Case
46	Yiannakas MC, Schneider T, Yoneyama M, Aforlabi-Logoh I, Prados F, Ciccarelli O, Wheeler-Kingshott CAM. Magnetisation transfer ratio combined with magnetic resonance neurography is feasible in the proximal lumbar plexus using healthy volunteers at 3T. Sci Rep. 2020;10(1):14568. doi: 10.1038/s41598-020-71570-1. Online 2020 Sep 3.	No COVID-19
47	Martínez-Barbero JP, Tomás-Muñoz P, Martínez-Moreno R. Relevance of neuroimaging in publications on COVID-19 and stroke. Neurologia (Engl Ed). 2020;35(9):709-710. doi: 10.1016/j.nrl.2020.07.002. Epub 2020 Jul 22.	Review
48	Sheth KN, Mazurek MH, Yuen MM, Cahn BA, Shah JT, Ward A, Kim JA, Gilmore EJ, Falcone GJ, Petersen N, Gobeske KT, Kaddouh F, Hwang DY, Schindler J, Sansing L, Matouk C, Rothberg J, Sze G, Siner J, Rosen MS, Spudich S, Kimberly WT. Assessment of brain injury using portable, low-field magnetic resonance imaging at the bedside of critically ill patients. JAMA Neurol.2020;78(1):41-7. doi: 10.1001/jamaneurol.2020.3263. Online ahead of print 2020 Sep 8.	Unsuitable
49	Yoon BC, Buch K, Lang M, Applewhite BP, Li MD, Mehan WA Jr, Leslie-Mazwi TM, Rincon SP. Clinical and Neuroimaging Correlation in Patients with COVID-19. AJNR Am J Neuroradiol. 2020;41(10):1791-1796. doi: 10.3174/ajnr.A6717. Epub 2020 Sep 10.	Unsuitable
50	Suri JS, Puvvula A, Biswas M, Majhail M, Saba L, Faa G, Singh IM, Oberleitner R, Turk M, Chadha PS, Johri AM, Sanches JM, Khanna NN, Viskovic K, Mavrogeni S, Laird JR, Pareek G, Miner M, Sobel DW, Balestrieri A, Sfikakis PP, Tsoulfas G, Protogerou A, Misra DP, Agarwal V, Kitas GD, Ahluwalia P, Kolluri R, Teji J, Maini MA, Agbakoba A, Dhanjil SK, Sockalingam M, Saxena A, Nicolaides A, Sharma A, Rathore V, Ajuluchukwu JNA, Fatemi M, Alizad A, Viswanathan V, Krishnan PR, Naidu S. COVID-19 pathways for brain and heart injury in comorbidity patients: A role of medical imaging and artificial intelligence-based COVID severity classification: A review. Comput Biol Med. 2020;124:103960. doi: 10.1016/j.compbiomed.2020.103960. Epub 2020 Aug 14.	Review
51	Cabrera MA, Karamsetty L, Simpson SA. Coronavirus and Its Implications for Psychiatry: A Rapid Review of the Early Literature. Psychosomatics. 2020;61(6):607-615. doi: 10.1016/j.psym.2020.05.018. Epub 2020 May 27.	Review
52	Siddiqui J, Bala F, Sciacca S, Falzon AM, Benger M, Matloob SA, Miller FNAC, Simister RJ, Chatterjee I, Sztriha LK, Davagnanam I, Booth TC. COVID-19 stroke apical lung examination study: A diagnostic and prognostic imaging biomarker in suspected acute stroke. AJNR Am J Neuroradiol. 2021;42(1):138-143. doi: 10.3174/ajnr.A6832. Epub 2020 Sep 17.	Unfocused

53	van Herpen MM, Boeschoten MA, Te Brake H, van der Aa N, Olff M. Mobile insight in risk, resilience, and online referral (MIRROR): Psychometric evaluation of an online self-help test. J Med Internet Res. 2020;22(9):e19716. doi: 10.2196/19716.	Unrelated
54	Naval-Baudin P, Rodriguez Caamaño I, Rubio-Maicas C, Pons-Escoda A, Fernández Viñas MM, Nuñez A, Cardona P, Majos C, Cos M, Calvo N. COVID-19 and ischemic stroke: Clinical and neuroimaging findings. J Neuroimaging. 2021;31(1):62-66. doi: 10.1111/jon.12790. Epub 2020 Sep 28.	Unsuitable
55	Sawlani V, Scotton S, Nader K, Jen JP, Patel M, Gokani K, Denno P, Thaller M, Englezou C, Janjua U, Bowen M, Hoskote C, Veenith T, Hassan-Smith G, Jacob S. COVID-19-related intracranial imaging findings: a large single-centre experience. Clin Radiol. 2021;76(2):108-116. doi: 10.1016/j.crad.2020.09.002. Epub 2020 Sep 15.	**Included**
56	Prasad A, Kataria S, Srivastava S, Lakhani DA, Sriwastava S. Multiple embolic stroke on magnetic resonance imaging of the brain in a COVID-19 case with persistent encephalopathy. Clin Imaging. 2021;69:285-288. doi: 10.1016/j.clinimag.2020.09.010. Epub 2020 Oct 6.	Case
57	Mistraletti G, Gristina G, Mascarin S, Iacobone E, Giubbilo I, Bonfanti S, Fiocca F, Fullin G, Fuselli E, Bocci MG, Mazzon D, Giusti GD, Galazzi A, Negro A, De Iaco F, Gandolfo E, Lamiani G, Del Negro S, Monti L, Salvago F, Di Leo S, Gribaudi MN, Piccinni M, Riccioni L, Giannini A, Livigni S, Maglione C, Vergano M, Marinangeli F, Lovato L, Mezzetti A, Drigo E, Vegni E, Calva S, Aprile A, Losi G, Fontanella L, Calegari G, Ansaloni C, Pugliese FR, Manca S, Orsi L, Moggia F, Scelsi S, Corcione A, Petrini F. How to communicate with families living in complete isolation. BMJ Support Palliat Care. 2020:bmjspcare-2020-002633. doi: 10.1136/bmjspcare-2020-002633. Online ahead of print 2020 Oct 15.	Unrelated
58	Matsubara K, Ibaraki M, Shidahara M, Kinoshita T. Iterative framework for image registration and partial volume correction in brain positron emission tomography. Alzheimer’s Disease Neuroimaging Initiative. Radiol Phys Technol. 2020;13(4):348-357. doi: 10.1007/s12194-020-00591-2. Epub 2020 Oct 19.	No COVID-19
59	Hao F, Tam W, Hu X, Tan W, Jiang L, Jiang X, Zhang L, Zhao X, Zou Y, Hu Y, Luo X, McIntyre RS, Quek T, Tran BX, Zhang Z, Pham HQ, Ho CSH, Ho RCM. A quantitative and qualitative study on the neuropsychiatric sequelae of acutely ill COVID-19 inpatients in isolation facilities. Transl Psychiatry. 2020 Oct 19;10(1):355. doi: 10.1038/s41398-020-01039-2.	Unfocused
60	Agarwal S, Conway J, Nguyen V, Dogra S, Krieger P, Zagzag D, Lewis A, Melmed K, Galetta S, Jain R. Serial imaging of virus-associated necrotizing disseminated acute leukoencephalopathy (VANDAL) in COVID-19. AJNR Am J Neuroradiol. 2021;42(2):279-284. doi: 10.3174/ajnr.A6898. Epub 2020 Oct 22.	Unfocused
61	Rut W, Groborz K, Zhang L, Sun X, Zmudzinski M, Pawlik B, Wang X, Jochmans D, Neyts J, Młynarski W, Hilgenfeld R, Drag M. SARS-CoV-2 Mpro inhibitors and activity-based probes for patient-sample imaging. Nat Chem Biol. 2021;17(2):222-228. doi: 10.1038/s41589-020-00689-z. Epub 2020 Oct 22.	Unfocused
62	Kasa LW, Haast RAM, Kuehn TK, Mushtaha FN, Baron CA, Peters T, Khan AR. Evaluating high spatial resolution diffusion kurtosis imaging at 3T: Reproducibility and quality of fit. J Magn Reson Imaging. 2021;53(4):1175-1187. doi: 10.1002/jmri.27408. Epub 2020 Oct 24.	No COVID-19
63	Fan H, Su P, Huang J, Liu P, Lu H. Multi-band MR fingerprinting (MRF) ASL imaging using artificial-neural-network trained with high-fidelity experimental data. Magn Reson Med. 2021 Apr;85(4):1974-1985. doi: 10.1002/mrm.28560. Epub 2020 Oct 26.	No COVID-19
64	Hatipoglu N, Yazici ZM, Palabiyik F, Gulustan F, Sayin I. Olfactory bulb magnetic resonance imaging in SARS-CoV-2-induced anosmia in pediatric cases. Int J Pediatr Otorhinolaryngol. 2020 Dec;139:110469. doi: 10.1016/j.ijporl.2020.110469. Epub 2020 Oct 24.	Case
65	Shor N, Chougar L, Pyatigorskaya N. MR Imaging of the Olfactory Bulbs in Patients with COVID-19 and Anosmia: How to Avoid Misinterpretation. AJNR Am J Neuroradiol. 2021;42(3):E10-E11. doi: 10.3174/ajnr.A6921. Epub 2020 Oct 29.	Case
66	Rapalino O, Weerasekera A, Moum SJ, Eikermann-Haerter K, Edlow BL, Fischer D, Torrado-Carvajal A, Loggia ML, Mukerji SS, Schaefer PW, Gonzalez RG, Lev MH, Ratai EM. Brain MR spectroscopic findings in 3 consecutive patients with COVID-19: Preliminary observations. AJNR Am J Neuroradiol. 2021;42(1):37-41. doi: 10.3174/ajnr.A6877. Epub 2020 Oct 29.	Case
67	Fontana IC, Bongarzone S, Gee A, Souza DO, Zimmer ER. PET imaging as a tool for assessing COVID-19 brain changes. Trends Neurosci. 2020;43(12):935-938. doi: 10.1016/j.tins.2020.10.010. Epub 2020 Oct 22.	Review
68	Revzin MV, Raza S, Srivastava NC, Warshawsky R, D'Agostino C, Malhotra A, Bader AS, Patel RD, Chen K, Kyriakakos C, Pellerito JS. Multisystem imaging manifestations of COVID-19, Part 2: From cardiac complications to pediatric manifestations. Radiographics. 2020;40(7):1866-1892. doi: 10.1148/rg.2020200195.	Unfocused
69	Choi Y, Lee MK. Neuroimaging findings of brain MRI and CT in patients with COVID-19: A systematic review and meta-analysis. Eur J Radiol. 2020;133:109393. doi: 10.1016/j.ejrad.2020.109393. Epub 2020 Nov 3.	Review
70	Marchini S, Zaurino E, Bouziotis J, Brondino N, Delvenne V, Delhaye M. Study of resilience and loneliness in youth (18-25 years old) during the COVID-19 pandemic lockdown measures. J Community Psychol. 2021;49(2):468-480. doi: 10.1002/jcop.22473. Epub 2020 Nov 10.	Unrelated
71	Kolacz J, Dale LP, Nix EJ, Roath OK, Lewis GF, Porges SW. Adversity history predicts self-reported autonomic reactivity and mental health in US residents during the COVID-19 pandemic. Front Psychiatry. 2020;11:577728. doi: 10.3389/fpsyt.2020.577728. eCollection 2020 Oct 27.	Unrelated
72	Gilan D, Röthke N, Blessin M, Kunzler A, Stoffers-Winterling J, Müssig M, Yuen KSL, Tüscher O, Thrul J, Kreuter F, Sprengholz P, Betsch C, Stieglitz RD, Lieb K. Psychomorbidity, resilience, and exacerbating and protective factors during the SARS-CoV-2 pandemic. Dtsch Arztebl Int. 2020;117(38):625-630. doi: 10.3238/arztebl.2020.0625.	Review
73	Bates A, Rushbrook S, Shapiro E, Grocott M, Cusack R. CovEMERALD: Assessing the feasibility and preliminary effectiveness of remotely delivered Eye Movement Desensitisation and Reprocessing following Covid-19 related critical illness: A structured summary of a study protocol for a randomised controlled trial. Trials. 2020;21(1):929. doi: 10.1186/s13063-020-04805-1.	Protocol
74	Jensen-Kondering U, Neumann A, Margraf NG, Gövert F, Brüggemann N, Schunk D, Bahmer T, Schramm P, Schreiber S, Jansen O. Cerebral imaging in patients with COVID-19 and neurological symptoms: First experience from two university hospitals in Northern Germany. Rofo. 2021;193(6):667-671. doi: 10.1055/a-1265-7209. Epub 2020 Nov 19.	Unsuitable
75	Moonis G, Filippi CG, Kirsch CFE, Mohan S, Stein EG, Hirsch JA, Mahajan A. The spectrum of neuroimaging findings on CT and MRI in adults with COVID-19. AJR Am J Roentgenol. 2021;217(4):959-974. doi: 10.2214/AJR.20.24839. Epub 2020 Nov 25.	Review
76	Zhizhong W, Koenig HG, Yan T, Jing W, Mu S, Hongyu L, Guangtian L. Psychometric properties of the moral injury symptom scale among Chinese health professionals during the COVID-19 pandemic. BMC Psychiatry. 2020;20(1):556. doi: 10.1186/s12888-020-02954-w.	Unrelated
77	Chen B, Chen C, Zheng J, Li R, Xu J. Insights into neuroimaging findings of patients with Coronavirus Disease 2019 presenting with neurological manifestations. Front Neurol. 2020;11:593520. doi: 10.3389/fneur.2020.593520. eCollection 2020 Nov 9.	Review
78	Katal S, Gholamrezanezhad A. Neuroimaging findings in COVID-19: A narrative review. Neurosci Lett. 2021;742:135529. doi: 10.1016/j.neulet.2020.135529. Epub 2020 Nov 25.	Review
79	Lersy F, Benotmane I, Helms J, Collange O, Schenck M, Brisset JC, Chammas A, Willaume T, Lefebvre N, Solis M, Hansmann Y, Fabacher T, Caillard S, Mertes PM, Pottecher J, Schneider F, Meziani F, Fafi-Kremer S, Kremer S. Cerebrospinal fluid features in patients with Coronavirus Disease 2019 and neurological manifestations: Correlation with brain magnetic resonance imaging findings in 58 patients. J Infect Dis. 2021;223(4):600-609. doi: 10.1093/infdis/jiaa745.	Unsuitable
80	Lee N, Lee HJ. South Korean nurses' experiences with patient care at a COVID-19-designated hospital: Growth after the frontline battle against an infectious disease pandemic. Int J Environ Res Public Health. 2020;17(23):9015. doi: 10.3390/ijerph17239015.	Unrelated

81	Shahid M, Rinne JO, Scheinin M, Virta J, Marjamäki P, Solin O, Arponen E, Sallinen J, Kuokkanen K, Rouru J. Application of the PET ligand [^{11}C]ORM-13070 to examine receptor occupancy by the α_{2C}-adrenoceptor antagonist ORM-12741: translational validation of target engagement in rat and human brain. EJNMMI Res. 2020;10(1):152. doi: 10.1186/s13550-020-00741-y.	Animal
82	Jagesar RR, Roozen MC, van der Heijden I, Ikani N, Tyborowska A, Penninx BWJH, Ruhe HG, Sommer IEC, Kas MJ, Vorstman JAS. Digital phenotyping and the COVID-19 pandemic: Capturing behavioral change in patients with psychiatric disorders. Eur Neuropsychopharmacol. 2021;42:115-120. doi: 10.1016/j.euroneuro.2020.11.012. Epub 2020 Nov 19.	Unrelated
83	Drenthen GS, Backes WH, Jansen JFA. Estimating myelin-water content from anatomical and diffusion images using spatially undersampled myelin-water imaging through machine learning. Neuroimage. 2021;226:117626. doi: 10.1016/j.neuroimage.2020.117626. Epub 2020 Dec 8.	Unrelated
84	Elkhaled W, Ben Abid F, Akhtar N, Abukamar MR, Ibrahim WH. A 23-year-old man with SARS-CoV-2 infection who presented with auditory hallucinations and imaging findings of cytotoxic lesions of the corpus callosum (CLOCC). Am J Case Rep. 2020;21:e928798. doi: 10.12659/AJCR.928798.	Case
85	Lindan CE, Mankad K, Ram D, Kociolek LK, Silvera VM, Boddaert N, Stivaros SM, Palasis S; ASPNR PECOBIG Collaborator Group. Neuroimaging manifestations in children with SARS-CoV-2 infection: a multinational, multicentre collaborative study. Lancet Child Adolesc Health. 2021;5(3):167-177. doi: 10.1016/S2352-4642(20)30362-X. Epub 2020 Dec 16.	**Included**
86	Crowe S, Howard AF, Vanderspank-Wright B, Gillis P, McLeod F, Penner C, Haljan G. The effect of COVID-19 pandemic on the mental health of Canadian critical care nurses providing patient care during the early phase pandemic: A mixed method study. Intensive Crit Care Nurs. 2021;63:102999. doi: 10.1016/j.iccn.2020.102999. Epub 2020 Dec 11.	Unrelated
87	Vogrig A, Gigli GL, Bnà C, Morassi M. Stroke in patients with COVID-19: Clinical and neuroimaging characteristics. Neurosci Lett. 2021;743:135564. doi: 10.1016/j.neulet.2020.135564. Epub 2020 Dec 19.	Review
88	Lai X, Liu J, Zhang T, Feng L, Jiang P, Kang L, Liu Q, Gao Y. Clinical, laboratory and imaging predictors for critical illness and mortality in patients with COVID-19: protocol for a systematic review and meta-analysis. BMJ Open. 2020;10(12):e039813. doi: 10.1136/bmjopen-2020-039813.	Review
89	Elhai JD, McKay D, Yang H, Minaya C, Montag C, Asmundson GJG. Health anxiety related to problematic smartphone use and gaming disorder severity during COVID-19: Fear of missing out as a mediator. Hum Behav Emerg Technol. 2021 Jan;3(1):137-146. doi: 10.1002/hbe2.227. Epub 2020 Nov 23.	Unrelated
90	Chowdhary A, Subedi R, Tandon M, Wen S, Patel J, Kataria S, Peterson S, Gwinn R, Elkhooly M, Prasad A, Nirwan L, Adcock A, Sriwastava S. Relevance and clinical significance of magnetic resonance imaging of neurological manifestations in COVID-19: A systematic review of case reports and case series. Brain Sci. 2020;10(12):1017. doi: 10.3390/brainsci10121017.	Review
91	Ciorba A, Hatzopoulos S, Cogliandolo C, Bianchini C, Renna M, Pelucchi S, Skarżyński PH, Skarzynska M, Campioni P, Cittanti C, Carnevale A, Giganti M, Perrucci L. Functional magnetic resonance imaging in the olfactory perception of the same stimuli. Life (Basel). 2020;11(1):11. doi: 10.3390/life11010011.	Unrelated
92	Turpin J, Unadkat P, Thomas J, Kleiner N, Khazanehdari S, Wanchoo S, Samuel K, Moclair BO, Black K, Dehdashti AR, Narayan RK, Temes R, Schulder M. Portable magnetic resonance imaging for ICU patients. Crit Care Explor. 2020;2(12):e0306. doi: 10.1097/CCE.0000000000000306. eCollection 2020 Dec 21Dec.	Unrelated
93	Giusti L, Salza A, Mammarella S, Bianco D, Ussorio D, Casacchia M, Roncone R. #Everything will be fine. Duration of home confinement and "all-or-nothing" cognitive thinking style as predictors of traumatic distress in young university students on a digital platform during the COVID-19 Italian lockdown. Front Psychiatry. 2020;11:574812. doi: 10.3389/fpsyt.2020.574812. eCollection 2020 Dec 15.	Unrelated
94	McGregor G, Sandhu H, Bruce J, Sheehan B, McWilliams D, Yeung J, Jones C, Lara B, Smith J, Ji C, Fairbrother E, Ennis S, Heine P, Alleyne S, Guck J, Padfield E, Potter R, Mason J, Lall R, Seers K, Underwood M. Rehabilitation Exercise and psycholoGical support After covid-19 InfectioN' (REGAIN): a structured summary of a study protocol for a randomised controlled trial. Trials. 2021;22(1):8. doi: 10.1186/s13063-020-04978-9.	Protocol
95	Tuma RL, Guedes BF, Carra R, Iepsen B, Rodrigues J, Camelo-Filho AE, Kubota G, Ferrari M, Studart-Neto A, Oku MH, Terrim S, Lopes CCB, Passos Neto CEB, Fiorentino MD, Souza JCC, Baima JPS, Silva T, Perissinotti I, Martin MDGM, Gonçalves M, Fortini I, Smid J, Adoni T, Lucato L, Nitrini R, Gomes H, Castro LH. Clinical, cerebrospinal fluid, and neuroimaging findings in COVID-19 encephalopathy: a case series. Neurol Sci. 2021;42(2):479-489. doi: 10.1007/s10072-020-04946-w. Epub 2021 Jan 7.	Case
96	Orman G, Desai NK, Kralik SF, Meoded A, Seghers VJ, Annapragada AV, Huisman TAGM. Neuroimaging offers low yield in children positive for SARS-CoV-2. AJNR Am J Neuroradiol. 2021;42(5):951-954. doi: 10.3174/ajnr.A7022. Epub 2021 Jan 7.	**Included**
97	Rapalino O, Pourvaziri A, Maher M, Jaramillo-Cardoso A, Edlow BL, Conklin J, Huang S, Westover B, Romero JM, Halpern E, Gupta R, Pomerantz S, Schaefer P, Gonzalez RG, Mukerji SS, Lev MH. Clinical, imaging, and lab correlates of severe COVID-19 leukoencephalopathy. AJNR Am J Neuroradiol. 2021;42(4):632-638. doi: 10.3174/ajnr.A6966. Epub 2021 Jan 7.	**Included**
98	Ladopoulos T, Zand R, Shahjouei S, Chang JJ, Motte J, Charles James J, Katsanos AH, Kerro A, Farahmand G, Vaghefi Far A, Rahimian N, Ebrahimzadeh SA, Abedi V, Papathanasiou M, Labedi A, Schneider R, Lukas C, Tsiodras S, Tsivgoulis G, Krogias C. COVID-19: Neuroimaging features of a pandemic. J Neuroimaging. 2021;31(2):228-243. doi: 10.1111/jon.12819. Epub 2021 Jan 9.	Review
99	Fenlon EP, III, Chen S, Ruzal-Shapiro CB, Jaramillo D, Maddocks ABR. Extracardiac imaging findings in COVID-19-associated multisystem inflammatory syndrome in children. Pediatr Radiol. 2021;51(5):831-839. doi: 10.1007/s00247-020-04929-1. Epub 2021 Jan 12.	**Unsuitable**
100	Hoorelbeke K, Sun X, Koster EHW, Dai Q. Connecting the dots: A network approach to post-traumatic stress symptoms in Chinese healthcare workers during the peak of the Coronavirus Disease 2019 outbreak. Stress Health. 2021;37(4):692-705. doi: 10.1002/smi.3027. Epub 2021 Feb 4.	No neuroim.
101	Elavsky S, Jandačková V, Knapová L, Vašendová V, Sebera M, Kaštovská B, Blaschová D, Kühnová J, Cimler R, Vilímek D, Bosek T, Koenig J, Jandačka D. Physical activity in an air-polluted environment: behavioral, psychological and neuroimaging protocol for a prospective cohort study (Healthy Aging in Industrial Environment study - Program 4). BMC Public Health. 2021;21(1):126. doi: 10.1186/s12889-021-10166-4.	Unrelated
102	Kas A, Soret M, Pyatigoskaya N, Habert MO, Hesters A, Le Guennec L, Paccoud O, Bombois S, Delorme C; on the behalf of CoCo-Neurosciences study group and COVID SMIT PSL study group. The cerebral network of COVID-19-related encephalopathy: a longitudinal voxel-based ^{18}F-FDG-PET study. Eur J Nucl Med Mol Imaging. 2021;48(8):2543-2557. doi: 10.1007/s00259-020-05178-y. Epub 2021 Jan 15.	**Included**
103	Vazquez C, Valiente C, García FE, Contreras A, Peinado V, Trucharte A, Bentall RP. Post-traumatic growth and stress-related responses during the COVID-19 pandemic in a national representative sample: The role of positive core beliefs about the world and others. J Happiness Stud. 2021;22(7):2915-2935. doi: 10.1007/s10902-020-00352-3. Epub 2021 Jan 11.	Unrelated
104	Corrêa DG, Hygino da Cruz LC Jr, Lopes FCR, Rangel CC, de Araújo Henriques Tessarollo AL, Coelho KCG, Brandão RZ, Novis RAF, Novis SSC, Silveira R, Carvalho VT, Nascimento OJM. Magnetic resonance imaging features of COVID-19-related cranial nerve lesions. J Neurovirol. 2021;27(1):171-177. doi: 10.1007/s13365-020-00934-0. Epub 2021 Jan 18.	Unrelated
105	Rosebrock L, Černis E, Lambe S, Waite F, Rek S, Petit A, Ehlers A, Clark DM, Freeman D. Catastrophic cognitions about coronavirus: the Oxford psychological investigation of coronavirus questionnaire [TOPIC-Q]. Psychol Med. 2021:1-10. doi: 10.1017/S0033291721000283. Online ahead of print 2021 Jan 22.	Unrelated

106	Gagliardi RJ, Domingues R, Cukier C, Mendes MF, Gagliardi VDB, de Oliveira FTM. Neurological manifestations of COVID-19 and the importance of magnetic resonance imaging. CNS Neurol Disord Drug Targets. 2021;20(5):390-391. doi: 10.2174/1871527320666210122093458.	Opinion
107	Ismail II, Gad KA. Absent blood oxygen level-dependent functional magnetic resonance imaging activation of the orbitofrontal cortex in a patient with persistent cacosmia and cacogeusia after COVID-19 infection. JAMA Neurol. 2021;78(5):609-610. doi: 10.1001/jamaneurol.2021.0009.	Case
108	Conklin J, Frosch MP, Mukerji SS, Rapalino O, Maher MD, Schaefer PW, Lev MH, Gonzalez RG, Das S, Champion SN, Magdamo C, Sen P, Harrold GK, Alabsi H, Normandin E, Shaw B, Lemieux JE, Sabeti PC, Branda JA, Brown EN, Westover MB, Huang SY, Edlow BL. Susceptibility-weighted imaging reveals cerebral microvascular injury in severe COVID-19. J Neurol Sci. 2021;421:117308. doi: 10.1016/j.jns.2021.117308. Epub 2021 Jan 15.	**Included**
109	Guedj E, Campion JY, Dudouet P, Kaphan E, Bregeon F, Tissot-Dupont H, Guis S, Barthelemy F, Habert P, Ceccaldi M, Million M, Raoult D, Cammilleri S, Eldin C. ^{18}F-FDG brain PET hypometabolism in patients with long COVID. Eur J Nucl Med Mol Imaging. 2021;48(9):2823-2833. doi: 10.1007/s00259-021-05215-4. Epub 2021 Jan 26.	**Included**
110	Dieckmeyer M, Roy AG, Senapati J, Wachinger C, Grundl L, Döpfert J, Bertran PF, Lemke A, Zimmer C, Kirschke JS, Hedderich DM. Effect of MRI acquisition acceleration via compressed sensing and parallel imaging on brain volumetry. MAGMA. 2021;34(4):487-497. doi: 10.1007/s10334-020-00906-9. Epub 2021 Jan 27.	Unrelated
111	Hill AR, Spencer-Segal JL. Glucocorticoids and the brain after critical illness. Endocrinology. 2021;162(3):bqaa242. doi: 10.1210/endocr/bqaa242.	Review
112	Ellena AM, Aresi G, Marta E, Pozzi M. Post-traumatic growth dimensions differently mediate the relationship between national identity and interpersonal trust among young adults: A study on COVID-19 crisis in Italy. Front Psychol. 2021;11:576610. doi: 10.3389/fpsyg.2020.576610. eCollection 2020 Jan 15.	Unrelated
113	Busch IM, Moretti F, Mazzi M, Wu AW, Rimondini M. What we have learned from two decades of epidemics and pandemics: A systematic review and meta-analysis of the psychological burden of frontline healthcare workers. Psychother Psychosom. 2021;90(3):178-190. doi: 10.1159/000513733. Epub 2021 Feb 1.	Review
114	Gao J, Liu Q, Zhou C, Zhang W, Wan Q, Hu C, Gu Z, Liang D, Liu X, Yang Y, Zheng H, Hu Z, Zhang N. An improved patch-based regularization method for PET image reconstruction. Quant Imaging Med Surg. 2021;11(2):556-570. doi: 10.21037/qims-20-19.	Unrelated
115	de Graaf MA, Antoni ML, Ter Kuile MM, Arbous MS, Duinisveld AJF, Feltkamp MCW, Groeneveld GH, Hinnen SCH, Janssen VR, Lijfering WM, Omara S, Postmus PE, Ramai SRS, Rius-Ottenheim N, Schalij MJ, Schiemanck SK, Smid L, Stöger JL, Visser LG, de Vries JJC, Wijngaarden MA, Geelhoed JJM, Roukens AHE. Short-term outpatient follow-up of COVID-19 patients: A multidisciplinary approach. EClinicalMedicine. 2021;32:100731. doi: 10.1016/j.eclinm.2021.100731. Epub 2021 Jan 28.	No neuroim.
116	Büttner L, Bauknecht HC, Fleckenstein FN, Kahn J, Tietze A, Bohner G, Siebert E. Neuroimaging findings in conjunction with severe COVID-19. Rofo. 2021;193(7):822-829. doi: 10.1055/a-1345-9784. Epub 2021 Feb 3.	**Included**
117	Nooner KB, Chung T, Feldstein Ewing SW, Brumback T, Arwood Z, Tapert SF, Brown SA, Cottler L. Retaining adolescent and young adult participants in research during a pandemic: Best practices from two large-scale developmental neuroimaging studies (NCANDA and ABCD). Front Behav Neurosci. 2021;14:597902. doi: 10.3389/fnbeh.2020.597902. eCollection 2020 Jan 18.	Unrelated
118	Gimenez-Llort L, Alveal-Mellado D. Digging signatures in 13-Month-Old 3xTg-AD mice for Alzheimer's disease and its disruption by isolation despite social life since they were born. Front Behav Neurosci. 2021;14:611384. doi: 10.3389/fnbeh.2020.611384. eCollection 2020 Jan 18.	Animal
119	Lang M, Li MD, Jiang KZ, Yoon BC, Mendoza DP, Flores EJ, Rincon SP, Mehan WA Jr, Conklin J, Huang SY, Lang AL, Giao DM, Leslie-Mazwi TM, Kalpathy-Cramer J, Little BP, Buch K. Severity of chest imaging is correlated with risk of acute neuroimaging findings among patients with COVID-19. AJNR Am J Neuroradiol. 2021;42(5):831-837. doi: 10.3174/ajnr.A7032. Epub 2021 Feb 4.	Unfocused
120	Walsh C, Tafforeau P, Wagner WL, Jafree DJ, Bellier A, Werlein C, Kühnel MP, Boller E, Walker-Samuel S, Robertus JL, Long DA, Jacob J, Marussi S, Brown E, Holroyd N, Jonigk DD, Ackermann M, Lee PD. Multiscale three-dimensional imaging of intact human organs down to the cellular scale using hierarchical phase-contrast tomography. bioRxiv. 2021:2021.02.03.429481. doi: 10.1101/2021.02.03.429481. Preprint 2021 Feb 3.	Unrelated
121	Meo SA, Abukhalaf AA, Alomar AA, Al-Hussain F. Magnetic Resonance Imaging (MRI) and neurological manifestations in SARS-CoV-2 patients. Eur Rev Med Pharmacol Sci. 2021;25(2):1101-1108. doi: 10.26355/eurrev_202101_24681.	Review
122	Yousefi-Koma A, Haseli S, Bakhshayeshkaram M, Raad N, Karimi-Galougahi M. Multimodality imaging with PET/CT and MRI reveals hypometabolism in tertiary olfactory cortex in parosmia of COVID-19. Acad Radiol. 2021;28(5):749-751. doi: 10.1016/j.acra.2021.01.031. Epub 2021 Feb 3.	Opinion
123	Geng S, Zhou Y, Zhang W, Lou A, Cai Y, Xie J, Sun J, Zhou W, Liu W, Li X. The influence of risk perception for COVID-19 pandemic on posttraumatic stress disorder in healthcare workers: A survey from four designated hospitals. Clin Psychol Psychother. 2021;28(5):1146-1159. doi: 10.1002/cpp.2564. Epub 2021 Feb 16.	No neuroim.
124	Aftan MK, Badrawi N, Abdulghaffar S, Abedzadeh AA, Albastaki U, Raman LG, Alzeena EFG. Pure testicular choriocarcinoma Cannonball metastases as a presenting imaging feature: A case report and a review of literature. Radiol Case Rep. 2021;16(4):923-928. doi: 10.1016/j.radcr.2021.01.053. eCollection 2021 Apr Feb 9.	Unrelated
125	Kerro A. Encephalopathy with progression to posterior reversible encephalopathy pattern in a patient with COVID-19: clinical, imaging findings and follow-up. BMJ Case Rep. 2021;14(2):e241240. doi: 10.1136/bcr-2020-241240.	Case
126	Thurnher MM, Boban J, Röggla M, Staudinger T. Distinct pattern of microsusceptibility changes on brain magnetic resonance imaging (MRI) in critically ill patients on mechanical ventilation/oxygenation. Neuroradiology. 2021;63(10):1651-1658. doi: 10.1007/s00234-021-02663-5. Epub 2021 Mar 1.	**Included**
127	Khandwala K, Mubarak F, Ahmad MN. Imaging Patterns of Encephalopathy in Patients with COVID-19. J Coll Physicians Surg Pak. 2021;30(1):S42-S45. doi: 10.29271/jcpsp.2021.01.S42.	Review
128	Yin Q, Chen A, Song X, Deng G, Dong W. Risk perception and PTSD symptoms of medical staff combating against COVID-19: A PLS structural equation model. Front Psychiatry. 2021;12:607612. doi: 10.3389/fpsyt.2021.607612. eCollection 2021 Feb 15.	Unrelated
129	Piqueras JA, Garcia-Olcina M, Rivera-Riquelme M, Martinez-Gonzalez AE, Cuijpers P. DetectaWeb-Distress Scale: A global and multidimensional web-based screener for emotional disorder symptoms in children and adolescents. Front Psychol. 2021;12:627604. doi: 10.3389/fpsyg.2021.627604. eCollection 2021 Feb 15.	Unrelated
130	Durcan E, Turan S, Sahin S, Sulu C, Taze SS, Kavla Y, Ozkaya HM, Kadioglu P. Psychosocial effects and clinic reflections of the COVID-19 outbreak in patients with acromegaly and Cushing's disease. Pituitary. 2021;24(4):589-599. doi: 10.1007/s11102-021-01136-5. Epub 2021 Mar 5.	Unrelated
131	Agarwal S, Melmed K, Dogra S, Jain R, Conway J, Galetta S, Lewis A. Increase in ventricle size and the evolution of white matter changes on serial imaging in critically ill patients with COVID-19. Neurocrit Care. 2021;35(2):491-500. doi: 10.1007/s12028-021-01207-2. Epub 2021 Mar 5.	**Included**
132	Chammas A, Namer IJ, Lersy F, Kremer S, Bund C. Inferior colliculus's hypermetabolism: A new finding on brain FDG PET and perfusion MRI in a patient with COVID-19. Clin Nucl Med. 2021;46(5):413-414. doi: 10.1097/RLU.0000000000003592.	Case

133	Sollini M, Morbelli S, Ciccarelli M, Cecconi M, Aghemo A, Morelli P, Chiola S, Gelardi F, Chiti A. Long COVID hallmarks on [^{18}F]FDG-PET/CT: a case-control study. Eur J Nucl Med Mol Imaging. 2021;48(10):3187-3197. doi: 10.1007/s00259-021-05294-3. Epub 2021 Mar 7.	FC
134	Rehmani R, Segan S, Maddika SR, Lei YW, Broka A. Spectrum of neurologic & neuroimaging manifestation in COVID-19. Brain Behav Immun Health. 2021;13:100238. doi: 10.1016/j.bbih.2021.100238. Epub 2021 Mar 3.	Case
135	Mira JJ, Cobos A, Martínez García O, Bueno Domínguez MJ, Astier-Peña MP, Pérez Pérez P, Carrillo I, Guilabert M, Perez-Jover V, Fernandez C, Vicente MA, Lahera-Martin M, Silvestre Busto C, Lorenzo Martínez S, Sanchez Martinez A, Martin-Delgado J, Mula A, Marco-Gomez B, Abad Bouzan C, Aibar-Remon C, Aranaz-Andres J; SARS-CoV-2 Second Victims Working Group. An acute stress scale for health care professionals caring for patients with COVID-19: Validation study. JMIR Form Res. 2021;5(3):e27107. doi: 10.2196/27107.	Unrelated
136	Mahammedi A, Ramos A, Bargalló N, Gaskill M, Kapur S, Saba L, Carrete H Jr, Sengupta S, Salvador E, Hilario A, Revilla Y, Sanchez M, Perez-Nuñez M, Bachir S, Zhang B, Oleaga L, Sergio J, Koren L, Martin-Medina P, Wang L, Benegas M, Ostos F, Gonzalez-Ortega G, Calleja P, Udstuen G, Williamson B, Khandwala V, Chadalavada S, Woo D, Vagal A. Brain and lung imaging correlation in patients with COVID-19: Could the severity of lung disease reflect the prevalence of acute abnormalities on neuroimaging? A global multicenter observational study. AJNR Am J Neuroradiol. 2021;42(6):1008-1016. doi: 10.3174/ajnr.A7072. Epub 2021 Mar 11.	**Included**
137	Lambrecq V, Hanin A, Munoz-Musat E, Chougar L, Gassama S, Delorme C, Cousyn L, Borden A, Damiano M, Frazzini V, Huberfeld G, Landgraf F, Nguyen-Michel VH, Pichit P, Sangare A, Chavez M, Morélot-Panzini C, Morawiec E, Raux M, Luyt CE, Rufat P, Galanaud D, Corvol JC, Lubetzki C, Rohaut B, Demeret S, Pyatigorskaya N, Naccache L, Navarro V; Cohort COVID-19 Neurosciences (CoCo Neurosciences) Study Group. Association of clinical, biological, and brain magnetic resonance imaging findings with electroencephalographic findings for patients with COVID-19. JAMA Netw Open. 2021;4(3):e211489. doi: 10.1001/jamanetworkopen.2021.1489.	**Included**
138	Yan K, Xiao FF, Jiang YW, Xiao TT, Zhang DJ, Yuan WH, Shao JB, Cheng GQ, Zeng LK. Effects of SARS-CoV-2 infection on neuroimaging and neurobehavior in neonates. World J Pediatr. 2021;17(2):171-179. doi: 10.1007/s12519-021-00423-2. Epub 2021 Mar 19.	**Included**
139	Zhao Y, Jiang Z, Guo S, Wu P, Lu Q, Xu Y, Liu L, Su S, Shi L, Que J, Sun Y, Sun Y, Deng J, Meng S, Yan W, Yuan K, Sun S, Yang L, Ran M, Kosten TR, Strang J, Lu Y, Huang G, Lu L, Bao Y, Shi J. Association of symptoms of attention deficit and hyperactivity with problematic internet use among university students in Wuhan, China during the COVID-19 pandemic. J Affect Disord. 2021;286:220-227. doi: 10.1016/j.jad.2021.02.078. Epub 2021 Mar 5.	Unrelated
140	Uginet M, Breville G, Hofmeister J, Machi P, Lalive PH, Rosi A, Fitsiori A, Vargas MI, Assal F, Allali G, Lovblad KO. Cerebrovascular complications and vessel wall imaging in COVID-19 encephalopathy-A pilot study. Clin Neuroradiol. 2022;32(1):287-293. doi: 10.1007/s00062-021-01008-2. Epub 2021 Mar 26.	**Included**
141	Cottin M, Hernández C, Núñez C, Labbé N, Quevedo Y, Davanzo A, Behn A. "What if we get sick?": Spanish adaptation and validation of the Fear of Illness and Virus Evaluation Scale in a non-clinical sample exposed to the COVID-19 pandemic. Front Psychol. 2021;12:590283. doi: 10.3389/fpsyg.2021.590283. eCollection 2021 Mar 11.	Unrelated
142	Boettcher AN, Hammoud DA, Weinberg JB, Agarwal P, Mendiratta-Lala M, Luker GD. Cancer imaging and patient care during the COVID-19 pandemic. Radiol Imaging Cancer. 2020;2(6):e200058. doi: 10.1148/rycan.2020200058. eCollection 2020 Nov Nov 13.	Review
143	Ullah I, Prévost J, Ladinsky MS, Stone H, Lu M, Anand SP, Beaudoin-Bussières G, Symmes K, Benlarbi M, Ding S, Gasser R, Fink C, Chen Y, Tauzin A, Goyette G, Bourassa C, Medjahed H, Mack M, Chung K, Wilen CB, Dekaban GA, Dikeakos JD, Bruce EA, Kaufmann DE, Stamatatos L, McGuire AT, Richard J, Pazgier M, Bjorkman PJ, Mothes W, Finzi A, Kumar P, Uchil PD. Live imaging of SARS-CoV-2 infection in mice reveals neutralizing antibodies require Fc function for optimal efficacy. bioRxiv. 2021:2021.03.22.436337. doi: 10.1101/2021.03.22.436337. Preprint 2021 Jun 28.	Animal
144	Banerjee D, Sathyanarayana Rao TS, Kallivayalil RA, Javed A. Psychosocial framework of resilience: Navigating needs and adversities during the pandemic, a qualitative exploration in the Indian frontline physicians. Front Psychol. 2021;12:622132. doi: 10.3389/fpsyg.2021.622132. eCollection 2021 Mar 16.	Unrelated
145	Mahmoudi H, Saffari M, Movahedi M, Sanaeinasab H, Rashidi-Jahan H, Pourgholami M, Poorebrahim A, Barshan J, Ghiami M, Khoshmanesh S, Potenza MN, Lin CY, Pakpour AH. A mediating role for mental health in associations between COVID-19-related self-stigma, PTSD, quality of life, and insomnia among patients recovered from COVID-19. Brain Behav. 2021;11(5):e02138. doi: 10.1002/brb3.2138. Epub 2021 Apr 3.	No neuroim.
146	Fontana IC, Souza DG, Pellerin L, Souza DO, Zimmer ER. About the source and consequences of 18F-FDG brain PET hypometabolism in short and long COVID-19. Eur J Nucl Med Mol Imaging. 2021;48(9):2674-2675. doi: 10.1007/s00259-021-05342-y. Epub 2021 Apr 4.	Opinion
147	Kawthalkar AS, Somani SD, Bhalde RP. Where have the others gone? Impact of lockdown during COVID-19 pandemic on patient populations undergoing Computed Tomography imaging in a public tertiary care hospital in India. Indian J Radiol Imaging. 2021;31(Suppl 1):S134-S138. doi: 10.4103/ijri.IJRI_424_20. Epub 2021 Jan 23.	Unrelated
148	Yin G, Li L, Lu S, Yin Y, Su Y, Zeng Y, Luo M, Ma M, Zhou H, Orlandini L, Yao D, Liu G, Lang J. An efficient primary screening of COVID-19 by serum Raman spectroscopy. J Raman Spectrosc. 2021;52(5):949-958. doi: 10.1002/jrs.6080. Epub 2021 Feb 19.	Unrelated
149	Liu X, Ju X, Liu X. The relationship between resilience and intent to stay among Chinese nurses to support Wuhan in managing COVID-19: The serial mediation effect of post-traumatic growth and perceived professional benefits. Nurs Open. 2021;8(5):2866-2876. doi: 10.1002/nop2.874. Epub 2021 Apr 7.	Unrelated
150	Wang Y, Yang X, Chen H, Xu Y. Effect of smartphone app on post-traumatic stress disorder in COVID-19 convalescent patients: A protocol for systematic review and meta-analysis. Medicine (Baltimore). 2021;100(14):e25479. doi: 10.1097/MD.0000000000025479.	Review
151	Velotti P, Civilla C, Rogier G, Beomonte Zobel S. A fear of COVID-19 and PTSD symptoms in pathological personality: The mediating effect of dissociation and emotion dysregulation. Front Psychiatry. 2021;12:590021. doi: 10.3389/fpsyt.2021.590021. eCollection 2021 Mar 23.	No neuroim.
152	Yang X, Yip BHK, Mak ADP, Zhang D, Lee EKP, Wong SYS. The differential effects of social media on depressive symptoms and suicidal ideation among the younger and older adult population in Hong Kong during the COVID-19 pandemic: Population-based cross-sectional survey study. JMIR Public Health Surveill. 2021;7(5):e24623. doi: 10.2196/24623.	Unrelated
153	Hu T, Wang Y, Lin L, Tang W. The mediating role of daytime sleepiness between problematic smartphone use and post-traumatic symptoms in COVID-19 home-refined adolescents. Child Youth Serv Rev. 2021;126:106012. doi: 10.1016/j.childyouth.2021.106012. Epub 2021 Apr 8.	Unrelated
154	Kansagra AP, Goyal MS, Hamilton S, Albers GW. Stroke imaging utilization according to age and severity during the COVID-19 pandemic. Radiology. 2021;300(3):E342-E344. doi: 10.1148/radiol.2021204716. Epub 2021 Apr 13.	Unrelated
155	Astill Wright L, Gnanapragasam S, Downes AJ, Bisson JI. Managing COVID-19 related distress in primary care: principles of assessment and management. BMC Fam Pract. 2021;22(1):73. doi: 10.1186/s12875-021-01399-8.	Review
156	Mavrogeni SI, Kolovou G, Tsirimpis V, Kafetzis D, Tsolas G, Fotis L. The importance of heart and brain imaging in children and adolescents with Multisystem Inflammatory Syndrome in Children (MIS-C). Rheumatol Int. 2021;41(6):1037-1044. doi: 10.1007/s00296-021-04845-z. Epub 2021 Apr 17.	Unrelated
157	Jegatheeswaran V, Chan MWK, Chakrabarti S, Fawcett A, Chen YA. Neuroimaging findings of hospitalized Covid-19 patients: A Canadian retrospective observational study. Can Assoc Radiol J. 2022;73(1):179-186. doi: 10.1177/08465371211002815. Epub 2021 Apr 21.	**Included**

158	Morassi M, Palmerini F, Nici S, Magni E, Savelli G, Guerra UP, Chieregato M, Morbelli S, Vogrig A. SARS-CoV-2-related encephalitis with prominent parkinsonism: clinical and FDG-PET correlates in two patients. J Neurol. 2021;268(11):3980-3987. doi: 10.1007/s00415-021-10560-3. Epub 2021 Apr 21.	Case
159	Rosa RG, Robinson CC, Veiga VC, Cavalcanti AB, Azevedo LCP, Machado FR, Berwanger O, Avezum Á, Lopes RD, Lisboa TC, Teixeira C, Zampieri FG, Tomazini BM, Kawano-Dourado L, Schneider D, Souza D, Santos RDRMD, Silva SSD, Trott G, Gimenes BDP, Souza AP, Barroso BM, Costa LS, Brognoli LG, Pelliccioli MP, Studier NDS, Schardosim RFC, Haubert TA, Pallaoro VEL, Oliveira DM, Velho PI, Medeiros GS, Gazzana MB, Zavascki AP, Pitrez PM, Oliveira RP, Polanczyk CA, Nasi LA, Hammes LS, Falavigna M. Quality of life and long-term outcomes after hospitalization for COVID-19: Protocol for a prospective cohort study (Coalition VII). Rev Bras Ter Intensiva. 2021;33(1):31-37. doi: 10.5935/0103-507X.20210003.	Protocol
160	Diaz A, Baweja R, Bonatakis JK, Baweja R. Global health disparities in vulnerable populations of psychiatric patients during the COVID-19 pandemic. World J Psychiatry. 2021;11(4):94-108. doi: 10.5498/wjp.v11.i4.94. eCollection 2021 Apr 19.	Review
161	Martin PJ, Felker M, Radhakrishnan R. MR Imaging Findings in a Neonate With COVID -19-Associated Encephalitis.Pediatr Neurol. 2021;119:48-49. doi: 10.1016/j.pediatrneurol.2021.02.012. Epub 2021 Mar 9.	Case
162	Neuroimaging Patterns in Patients with COVID-19-Associated Neurological Complications: A Review. Garg RK, Paliwal VK, Malhotra HS, Sharma PK. Neurol India. 2021;69(2):260-271. doi: 10.4103/0028-3886.314531.	Review
163	Caro-Domínguez P, Navallas M, Riaza-Martin L, Ghadimi Mahani M, Ugas Charcape CF, Valverde I, D'Arco F, Toso S, Shelmerdine SC, van Schuppen J, Secinaro A, Gräfe D, Camacho M, Neth O, Goo HW, Kellenberger CJ. Imaging findings of multisystem inflammatory syndrome in children associated with COVID-19. Pediatr Radiol. 2021;51(9):1608-1620. doi: 10.1007/s00247-021-05065-0. Epub 2021 Apr 27.	Unrelated
164	Mogensen MA, Wangaryattawanich P, Hartman J, Filippi CG, Hippe DS, Cross NM; RSNA COVID-19 task force. Special report of the RSNA COVID-19 task force: systematic review of outcomes associated with COVID-19 neuroimaging findings in hospitalized patients. Br J Radiol. 2021;94(1127):20210149. doi: 10.1259/bjr.20210149. Epub 2021 Apr 29.	Review
165	Polles AG, Jacobs WS, Brazle C, Merlo LJ. The role of alcohol biomarkers in detecting a physician's COVID-19-related acute stress response: A case report. J Addict Med. 2022;16(1):e62-e65. doi: 10.1097/ADM.0000000000000865.	Case
166	Vedam-Mai V, Deisseroth K, Giordano J, Lazaro-Munoz G, Chiong W, Suthana N, Langevin JP, Gill J, Goodman W, Provenza NR, Halpern CH, Shivacharan RS, Cunningham TN, Sheth SA, Pouratian N, Scangos KW, Mayberg HS, Horn A, Johnson KA, Butson CR, Gilron R, de Hemptinne C, Wilt R, Yaroshinsky M, Little S, Starr P, Worrell G, Shirvalkar P, Chang E, Volkmann J, Muthuraman M, Groppa S, Kühn AA, Li L, Johnson M, Otto KJ, Raike R, Goetz S, Wu C, Silburn P, Cheeran B, Pathak YJ, Malekmohammadi M, Gunduz A, Wong JK, Cernera S, Hu W, Wagle Shukla A, Ramirez-Zamora A, Deeb W, Patterson A, Foote KD, Okun MS. Proceedings of the Eighth Annual Deep Brain Stimulation Think Tank: Advances in Optogenetics, Ethical Issues Affecting DBS Research, Neuromodulatory Approaches for Depression, Adaptive Neurostimulation, and Emerging DBS Technologies. Front Hum Neurosci. 2021;15:644593. doi: 10.3389/fnhum.2021.644593. eCollection 2021 Apr 19.	No COVID-19
167	Chammas A, Bund C, Lersy F, Brisset JC, Ardellier FD, Kremer S, Namer IJ. Collicular hyperactivation in patients with COVID-19: A new finding on brain MRI and PET/CT. AJNR Am J Neuroradiol. 2021;42(8):1410-1414. doi: 10.3174/ajnr.A7158. Epub 2021 May 20.	Unfocused
168	Sun W, Chen WT, Zhang Q, Ma S, Huang F, Zhang L, Lu H. Post-traumatic growth experiences among COVID-19 confirmed cases in China: A qualitative study. Clin Nurs Res. 2021;30(7):1079-1087. doi: 10.1177/10547738211016951. Epub 2021 May 21.	Unrelated
169	Azoulay E, Pochard F, Reignier J, Argaud L, Bruneel F, Courbon P, Cariou A, Klouche K, Labbé V, Barbier F, Guitton C, Demoule A, Kouatchet A, Guisset O, Jourdain M, Papazian L, Van Der Meersch G, Reuter D, Souppart V, Resche-Rigon M, Darmon M, Kentish-Barnes N; FAMIREA Study Group. Symptoms of mental health disorders in critical care physicians facing the second COVID-19 wave: A cross-sectional study. Chest. 2021;160(3):944-955. doi: 10.1016/j.chest.2021.05.023. Epub 2021 May 21.	Unrelated
170	Carmassi C, Bui E, Bertelloni CA, Dell'Oste V, Pedrinelli V, Corsi M, Baldanzi S, Cristaudo A, Dell'Osso L, Buselli R. Validation of the Italian version of the peritraumatic distress inventory: validity, reliability and factor analysis in a sample of healthcare workers. Eur J Psychotraumatol. 2021;12(1):1879552. doi: 10.1080/20008198.2021.1879552. Online 2021 Mar 11.	Unrelated
171	Raban D, Barhaghi K, Timpone V, Jones W, Sauer B, Pollard R, Callen A. COVID-19 associated intracranial vasculopathy-MRI vessel wall imaging as adjunct to emergent CT angiography-a case report. Emerg Radiol. 2021;28(5):887-890. doi: 10.1007/s10140-021-01948-9. Epub 2021 May 26.	Case
172	Franzoi IG, Granieri A, Sauta MD, Agnesone M, Gonella M, Cavallo R, Lochner P, Bragazzi NL, Naldi A. Anxiety, post-traumatic stress, and burnout in health professionals during the COVID-19 pandemic: Comparing mental health professionals and other healthcare workers. Healthcare (Basel). 2021 May 27;9(6):635. doi: 10.3390/healthcare9060635.	Unrelated
173	Guerrero JI, Barragán LA, Martínez JD, Montoya JP, Peña A, Sobrino FE, Tovar-Spinoza Z, Ghotme KA. Central and peripheral nervous system involvement by COVID-19: a systematic review of the pathophysiology, clinical manifestations, neuropathology, neuroimaging, electrophysiology, and cerebrospinal fluid findings. BMC Infect Dis. 2021;21(1):515. doi: 10.1186/s12879-021-06185-6.	Review
174	Chadli A, Haraj NE, El Aziz S, Laidi S, Mounir A, Bensbaa S, Mjabber A, Barrou L, El Kettani El Hamidi C, Nsiri A, Al Harrar R, Ezzouine H, Charra B, Kamal N, Soussi Abdallaoui M, Bennouna GM, Marhoum El Filali K, Mchichi Alami K, Agoub M, El Mdaghri N, Ramdani B, Benghanem Gharbi M, Afif MH. COVID-19: Patient care after discharge from the Intensive Care Unit. Int J Clin Pract. 2021 Sep;75(9):e14270. doi: 10.1111/ijcp.14270. Epub 2021 Jul 5.	Unrelated
175	Toniolo S, Scarioni M, Di Lorenzo F, Hort J, Georges J, Tomic S, Nobili F, Frederiksen KS; Management Group of the EAN Dementia and Cognitive Disorders Scientific Panel. Dementia and COVID-19, a bidirectional liaison: risk factors, biomarkers, and optimal health care. J Alzheimers Dis. 2021;82(3):883-898. doi: 10.3233/JAD-210335.	Review
176	Waters L, Allen KA, Arslan G. Stress-related growth in adolescents returning to school after COVID-19 school closure. Front Psychol. 2021;12:643443. doi: 10.3389/fpsyg.2021.643443. eCollection 2021 May 20.	Unrelated
177	Favreau M, Hillert A, Osen B, Gärtner T, Hunatschek S, Riese M, Hewera K, Voderholzer U. Psychological consequences and differential impact of the COVID-19 pandemic in patients with mental disorders. Psychiatry Res. 2021;302:114045. doi: 10.1016/j.psychres.2021.114045. Epub 2021 Jun 6.	Unrelated
178	Manca R, De Marco M, Ince PG, Venneri A. Heterogeneity in regional damage detected by neuroimaging and neuropathological studies in older adults with COVID-19: A cognitive-neuroscience systematic review to inform the long-term impact of the virus on neurocognitive trajectories. Front Aging Neurosci. 2021;13:646908. doi: 10.3389/fnagi.2021.646908. eCollection 2021 Jun 3.	Review
179	Greenway MRF, Erben Y, Huang JF, Siegel JL, Lamb CJ, Badi MK, Sakusic A, Gopal N, Meschia JF, Lin MP. Yield of head imaging in ambulatory and hospitalized patients with SARS-CoV-2: A multi-center study of 8675 patients. Neurohospitalist. 2021;11(3):221-228. doi: 10.1177/1941874420980622. Epub 2020 Dec 16.	Unrelated
180	Kelsch RD, Silbergleit R, Krishnan A. Neuroimaging in the first 6 weeks of the COVID-19 pandemic in an 8-hospital campus: Observations and patterns in the brain, head and neck, and spine. J Comput Assist Tomogr. 2021;45(4):592-599. doi: 10.1097/RCT.0000000000001179.	Unfocused
181	Miragall M, Herrero R, Vara MD, Galiana L, Baños RM. The impact of strict and forced confinement due to the COVID-19 pandemic on positive functioning variables, emotional distress, and posttraumatic growth in a Spanish sample. Eur J Psychotraumatol. 2021 J;12(1):1918900. doi: 10.1080/20008198.2021.1918900.	Unrelated
182	Chen L, Lin D, Feng H. An Investigation of mental health status among medical staff following COVID-19 outbreaks: A cross-sectional study. Med Sci Monit. 2021;27:e929454. doi: 10.12659/MSM.929454.	Unrelated

183	Najt P, Richards HL, Fortune DG. Brain imaging in patients with COVID-19: A systematic review. Brain Behav Immun Health. 2021:100290. doi: 10.1016/j.bbih.2021.100290. Epub 2021 Jul 2.	Review
184	Ayandele O, Ramos-Vera CA, Iorfa SK, Chovwen CO, Olapegba PO. Exploring the complex pathways between the fear of COVID-19 and preventive health behavior among Nigerians: Mediation and moderation analyses. Am J Trop Med Hyg. 2021;105(3):701-707. doi: 10.4269/ajtmh.20-0994.	Unrelated
185	Kira IA, Shuwiekh HAM, Ashby JS, Elwakeel SA, Alhuwailah A, Sous MSF, Baali SBA, Azdaou C, Oliemat EM, Jamil HJ. The impact of COVID-19 traumatic stressors on mental health: Is COVID-19 a new trauma type? Int J Ment Health Addict. 2023;21(1):51-70. doi: 10.1007/s11469-021-00577-0. Epub 2021 Jul 6.	Unrelated
186	Samkaria A, Punjabi K, Sharma S, Joon S, Sandal K, Dasgupta T, Sharma P, Mandal PK. Brain stress mapping in COVID-19 survivors using MR spectroscopy: New avenue of mental health status monitoring. J Alzheimers Dis. 2021;83(2):523-530. doi: 10.3233/JAD-210287.	Opinion
187	Bafunno D, Romito F, Lagattolla F, Delvino VA, Minoia C, Loseto G, Dellino M, Guarini A, Catino A, Montrone M, Longo V, Pizzutilo P, Galetta D, Giotta F, Latorre AC, Russo A, Lorusso V, Cormio C. Psychological well-being in cancer outpatients during COVID-19. J BUON. 2021;26(3):1127-1134.	Unrelated
188	Kim PH, Kim M, Suh CH, Chung SR, Park JE, Kim SC, Choi YJ, Lee JH, Kim HS, Baek JH, Choi CG, Kim SJ. Neuroimaging findings in patients with COVID-19: A systematic review and meta-analysis. Korean J Radiol. 2021;22(11):1875-1885. doi: 10.3348/kjr.2021.0127. Epub 2021 Jul 1.	Review
189	Tu Y, Zhang Y, Li Y, Zhao Q, Bi Y, Lu X, Kong Y, Wang L, Lu Z, Hu L. Post-traumatic stress symptoms in COVID-19 survivors: a self-report and brain imaging follow-up study. Mol Psychiatry. 2021;26(12):7475-7480. doi: 10.1038/s41380-021-01223-w. Epub 2021 Jul 20.	**Included**
190	Casali N, Feraco T, Meneghetti C. Character strengths sustain mental health and post-traumatic growth during the COVID-19 pandemic. A longitudinal analysis. Psychol Health. 2022;37(12):1663-1679. doi: 10.1080/08870446.2021.1952587. Epub 2021 Jul 21.	Unrelated
191	Feng P, Chen Z, Becker B, Liu X, Zhou F, He Q, Qiu J, Lei X, Chen H, Feng T. Predisposing variations in fear-related brain networks prospectively predict fearful feelings during the 2019 Coronavirus (COVID-19) pandemic. Cereb Cortex. 2022;32(3):540-553. doi: 10.1093/cercor/bhab232.	Unsuitable
192	Looking after each other in a crisis-Lessons from Novichok and the parallels with Covid-19. Jenkins KSM, Thomas J, Duggan M, Scott H, Lang J. Nurs Crit Care. 2023 Jan;28(1):30-35. doi: 10.1111/nicc.12677. Epub 2021 Jul 28.	Unrelated
193	Samkaria A, Mandal PK. Brain Imaging in COVID-19. ACS Chem Neurosci. 2021;12(16):2953-2955. doi: 10.1021/acschemneuro.1c00467. Epub 2021 Aug 3.	Opinion
194	Liu D, Epskamp S, Isvoranu AM, Chen C, Liu W, Hong X. Network analysis of physical and psychiatric symptoms of hospital discharged patients infected with COVID-19. J Affect Disord. 2021;294:707-713. doi: 10.1016/j.jad.2021.07.043. Epub 2021 Jul 16.	Unrelated
195	Engelbrecht MC, Heunis JC, Kigozi NG. Post-traumatic stress and coping strategies of South African nurses during the second wave of the COVID-19 pandemic. Int J Environ Res Public Health. 2021;18(15):7919. doi: 10.3390/ijerph18157919.	Unrelated
196	Sanghvi D, Kale H. Imaging of COVID-19-associated craniofacial mucormycosis: a black and white review of the "black fungus". Clin Radiol. 2021;76(11):812-819. doi: 10.1016/j.crad.2021.07.004. Epub 2021 Jul 28.	Review.
197	Morales Chainé S, López Montoya A, Bosch Maldonado A, Beristain Aguirre A, Robles García R, Garibay Rubio CR, Astudillo García CI, Lira Chávez IA, Rangel Gómez MG. Mental health symptoms, binge drinking, and the experience of abuse during the COVID-19 lockdown in Mexico. Front Public Health. 2021;9:656036. doi: 10.3389/fpubh.2021.656036. eCollection 2021 Jul 22.	Unrelated
198	Fu Z, Tu Y, Calhoun VD, Zhang Y, Zhao Q, Chen J, Meng Q, Lu Z, Hu L. Dynamic functional network connectivity associated with post-traumatic stress symptoms in COVID-19 survivors. Neurobiol Stress. 2021;15:100377. doi: 10.1016/j.ynstr.2021.100377. Epub 2021 Aug 5.	FC
199	Pang L, Zhu S, Ma J, Zhu L, Liu Y, Ou G, Li R, Wang Y, Liang Y, Jin X, Du L, Jin Y. Intranasal temperature-sensitive hydrogels of cannabidiol inclusion complex for the treatment of post-traumatic stress disorder. Acta Pharm Sin B. 2021;11(7):2031-2047. doi: 10.1016/j.apsb.2021.01.014. Epub 2021 Jan 24.	Unrelated
200	D'Addario M, Zanatta F, Adorni R, Greco A, Fattirolli F, Franzelli C, Giannattasio C, Steca P. Depression symptoms as longitudinal predictors of the psychological impact of COVID-19 pandemic in hypertensive patients. Sci Rep. 2021;11(1):16496. doi: 10.1038/s41598-021-96165-2.	Unrelated
201	Rajagopalan V, Deoni S, Panigrahy A, Thomason ME. Is fetal MRI ready for neuroimaging prime time? An examination of progress and remaining areas for development. Dev Cogn Neurosci. 2021;51:100999. doi: 10.1016/j.dcn.2021.100999. Epub 2021 Aug 4.	Opinion
202	Morand A, Campion JY, Lepine A, Bosdure E, Luciani L, Cammilleri S, Chabrol B, Guedj E. Similar patterns of [18F]-FDG brain PET hypometabolism in paediatric and adult patients with long COVID: a paediatric case series. Eur J Nucl Med Mol Imaging. 2022;49(3):913-920. doi: 10.1007/s00259-021-05528-4. Epub 2021 Aug 19.	Case
203	Bahadirli S, Sagaltici E. Post-traumatic stress disorder in healthcare workers of emergency departments during the pandemic: A cross-sectional study. Am J Emerg Med. 2021;50:251-255. doi: 10.1016/j.ajem.2021.08.027. Epub 2021 Aug 14.	Unrelated
204	Islam MS, Rahman ME, Banik R, Emran MGI, Saiara N, Hossain S, Hasan MT, Sikder MT, Smith L, Potenza MN. Financial and mental health concerns of impoverished urban-dwelling Bangladeshi people during COVID-19. Front Psychol. 2021;12:663687. doi: 10.3389/fpsyg.2021.663687. eCollection 2021 Aug 6.	Unrelated
205	Zhao Q, Sun X, Xie F, Chen B, Wang L, Hu L, Dai Q. Impact of COVID-19 on psychological wellbeing. Int J Clin Health Psychol. 2021;21(3):100252. doi: 10.1016/j.ijchp.2021.100252. Epub 2021 May 27.	Unrelated
206	Yang X, Yip BHK, Lee EKP, Zhang D, Wong SYS. The relationship between technology use and problem technology use and potential psychosocial mechanisms: Population-based telephone survey in community adults during COVID-19. Front Psychol. 2021;12:696271. doi: 10.3389/fpsyg.2021.696271. eCollection 2021 Aug 9.	Unrelated
207	Kavak S, Yildirim MS, Altındag R, Mertsoy Y, Alakus MF, Guleken MD, Kaya S. Correlation of neuroimaging findings with clinical presentation and laboratory data in patients with COVID-19: A single-center study. Biomed Res Int. 2021;2021:2013371. doi: 10.1155/2021/2013371. eCollection 2021 Aug 10.	Unsuitable
208	Ho RC, Sharma VK, Tan BYQ, Ng AYY, Lui YS, Husain SF, Ho CS, Tran BX, Pham QH, McIntyre RS, Chan ACY. Comparison of brain activation patterns during olfactory stimuli between recovered COVID-19 patients and healthy controls: A functional near-infrared spectroscopy (fNIRS) study. Brain Sci. 2021;11(8):968. doi: 10.3390/brainsci11080968.	Unsuitable
209	Picano E, Ciampi Q, Cortigiani L, Arruda-Olson AM, Borguezan-Daros C, de Castro E Silva Pretto JL, Cocchia R, Bossone E, Merli E, Kane GC, Varga A, Agoston G, Scali MC, Morrone D, Simova I, Samardjieva M, Boshchenko A, Ryabova T, Vrublevsky A, Palinkas A, Palinkas ED, Sepp R, Torres MAR, Villarraga HR, Preradović TK, Citro R, Amor M, Mosto H, Salamè M, Leeson P, Mangia C, Gaibazzi N, Tuttolomondo D, Prota C, Peteiro J, Van De Heyning CM, D'Andrea A, Rigo F, Nikolic A, Ostojic M, Lowenstein J, Arbucci R, Haber DML, Merlo PM, Wierzbowska-Drabik K, Kasprzak JD, Haberka M, Camarozano AC, Ratanasit N, Mori F, D'Alfonso MG, Tassetti L, Milazzo A, Olivotto I, Marchi A, Rodriguez-Zanella H, Zagatina A, Padang R, Dekleva M, Djordievic-Dikic A, Boskovic N, Tesic M, Giga V, Beleslin B, Di Salvo G, Lorenzoni V, Cameli M, Mandoli GE, Bombardini T, Caso P, Celutkiene J, Barbieri A, Benfari G, Bartolacelli Y, Malagoli A, Bursi F, Mantovani F, Villari B, Russo A, De Nes M, Carpeggiani C, Monte I, Re F, Cotrim C, Bilardo G, Saad AK, Karuzas A, Matuliauskas D, Colonna P, Antonini-Canterin F, Pepi M, Pellikka PA, The Stress Echo Study Group Of The Italian	Protocol

	Society Of Echocardiography And Cardiovascular Imaging Siecvi. Stress Echo 2030: The Novel ABCDE-(FGLPR) Protocol to define the future of imaging. J Clin Med. 2021;10(16):3641. doi: 10.3390/jcm10163641.	
210	Nearchou F, Douglas E. Traumatic distress of COVID-19 and depression in the general population: Exploring the role of resilience, anxiety, and hope. Int J Environ Res Public Health. 2021;18(16):8485. doi: 10.3390/ijerph18168485.	Unrelated
211	Ullah I, Prévost J, Ladinsky MS, Stone H, Lu M, Anand SP, Beaudoin-Bussières G, Symmes K, Benlarbi M, Ding S, Gasser R, Fink C, Chen Y, Tauzin A, Goyette G, Bourassa C, Medjahed H, Mack M, Chung K, Wilen CB, Dekaban GA, Dikeakos JD, Bruce EA, Kaufmann DE, Stamatatos L, McGuire AT, Richard J, Pazgier M, Bjorkman PJ, Mothes W, Finzi A, Kumar P, Uchil PD. Live imaging of SARS-CoV-2 infection in mice reveals that neutralizing antibodies require Fc function for optimal efficacy. Immunity. 2021;54(9):2143-2158.e15. doi: 10.1016/j.immuni.2021.08.015. Epub 2021 Aug 18.	Animal
212	Günbey HP, Rona G, Adıgüzel Karaoysal Ö, Batırel A, Özen Barut B. Correlation of neuroimaging and thorax CT findings in patients with COVID-19: A large single-center experience. Turk J Med Sci. 2021;51(6):2850-2860. doi: 10.3906/sag-2105-138.	Unsuitable
213	Kulason S, Ratnanather JT, Miller MI, Kamath V, Hua J, Yang K, Ma M, Ishizuka K, Sawa A. A comparative neuroimaging perspective of olfaction and higher-order olfactory processing: on health and disease. Semin Cell Dev Biol. 2022;129:22-30. doi: 10.1016/j.semcdb.2021.08.009. Epub 2021 Aug 27.	Review
214	Liu JJW, Nazarov A, Plouffe RA, Forchuk CA, Deda E, Gargala D, Le T, Bourret-Gheysen J, Soares V, Nouri MS, Hosseiny F, Smith P, Roth M, MacDougall AG, Marlborough M, Jetly R, Heber A, Albuquerque J, Lanius R, Balderson K, Dupuis G, Mehta V, Richardson JD. Exploring the well-being of health care workers during the COVID-19 pandemic: Protocol for a prospective longitudinal study. JMIR Res Protoc. 2021;10(9):e32663. doi: 10.2196/32663.	Protocol
215	Alexander C, Vasefi M. Cannabidiol and the corticoraphe circuit in post-traumatic stress disorder. IBRO Neurosci Rep. 2021;11:88-102. doi: 10.1016/j.ibneur.2021.08.001. eCollection 2021 Dec; Aug 21.	Review
216	Srifuengfung M, Thana-Udom K, Ratta-Apha W, Chulakadabba S, Sanguanpanich N, Viravan N. Impact of the COVID-19 pandemic on older adults living in long-term care centers in Thailand, and risk factors for post-traumatic stress, depression, and anxiety. J Affect Disord. 2021;295:353-365. doi: 10.1016/j.jad.2021.08.044. Epub 2021 Aug 28.	Unrelated
217	Edison P. *Brain connectivity:* Advances in neuroimaging to investigate COVID-19. Brain Connect. 2021;11(7):502-504. doi: 10.1089/brain.2021.29026.ped.	Opinion
218	Khandwala K, Mubarak F, Ahmad MN. Imaging patterns of encephalopathy in patients with COVID-19. J Coll Physicians Surg Pak. 2021;31(1):S42-S45. doi: 10.29271/jcpsp.2021.Supp1.S42.	Unsuitable
219	Villarreal-Zegarra D, Copez-Lonzoy A, Vilela-Estrada AL, Huarcaya-Victoria J. Depression, post-traumatic stress, anxiety, and fear of COVID-19 in the general population and health-care workers: prevalence, relationship, and explicative model in Peru. BMC Psychiatry. 2021;21(1):455. doi: 10.1186/s12888-021-03456-z.	No neuroim.
220	Kalekar T, Thakker V, Bansal A. Role of neuroimaging in COVID 19 infection-A retrospective study. J Radiol Nurs. 2021;40(4):370-376. doi: 10.1016/j.jradnu.2021.09.003. Epub 2021 Sep 17.	Unfocused
221	Spurio MG. Mourning from Covid-19 and Post Traumatic Stress Disorder. New therapeutic tools in the treatment of pathological bereavement. Psychiatr Danub. 2021;33(Suppl 9):102-107.	Unrelated
222	Norred MA, Haselden LC, Sahlem GL, Wilkerson AK, Short EB, McTeague LM, George MS. TMS and CBT-I for comorbid depression and insomnia. Exploring feasibility and tolerability of transcranial magnetic stimulation (TMS) and cognitive behavioral therapy for insomnia (CBT-I) for comorbid major depressive disorder and insomnia during the COVID-19 pandemic. Brain Stimul. 2021;14(6):1508-1510. doi: 10.1016/j.brs.2021.09.007. Epub 2021 Sep 23.	Unrelated
223	Mazzacane F, Zito A, Magno S, Persico A, Mazzoleni V, Asteggiano C, Rognone E, Pichiecchio A, Padovani A, Cavallini A, Morotti A. Vessel wall magnetic resonance imaging in COVID-19-associated cryptogenic ischemic stroke. Eur J Neurol. 2022;29(2):615-619. doi: 10.1111/ene.15128. Epub 2021 Oct 6.	Unfocused
224	Ghio L, Patti S, Piccinini G, Modafferi C, Lusetti E, Mazzella M, Del Sette M. Anxiety, depression and risk of post-traumatic stress disorder in health workers: The relationship with burnout during COVID-19 pandemic in Italy. Int J Environ Res Public Health. 2021;18(18):9929. doi: 10.3390/ijerph18189929.	Unrelated
225	Hajac M, Olchowy C, Poręba R, Gać P. COVID-19 pathology imaging: A one-year perspective. Dent Med Probl. 2021;58(3):377-384. doi: 10.17219/dmp/135814.	Review
226	Peng X, Yang Y, Gao P, Ren Y, Hu D, He Q. Negative and positive psychological experience of frontline nurses in combatting COVID-19: A qualitative study. J Nurs Manag. 2022;30(7):2185-2193. doi: 10.1111/jonm.13481. Epub 2021 Nov 1.	Unrelated
227	Grande M, Lattanzio L, Buard I, McKendrick AM, Chan YM, Pelak VS. A study protocol for an open-label feasibility treatment trial of visual snow syndrome with transcranial magnetic stimulation. Front Neurol. 2021;12:724081. doi: 10.3389/fneur.2021.724081. eCollection 2021 Sep 24.	Protocol
228	Tizenberg BN, Brenner LA, Lowry CA, Okusaga OO, Benavides DR, Hoisington AJ, Benros ME, Stiller JW, Kessler RC, Postolache TT. Biological and psychological factors determining neuropsychiatric outcomes in COVID-19. Curr Psychiatry Rep. 2021;23(10):68. doi: 10.1007/s11920-021-01275-3.	Review
229	Gross A, Albrecht T. One year of COVID-19 pandemic: what we Radiologists have learned about imaging. Rofo. 2022;194(2):141-151. doi: 10.1055/a-1522-3155. Epub 2021 Oct 14.	Review
230	Lang M, Chang YS, Mehan WA Jr, Rincon SP, Buch K. Long-term neuroimaging follow-up of COVID-19-related leukoencephalopathy. Neuroradiology. 2021;63(12):2153-2156. doi: 10.1007/s00234-021-02829-1. Epub 2021 Oct 18.	Unrelated
231	Burma JS, Lapointe AP, Soroush A, Oni IK, Smirl JD, Dunn JF. Insufficient sampling frequencies skew heart rate variability estimates: Implications for extracting heart rate metrics from neuroimaging and physiological data. J Biomed Inform. 2021;123:103934. doi: 10.1016/j.jbi.2021.103934. Epub 2021 Oct 16.	Unrelated
232	Podury S, Srivastava S, Khan E, Kakara M, Tandon M, Shrestha AK, Freeland K, Wen S, Sriwastava S. Relevance of CSF, serum and neuroimaging markers in CNS and PNS manifestation in COVID-19: A systematic review of case report and case series. Brain Sci. 2021;11(10):1354. doi: 10.3390/brainsci11101354.	Review
233	Khaleghi M, Aziz-Ahari A, Rezaeian N, Asadian S, Mounesi Sohi A, Motamedi O, Azhdeh S. The valuable role of imaging modalities in the diagnosis of the uncommon presentations of COVID-19: An educative case series. Case Rep Med. 2021;2021:7213627. doi: 10.1155/2021/7213627. eCollection 2021 Oct 13.	Case
234	Chukhlovina ML, Chukhlovin AA. Особенности ведения пациентов с черепно-мозговой травмой [Features of the patient management with traumatic brain injury]. Zh Nevrol Psikhiatr Im S S Korsakova. 2021;121(9):145-151. Russian. doi: 10.17116/jnevro2021121091145.	Unrelated
235	Kao WT, Hsu ST, Chou FH, Chou LS, Hsieh KY, Li DJ, Lin GG, Wu PJ, Chen WJ, Huang JJ. The societal influences and quality of life among healthcare team members during the COVID-19 pandemic. Front Psychiatry. 2021;12:706443. doi: 10.3389/fpsyt.2021.706443. eCollection 2021 Oct 11.	Unrelated
236	Guggenberger KV, Bley TA, Vogt ML, Urbach H, Meckel S. High-resolution black blood vessel wall imaging in COVID-19 encephalopathy-Is it really endotheliitis? Clin Neuroradiol. 2022;32(1):295-296. doi: 10.1007/s00062-021-01109-y. Epub 2021 Oct 28.	Unrelated
237	Wild J, McKinnon A, Wilkins A, Browne H. Post-traumatic stress disorder and major depression among frontline healthcare staff working during the COVID-19 pandemic. Br J Clin Psychol. 2022;61(3):859-866. doi: 10.1111/bjc.12340. Epub 2021 Oct 29.	Unrelated

238	Masiello E, Buonsenso D, Lazzareschi I, Gatto A, Piastra M, Chiaretti A, Valentini P. Case report: Kawasaki shock syndrome with polycyclic eruption: A peculiar brain imaging. Front Pediatr. 2021;9:651457. doi: 10.3389/fped.2021.651457. eCollection 2021 Oct 15.	Case
239	Walsh CL, Tafforeau P, Wagner WL, Jafree DJ, Bellier A, Werlein C, Kühnel MP, Boller E, Walker-Samuel S, Robertus JL, Long DA, Jacob J, Marussi S, Brown E, Holroyd N, Jonigk DD, Ackermann M, Lee PD. Imaging intact human organs with local resolution of cellular structures using hierarchical phase-contrast tomography. Nat Methods. 2021;18(12):1532-1541. doi: 10.1038/s41592-021-01317-x. Epub 2021 Nov 4.	No COVID-19
240	Hughes NM, Hammer MM, Awad MM, Jacene HA. Radiation recall pneumonitis on FDG PET/CT triggered by COVID-19 vaccination. Clin Nucl Med. 2022;47(3):e281-e283. doi: 10.1097/RLU.0000000000003980.	Case
241	Benedetti F, Palladini M, Paolini M, Melloni E, Vai B, De Lorenzo R, Furlan R, Rovere-Querini P, Falini A, Mazza MG.Brain correlates of depression, post-traumatic distress, and inflammatory biomarkers in COVID-19 survivors: A multimodal magnetic resonance imaging study. Brain Behav Immun Health. 2021;18:100387. doi: 10.1016/j.bbih.2021.100387. Epub 2021 Nov 2.	**Included**
242	Uginet M, Breville G, Hofmeister J, Machi P, Lalive PH, Rosi A, Fitsiori A, Vargas MI, Assal F, Allali G, Lövblad KO. Answer to Letter to the Editor: High-resolution Black Blood Vessel Wall Imaging in COVID-19 Encephalopathy-is it Really Endotheliitis? Clin Neuroradiol. 2022;32(1):297-298. doi: 10.1007/s00062-021-01113-2. Epub 2021 Nov 10.	Opinion
243	Singh V, Allawadhi P, Khurana A, Banothu AK, Bharani KK. Critical neurological features of COVID-19: Role of imaging methods and biosensors for effective diagnosis. Sens Int. 2021;2:100098. doi: 10.1016/j.sintl.2021.100098. Epub 2021 May 13.	Review
244	Park YS, Park KH, Lee J. Validation of the Korean Version of Impact of Event Scale-Revised (IES-R) in Korean Nurses during the COVID-19 Pandemic. Int J Environ Res Public Health. 2021;18(21):11311. doi: 10.3390/ijerph182111311.	Unrelated
245	Sfera A, Osorio C, Rahman L, Zapata-Martín Del Campo CM, Maldonado JC, Jafri N, Cummings MA, Maurer S, Kozlakidis Z. PTSD as an Endothelial Disease: Insights From COVID-19. Front Cell Neurosci. 2021;15:770387. doi: 10.3389/fncel.2021.770387. eCollection 2021 Oct 29.	Review.
246	Griffanti L, Raman B, Alfaro-Almagro F, Filippini N, Cassar MP, Sheerin F, Okell TW, Kennedy McConnell FA, Chappell MA, Wang C, Arthofer C, Lange FJ, Andersson J, Mackay CE, Tunnicliffe EM, Rowland M, Neubauer S, Miller KL, Jezzard P, Smith SM. Adapting the UK Biobank Brain Imaging Protocol and Analysis Pipeline for the C-MORE Multi-Organ Study of COVID-19 Survivors. Front Neurol. 2021 Oct 29;12:753284. doi: 10.3389/fneur.2021.753284. eCollection 2021. PMID: 34777224 .	Unrelated
247	Plouffe RA, Nazarov A, Forchuk CA, Gargala D, Deda E, Le T, Bourret-Gheysen J, Jackson B, Soares V, Hosseiny F, Smith P, Roth M, MacDougall AG, Marlborough M, Jetly R, Heber A, Albuquerque J, Lanius R, Balderson K, Dupuis G, Mehta V, Richardson JD. Impacts of morally distressing experiences on the mental health of Canadian health care workers during the COVID-19 pandemic. Eur J Psychotraumatol. 2021;12(1):1984667. doi: 10.1080/20008198.2021.1984667. eCollection 2021 Nov 10.	Unrelated
248	Marsiglia M, Chwalisz BK, Maher M. Neuroradiologic Imaging of Neurologic and Neuro-Ophthalmic Complications of Coronavirus-19 Infection. J Neuroophthalmol. 2021;41(4):452-460. doi: 10.1097/WNO.0000000000001454.	Review
249	Nagarajan R, Krishnamoorthy Y, Basavarachar V, Dakshinamoorthy R.Prevalence of post-traumatic stress disorder among survivors of severe COVID-19 infections: A systematic review and meta-analysis.J Affect Disord. 2022 Feb 15;299:52-59. doi: 10.1016/j.jad.2021.11.040. Epub 2021 Nov 17.	Review
250	Yadav T, Tiwari S, Gupta A, Garg PK, Khera PS, Rajagopal R, Goyal A, Soni K, Chugh A, Jain V, Sureka B, Elhence P, Misra S. Magnetic Resonance Imaging in Coronavirus Disease - 2019 Associated Rhino-Orbital-Cerebral Mucormycosis (CA-ROCM) - Imaging Analysis of 50 Consecutive Patients. Curr Probl Diagn Radiol. 2022;51(1):112-120. doi: 10.1067/j.cpradiol.2021.09.004. Epub 2021 Nov 3.	Review
251	Gilsbach S, Herpertz-Dahlmann B, Konrad K. Psychological Impact of the COVID-19 Pandemic on Children and Adolescents With and Without Mental Disorders. Front Public Health. 2021;9:679041. doi: 10.3389/fpubh.2021.679041. eCollection 2021 Nov 5.	Unrelated
252	Bellan M, Baricich A, Patrucco F, Zeppegno P, Gramaglia C, Balbo PE, Carriero A, Amico CS, Avanzi GC, Barini M, Battaglia M, Bor S, Cantaluppi V, Cappellano G, Ceruti F, Chiocchetti A, Clivati E, Giordano M, Cuneo D, Gambaro E, Gattoni E, Loro A, Manfredi M, Morosini U, Murano F, Paracchini E, Patti G, Pinato DJ, Raineri D, Rolla R, Sainaghi PP, Tricca S, Pirisi M. Long-term sequelae are highly prevalent one year after hospitalization for severe COVID-19. Sci Rep. 2021;11(1):22666. doi: 10.1038/s41598-021-01215-4.	Unrelated
253	Chen W, Gao R, Yang T. Factor Structure and Psychometric Properties for the PTSD Checklist of Chinese Adolescents in the Closed Period after the COVID-19 Outbreak. Int J Environ Res Public Health. 2021;18(22):12245. doi: 10.3390/ijerph182212245.	Unrelated
254	Rudroff T, Workman CD, Ponto LLB.18F-FDG-PET Imaging for Post-COVID-19 Brain and Skeletal Muscle Alterations.Viruses. 2021;13(11):2283. doi: 10.3390/v13112283.	Review
255	Riello M, Purgato M, Bove C, Tedeschi F, MacTaggart D, Barbui C, Rusconi E. Effectiveness of self-help plus (SH+) in reducing anxiety and post-traumatic symptomatology among care home workers during the COVID-19 pandemic: a randomized controlled trial. R Soc Open Sci. 2021;8(11):210219. doi: 10.1098/rsos.210219. eCollection 2021 Nov 24.	Unrelated
256	Zhang X, Liu J, Yang Y, Zhao S, Guo L, Han J, Hu X. Test-retest reliability of dynamic functional connectivity in naturalistic paradigm functional magnetic resonance imaging. Hum Brain Mapp. 2022;43(4):1463-1476. doi: 10.1002/hbm.25736. Epub 2021 Dec 6.	No COVID-19
257	Helminen EC, Scheer JR, Jackson SD, Brisbin CD, Batchelder AW, Cascalheira CJ, Sullivan TP. PTSD Symptoms and Hazardous Drinking Indicators among Trauma-Exposed Sexual Minority Women during Heightened Societal Stress. Behav Med. 2021:1-12. doi: 10.1080/08964289.2021.2006132. Online ahead of print 2021 Dec 6.	No COVID-19
258	Chi XL, Huang QM, Liu XF, Huang LY, Hu MJ, Chen ZJ, Jiao C, Stubbs B, Hossain MM, Zou LY.Self-compassion and resilience mediate the relationship between childhood exposure to domestic violence and posttraumatic growth/stress disorder during COVID-19 pandemic. World J Psychiatry. 2021 Nov 19;11(11):1106-1115. doi: 10.5498/wjp.v11.i11.1106. eCollection 2021 Nov 19.	Unrelated
259	Genis-Mendoza AD, Martínez-Magaña JJ, López-Narváez ML, González-Castro TB, Juárez-Rojop IE, Nicolini H, Tovilla-Zárate CA, Castillo-Avila RG. Mental Health Problems Due to Social Isolation During the COVID-19 Pandemic in a Mexican Population. Front Public Health. 2021;9:703450. doi: 10.3389/fpubh.2021.703450. eCollection 2021 Nov 23.	Unrelated
260	Hang Y, Boryczka J, Wu N. Visible-light and near-infrared fluorescence and surface-enhanced Raman scattering point-of-care sensing and bio-imaging: a review. Chem Soc Rev. 2022;51(1):329-375. doi: 10.1039/c9cs00621d.	Review.
261	Du YY, Zhao W, Zhou XL, Zeng M, Yang DH, Xie XZ, Huang SH, Jiang YJ, Yang WH, Guo H, Sun H, Liu JY, Liu P, Zhou ZG, Luo H, Liu J. Survivors of COVID-19 exhibit altered amplitudes of low frequency fluctuation in the brain: a resting-state functional magnetic resonance imaging study at 1-year follow-up. Neural Regen Res. 2022;17(7):1576-1581. doi: 10.4103/1673-5374.327361.	fMRI
262	Nisticò V, Gambini O, Pizzi L, Faggioli R, Priori A, Demartini B. A paradoxical psychological impact of COVID-19 among a sample of Italian adults with High Functioning Autism Spectrum Disorder. J Clin Neurosci. 2022;95:27-30. doi: 10.1016/j.jocn.2021.11.026. Epub 2021 Nov 29.	Unrelated
263	Andreassi S, Monaco S, Salvatore S, Sciabica GM, De Felice G, Petrovska E, Mariani R. To Work or Not to Work, That Is the Question: The Psychological Impact of the First COVID-19 Lockdown on the Elderly, Healthcare Workers, and Virtual Workers. Healthcare (Basel). 2021;9(12):1754. doi: 10.3390/healthcare9121754.	Unrelated
264	Schmitt N, Mattern E, Cignacco E, Seliger G, König-Bachmann M, Striebich S, Ayerle GM.Effects of the Covid-19 pandemic on maternity staff in 2020 - a scoping review. BMC Health Serv Res. 2021;21(1):1364. doi: 10.1186/s12913-021-07377-1.	Review
265	Yu AT, Absar NM. Long-term Neuropsychiatric Complications and 18F-FDG-PET Hypometabolism in the Brain From Prolonged Infection of COVID-19. Alzheimer Dis Assoc Disord. 2022;36(2):173-175. doi: 10.1097/WAD.0000000000000485. Epub 2021 Dec 30.	Unrelated

266	Enlow PT, Phan TT, Lewis AM, Hildenbrand AK, Sood E, Canter KS, Vega G, Alderfer MA, Kazak AE.Validation of the COVID-19 Exposure and Family Impact Scales. J Pediatr Psychol. 2022;47(3):259-269. doi: 10.1093/jpepsy/jsab136.	Unrelated
267	Jindal G, Sethi A, Bhargarva K, Sethi S, Mittal A, Singh U, Singh S, Shrivastava A. Imaging findings in invasive rhino-orbito-cerebral mucormycosis in post-COVID-19 patients. Proc (Bayl Univ Med Cent). 2021;35(1):32-34. doi: 10.1080/08998280.2021.1981100. eCollection 2022 Sep 30.	Unrelated
268	Rossi R, Socci V, Talevi D, Niolu C, Pacitti F, Di Marco A, Rossi A, Siracusano A, Di Lorenzo G, Olff M. Trauma-spectrum symptoms among the Italian general population in the time of the COVID-19 outbreak. Eur J Psychotraumatol. 2021;12(1):1855888. doi: 10.1080/20008198.2020.1855888. eCollection 2021 Jan 26.	Unrelated
269	Manu P.Post-COVID Fatigue: Functional Brain Imaging and Cognitive Testing.Am J Ther. 2021 Dec 1;29(1):e85-e86. doi: 10.1097/MJT.0000000000001459.PMID: 34994350 No abstract available.	No COVID-19
270	Metwally MI, Mobashir M, Sweed AH, Mahmoud SM, Hassan AG, ElKashishy K, Eesa M, Elnashar I, Elmalt A, Elsayed AI, Idris SK, Elshetry ASF.Post COVID-19 Head and Neck Mucormycosis: MR Imaging Spectrum and Staging. Acad Radiol. 2022;29(5):674-684. doi: 10.1016/j.acra.2021.12.007. Epub 2021 Dec 15.	Unfocused
271	Chun KJ, Zamora E, Coco M. Brain perfusion single-photon emission computerized tomography/computerized tomography: Stroke-like manifestations of COVID-19 with transient antiphospholipid elevation. World J Nucl Med. 2021;20(4):405-407. doi: 10.4103/wjnm.wjnm_28_21. eCollection 2021 Oct-Dec; Nov 25.	Unrelated
272	Siripongsatian D, Kunawudhi A, Promteangtrong C, Kiatkittikul P, Jantarato A, Choolam A, Ponglikitmongkol K, Siripongboonsitti T, Kaeowirun T, Chotipanich C. Alterations in ^{18}F-FDG PET/MRI and ^{15}O-water PET brain findings in patients with neurological symptoms after COVID-19 vaccination: A pilot study. Clin Nucl Med. 2022;47(3):e230-e239. doi: 10.1097/RLU.0000000000004041.	Unrelated
273	Garrido-Hernansaiz H, Rodríguez-Rey R, Collazo-Castiñeira P, Collado S.The posttraumatic growth inventory-short form (PTGI-SF): A psychometric study of the Spanish population during the COVID-19 pandemic. Curr Psychol. 2022:1-10. doi: 10.1007/s12144-021-02645-z. Online ahead of print 2022 Jan 11.	Unrelated
274	Pszczolkowski S, Cottam WJ, Briley PM, Iwabuchi SJ, Kaylor-Hughes C, Shalabi A, Babourina-Brooks B, Berrington A, Barber S, Suazo Di Paola A, Blamire A, McAllister-Williams RH, Parikh J, Abdelghani M, Matthäus L, Hauffe R, Liddle P, Auer DP, Morriss R.Connectivity-Guided Theta Burst Transcranial Magnetic Stimulation Versus Repetitive Transcranial Magnetic Stimulation for Treatment-Resistant Moderate to Severe Depression: Magnetic Resonance Imaging Protocol and SARS-CoV-2-Induced Changes for a Randomized Double-blind Controlled Trial.JMIR Res Protoc. 2022;11(1):e31925. doi: 10.2196/31925.	Protocol
275	Sklinda K, Górecki A, Dorobek M, Walecki J, Modrzyńska A, Mruk B. Ischaemic background of brain fog in long-haul COVID-19 - a nuclear magnetic resonance spectroscopy-based metabonomic analysis. Preliminary results. Pol J Radiol. 2021;86:e654-e660. doi: 10.5114/pjr.2021.111100. eCollection 2021 Dec 13.	Unrelated
276	Kiatkittikul P, Promteangtrong C, Kunawudhi A, Siripongsatian D, Siripongboonsitti T, Ruckpanich P, Thongdonpua S, Jantarato A, Piboonvorawong C, Fonghoi N, Chotipanich C. Abnormality pattern of F-18 FDG PET whole body with functional MRI brain in post-acute COVID-19. Nucl Med Mol Imaging. 2022;56(1):29-41. doi: 10.1007/s13139-021-00730-6. Epub 2022 Jan 15.	Unrelated
277	Chiam M, Ho CY, Quah E, Chua KZY, Ng CWH, Lim EG, Tan JRM, Wong RSM, Ong YT, Soong YL, Kwek JW, Yong WS, Loh KWJ, Lim C, Mason S, Krishna LKR.Changing self-concept in the time of COVID-19: a close look at physician reflections on social media.Philos Ethics Humanit Med. 2022;17(1):1. doi: 10.1186/s13010-021-00113-x.	Unrelated
278	Yang J, Menhas R, Dai J, Younas T, Anwar U, Iqbal W, Ahmed Laar R, Muddasar Saeed M. Virtual reality fitness (VRF) for behavior management during the COVID-19 pandemic: A mediation analysis approach. Psychol Res Behav Manag. 2022;15:171-182. doi: 10.2147/PRBM.S350666. eCollection 2022 Jan 20.	Unrelated
279	Hada M, Gupta P, Bagarhatta M, Tripathy K, Harsh A, Khilnani K, Mendiratta K, Agarwal S, Chouhan JK, Bhandari S. Orbital magnetic resonance imaging profile and clinicoradiological correlation in COVID-19-associated rhino-orbital-cerebral mucormycosis: A single-center study of 270 patients from North India. Indian J Ophthalmol. 2022;70(2):641-648. doi: 10.4103/ijo.IJO_1652_21.	Unfocused
280	Abe K, Tei S, Takahashi H, Fujino J. Structural brain correlates of burnout severity in medical professionals: A voxel-based morphometric study. Neurosci Lett. 2022;772:136484. doi: 10.1016/j.neulet.2022.136484. Epub 2022 Jan 30.	No COVID-19
281	Levi P, Moss J. Intensive care unit nurses' lived experiences of psychological stress and trauma caring for COVID-19 patients. Workplace Health Saf. 2022 Aug;70(8):358-367. doi: 10.1177/21650799211064262. Epub 2022 Feb 3.	Unrelated
282	Esteban-Sepúlveda S, Terradas-Robledo R, Castro-Ribeiro T, García-Pagès E, Sobregrau-Sangrà P, Lacueva-Pérez L. Pandemia COVID-19 sobre profesionales sanitarios en un hospital de tercer nivel en España: cambios laborales durante la primera ola, salud mental a los 4 meses y seguimiento a los 9 meses [COVID-19 pandemic on health professionals in a third level hospital in Spain: job changes during the first wave, mental health at 4 months, and follow-up at 9 months]. Enferm Clin. 2022;32(3):143-151. Spanish. doi: 10.1016/j.enfcli.2021.12.009. Epub 2022 Feb 2.	Unrelated
283	Shi J, Sznajder KK, Liu S, Xie X, Yang X, Zheng Z. Resilience and posttraumatic growth of patients with breast cancer during the COVID-19 pandemic in China: The mediating effect of recovery. Front Psychol. 2022;12:811078. doi: 10.3389/fpsyg.2021.811078. eCollection 2021 Jan 20.	Unrelated
284	Damiano RF, Caruso MJG, Cincoto AV, de Almeida Rocca CC, de Pádua Serafim A, Bacchi P, Guedes BF, Brunoni AR, Pan PM, Nitrini R, Beach S, Fricchione G, Busatto G, Miguel EC, Forlenza OV; HCFMUSP COVID-19 Study Group. Post-COVID-19 psychiatric and cognitive morbidity: Preliminary findings from a Brazilian cohort study. Gen Hosp Psychiatry. 2022;75:38-45. doi: 10.1016/j.genhosppsych.2022.01.002. Epub 2022 Jan 6.	Unrelated
285	Fidel Kinori SG, Carot-Sans G, Cuartero A, Valero-Bover D, Roma Monfa R, Garcia E, Pérez Sust P, Blanch J, Piera-Jiménez J, Ramos-Quiroga JA. A web-based app for emotional management during the COVID-19 pandemic: platform development and retrospective analysis of its use throughout two waves of the outbreak in Spain. JMIR Form Res. 2022 Mar 31;6(3):e27402. doi: 10.2196/27402.	Unrelated
286	Meyer PT, Hellwig S, Blazhenets G, Hosp JA. Molecular imaging findings on acute and long-term effects of COVID-19 on the brain: A systematic review. J Nucl Med. 2022;63(7):971-980. doi: 10.2967/jnumed.121.263085. Epub 2022 Feb 17.	Review
287	Sawalha J, Yousefnezhad M, Shah Z, Brown MRG, Greenshaw AJ, Greiner R. Detecting presence of PTSD using sentiment analysis from text data. Front Psychiatry. 2022;12:811392. doi: 10.3389/fpsyt.2021.811392. eCollection 2021 Feb 1.	No COVID-19
288	Bountress KE, Cusack SE, Conley AH, Aggen SH, The Spit For Science Working Group, Vassileva J, Dick DM, Amstadter AB. The COVID-19 pandemic impacts psychiatric outcomes and alcohol use among college students. Eur J Psychotraumatol. 2022 Feb 14;13(1):2022279. doi: 10.1080/20008198.2021.2022279. eCollection 2022.PMID: 35186215 .	Unrelated
289	Duarte AC, Fujiki RHM, Glória LFP, Fragoso DC, Pacheco FT, Calvi C, Rosa-Júnior M, Maia Junior ACM, Rocha AJD. Neurological imaging findings in hospitalized COVID-19 patients: a retrospective observational study in two Brazilian reference centers. Arq Neuropsiquiatr. 2022;80(5):490-496. doi: 10.1590/0004-282X-ANP-2021-0103.	**Included**
290	Chater AM, Howlett N, Shorter GW, Zakrzewski-Fruer JK, Williams J. Reflections on experiencing parental bereavement as a young person: A retrospective qualitative study. Int J Environ Res Public Health. 2022;19(4):2083. doi:10.3390/ijerph19042083.	No COVID-19
291	Smith AJ, Shoji K, Griffin BJ, Sippel LM, Dworkin ER, Wright HM, Morrow E, Locke A, Love TM, Harris JI, Kaniasty K, Langenecker SA, Benight CC. Social cognitive mechanisms in healthcare worker resilience across time during the pandemic. Soc Psychiatry Psychiatr Epidemiol. 2022;57(7):1457-1468. doi: 10.1007/s00127-022-02247-5. Epub 2022 Feb 26.	No COVID-19

292	Li W, Zhao N, Yan X, Xu X, Zou S, Wang H, Li Y, Du X, Zhang L, Zhang Q, Cheung T, Ungvari GS, Ng CH, Xiang YT. Network Analysis of depression, anxiety, posttraumatic stress symptoms, insomnia, pain, and fatigue in clinically stable older patients with psychiatric disorders during the COVID-19 outbreak. J Geriatr Psychiatry Neurol. 2022;35(2):196-205. doi: 10.1177/08919887221078559.	Unrelated
293	Kumar Gg S, Deepalam S, Siddiqui A, Adiga CP, Kumar S, Shivalingappa SS, Acharya UV, Goolahally LN, Sharma S, Andrew D, Hosmani P, Nair S, Medikeri G, Rao RM, Agadi JB, Kumar S, Adoor G, Sharma S, Hegde R, Saini J, Kulanthaivelu K. Coronavirus disease 2019 (COVID-19)-associated rhino-orbito-cerebral mucormycosis: A multi-institutional retrospective study of imaging patterns. World Neurosurg. 2022;162:e131-e140. doi: 10.1016/j.wneu.2022.02.107. Epub 2022 Mar 4.	Unrelated
294	Gollub RL. Brain changes after COVID revealed by imaging. Nature. 2022;604(7907):633-634. doi: 10.1038/d41586-022-00503-x.	Opinion
295	Hu J, Huang Y, Liu J, Zheng Z, Xu X, Zhou Y, Wang J.COVID-19 related stress and mental health outcomes 1 year after the peak of the pandemic outbreak in China: the mediating effect of resilience and social support. Front Psychiatry. 2022;13:828379. doi: 10.3389/fpsyt.2022.828379. eCollection 2022 Feb 21.	Unrelated
296	Bui MV, McInnes E, Ennis G, Foster K.Protocol for a mixed methods process evaluation of the Promoting Resilience in Nurses (PRiN) trial.Int J Ment Health Nurs. 2022;31(3):687-696. doi: 10.1111/inm.12989. Epub 2022 Mar 13.	Protocol
297	Molina JD, Rodrigo Holgado I, Juanes González A, Combarro Ripoll CE, Lora Pablos D, Rubio G, Alonso J, Rivas-Clemente FPJ. Neuropsychological symptom identification and classification in the hospitalized COVID-19 patients during the first wave of the pandemic in a front-line Spanish tertiary hospital. Front Psychiatry. 2022;13:838239. doi: 10.3389/fpsyt.2022.838239. eCollection 2022 Mar 2.	Unrelated
298	Liu X, Wen X, Zhang Q, Xu W. Buffering traumatic reactions to COVID-19: Mindfulness moderates the relationship between the severity of the pandemic and posttraumatic stress symptoms. Psychol Trauma. 2023;15(3):474-482. doi: 10.1037/tra0001227. Epub 2022 Mar 21.	Unrelated
299	McBride O, Bunting E, Harkin O, Butter S, Shevlin M, Murphy J, Mason L, Hartman TK, McKay R, Hyland P, Levita L, Bennett KM, Stocks TVA, Gibson-Miller J, Martinez AP, Vallières F, Bentall RP. Testing both affordability-availability and psychological-coping mechanisms underlying changes in alcohol use during the COVID-19 pandemic. PLoS One. 2022;17(3):e0265145. doi: 10.1371/journal.pone.0265145. eCollection 2022 Mar 24.	Unrelated
300	Ortoleva Bucher C, Abt M, Berthoud L, Cohen C. Health and coping strategies of nursing home residents and their relatives during the COVID-19 pandemic: a mixed-methods study protocol. BMJ Open. 2022;12(3):e059262. doi: 10.1136/bmjopen-2021-059262.	Protocol
301	Nelson DW, Granberg T, Andersen P, Jokhadar E, Kåhlin J, Granström A, Hallinder H, Schening A, Thunborg C, Walles H, Hagman G, Shams-Latifi R, Yu J, Petersson S, Tzortzakakis A, Levak N, Aspö M, Piehl F, Zetterberg H, Kivipelto M, Eriksson LI. The Karolinska NeuroCOVID study protocol: Neurocognitive impairment, biomarkers and advanced imaging in critical care survivors. Acta Anaesthesiol Scand. 2022;66(6):759-766. doi: 10.1111/aas.14062. Epub 2022 Apr 2.	**Included**
302	Zhen B, Yao B, Zhou X. Pandemic exposure, post-traumatic stress disorder, conflict behaviors, and online aggressive behaviors among college students during the COVID-19 pandemic: Examining the moderating role of gender. Front Psychiatry. 2022;13:809173. doi: 10.3389/fpsyt.2022.809173. eCollection 2022 Mar 17.	Unrelated
303	Kim D-Y, Pyo A, Ji S, You S-H, Kim S-E, Lim D, Kim H, Lee K-H, Oh S-J, Jung Y-R, Kim U-J, Jeon S, Kwon S-Y, Kang S-R, Lee H-B, Hyun H, Kim SY, Moon K-S, Lee S, Kang S-J, Min J-J. In vivo imaging of invasive aspergillosis with ^{18}F-fluorodeoxysorbitol positron emission tomography. Nat Commun. 2022;13(1):1926. doi: 10.1038/s41467-022-29553-5.	No COVID-19
304	Suo X, Zuo C, Lan H, Pan N, Zhang X, Kemp GJ, Wang S, Gong Q. COVID-19 vicarious traumatization links functional connectome to general distress. Neuroimage. 2022;255:119185. doi: 10.1016/j.neuroimage.2022.119185. Epub 2022 Apr 7.	Unrelated
305	Clark DE, Aggarwal SK, Phillips NJ, Soslow JH, Dendy JM, Hughes SG. Cardiac Magnetic Resonance in the Evaluation of COVID-19. Card Fail Rev. 2022;8:e09. doi: 10.15420/cfr.2021.20. eCollection 2022 Jan; Mar 23.	Review
306	Zare Dehnavi A, Salehi M, Arab Ahmadi M, Asgardoon MH, Ashrafi F, Ahmadinejad N, Behkar A, Hamidi Farahani R, Hashemi H, Tafakhori A, Shahali H, Rahmani M, Ranjbar Naeini A.Clinical, Laboratory and Imaging Characteristics of Hospitalized COVID-19 Patients with Neurologic Involvement; a Cross-Sectional Study. Arch Acad Emerg Med. 2022 Jan 30;10(1):e10. doi: 10.22037/aaem.v10i1.1507. eCollection 2022.PMID: 35402993 .	Unfocused
307	Tebbeb N, Villemagne F, Prieur T, Dorier S, Fort E, Célarier T, Fontana L, Barth N, Pélissier C.COVID-19 Health Crisis Workloads and Screening for Psychological Impact in Nursing Home Staff: A Qualitative and Quantitative Survey.Int J Environ Res Public Health. 2022 Mar 29;19(7):4061. doi: 10.3390/ijerph19074061.PMID: 35409741 .	No COVID-19
308	Andriuta D, Si-Ahmed C, Roussel M, Constans JM, Makki M, Aarabi A, Basille D, Andrejak C, Godefroy O. Clinical and Imaging Determinants of Neurocognitive Disorders in Post-Acute COVID-19 Patients with Cognitive Complaints. J Alzheimers Dis. 2022;87(3):1239-1250. doi: 10.3233/JAD-215506.	Included
309	Mungmunpuntipantip R, Wiwanitkit V. Re: Alterations in ^{18}F-FDG PET/MRI and ^{15}O-water PET brain findings in patients with neurological symptoms after COVID-19 vaccination: A pilot study. Clin Nucl Med. 2022;47(10):e671. doi: 10.1097/RLU.0000000000004227. Epub 2022 Apr 20.	Opinion
310	Frosolini A, Parrino D, Fabbris C, Fantin F, Inches I, Invitto S, Spinato G, Filippis C. Magnetic Resonance Imaging Confirmed Olfactory Bulb Reduction in Long COVID-19: Literature Review and Case Series. Brain Sci. 2022;12(4):430. doi: 10.3390/brainsci12040430.PMID: 35447962 .	Review
311	Siripongsatian D, Chotipanich C. Alterations in ^{18}F-FDG PET/MRI and ^{15}O-water PET brain findings in patients with neurological symptoms after COVID-19 vaccination: A pilot study: Reply. Clin Nucl Med. 2022;47(10):e671-e672. doi: 10.1097/RLU.0000000000004223. Epub 2022 Apr 22.	Opinion
312	Laroche A, Soulet D, Bazin M, Levesque T, Allaeys I, Vallières N, Gunzer M, Flamand L, Lacroix S, Boilard E.Live imaging of platelets and neutrophils during antibody-mediated neurovascular thrombosis.Blood Adv. 2022;6(12):3697-3702. doi: 10.1182/bloodadvances.2021006728.	No COVID-19
313	Yin Q, Chen A, Song X, Deng G, Dong W. Corrigendum: Risk Perception and PTSD Symptoms of Medical Staff Combating Against COVID-19: A PLS Structural Equation Model. Front Psychiatry. 2022;13:890074. doi: 10.3389/fpsyt.2022.890074. eCollection 2022 Apr 8.	Dupllicate of #128
314	Meena J, Dhiman S, Sharma R, Kumari K, Singhal S, Kulshrestha V, Vatsa R, Perumal V.The Impact of the COVID-19 Pandemic on the Psychological Well-being of Healthcare Workers in Obstetrics and Gynaecology: An Observational Study at an Apex Institute. Cureus. 2022;14(4):e24040. doi: 10.7759/cureus.24040. eCollection 2022 Apr 11.	Unrelated
315	Wang Y, Luo B, Hong B, Yang M, Zhao L, Jia P The relationship between family functioning and non-suicidal self-injury in adolescents: A structural equation modeling analysis. J Affect Disord. 2022;309:193-200. doi: 10.1016/j.jad.2022.04.124. Epub 2022 Apr 25.	No COVID-19
316	Tang W, Yan Z, Lu Y, Xu J. Prospective examination of adolescent emotional intelligence and post-traumatic growth during and after COVID-19 lockdown. Affect Disord. 2022;309:368-374. doi: 10.1016/j.jad.2022.04.129. Epub 2022 Apr 25.	Unrelated
317	Liu Y, Partinen E, Chan NY, Dauvilliers Y, Inoue Y, De Gennaro L, Plazzi G, Bolstad CJ, Nadorff MR, Merikanto I, Bjorvatn B, Han F, Zhang B, Cunha AS, Mota-Rolim S, Léger D, Matsui K, Espie CA, Chung F, Morin CM, Sieminski M, Penzel T, Holzinger B, Partinen M, Wing YK.Dream-enactment behaviours during the COVID-19 pandemic: an international COVID-19 sleep study. J Sleep Res. 2023;32(1):e13613. doi: 10.1111/jsr.13613. Epub 2022 Apr 26.	Unrelated
318	Adeyemo OO, Tu S, Falako S, Keene D. Stressors on frontline healthcare workers during the COVID-19 pandemic: a focus on moral injury and implications for the future. Int Arch Occup Environ Health. 2022;95(8):1755-1762. doi: 10.1007/s00420-022-01867-3. Epub 2022 Apr 28.	Unrelated

319	Cannon CEB, Ferreira R, Buttell F, O'Connor A. Impacts of the COVID-19 pandemic on rural survivors of intimate partner violence. Psychol Trauma. 2023;15(3):511-514. doi: 10.1037/tra0001244. Epub 2022 Apr 28.	Unrelated
320	Mennicken B, Petit G, Yombi JC, Belkhir L, Deschietere G, Germeau N, Salavrakos M, Moreau G, Nizet L, Cool G, Luts A, Billieux J, de Timary P. Psychological distress among hospital caregivers during and after the first wave of COVID-19: Individual factors involved in the severity of symptoms expression. Psychiatry Res Commun. 2022;2(2):100037. doi: 10.1016/j.psycom.2022.100037. Epub 2022 Apr 26.	Unrelated
321	Nam E, Kim Y. Resilience among residents from the areas first affected by COVID-19 in South Korea. Psychol Res Behav Manag. 2022;15:1033-1041. doi: 10.2147/PRBM.S364323. eCollection 2022 Apr 22.	Unrelated
322	Widemon RS, Huang S, Capaccione KM, Mitchell RP, Salvatore MM, Lignelli A, Nguyen P. The impact of the COVID-19 pandemic on neuroimaging volume in New York City. Neuroradiol J. 2022;35(6):713-717. doi: 10.1177/19714009221096828. Epub 2022 May 3.	**Included**
323	Mejia-Lancheros C, Alfayumi-Zeadna S, Lachaud J, O'Campo P, Gogosis E, Da Silva G, Stergiopoulos V, Hwang SW, Thulien N. Differential impacts of COVID-19 and associated responses on the health, social well-being and food security of users of supportive social and health programs during the COVID-19 pandemic: A qualitative study. Health Soc Care Community. 2022;30(6):e4332-e4344. doi: 10.1111/hsc.13826. Epub 2022 May 6.	Unrelated
324	Huang Y, Ling Q, Manyande A, Wu D, Xiang B. Brain imaging changes in patients recovered from COVID-19: A narrative review. Front Neurosci. 2022;16:855868. doi: 10.3389/fnins.2022.855868. eCollection 2022 Apr 22.	Review
325	Jovarauskaite L, Murphy D, Truskauskaite-Kuneviciene I, Dumarkaite A, Andersson G, Kazlauskas E. Associations between moral injury and ICD-11 post-traumatic stress disorder (PTSD) and complex PTSD among help-seeking nurses: a cross-sectional study. BMJ Open. 2022;12(5):e056289. doi: 10.1136/bmjopen-2021-056289.	Unrelated
326	Esteban-Sepúlveda S, Terradas-Robledo R, Castro-Ribeiro T, García-Pagès E, Sobregrau-Sangrà P, Lacueva-Pérez L. COVID-19 pandemic on health professionals in a third level hospital in Spain: job changes during the first wave, mental health at 4 months, and follow-up at 9 months. Enferm Clin (Engl Ed). 2022;32(3):143-151. doi: 10.1016/j.enfcle.2021.12.003. Epub 2022 May 10.	Unrelated
327	Sehgal A, Kumar J, Garg A, Jain A, Meher R, Singh M, Goel R. MR imaging spectrum in COVID associated Rhino-Orbito-Cerebral mucormycosis with special emphasis on intracranial disease and impact on patient prognosis. Eur J Radiol. 2022;152:110341. doi: 10.1016/j.ejrad.2022.110341. Epub 2022 May 6.	Review
328	Chen JJ, Zeng BY, Lui CC, Chen TY, Chen YW, Tseng PT. Pfizer-BioNTech COVID-19 vaccine-associated tinnitus and treatment with transcranial magnetic stimulation. QJM. 2022;115(9):623-624. doi: 10.1093/qjmed/hcac124.	No COVID-19
329	Jones DL, Zhang Y, Rodriguez VJ, Haberlen S, Ramirez C, Adimora AA, Merenstein D, Aouizerat B, Sharma A, Wilson T, Mimiaga MJ, Sheth AN, Plankey M, Cohen MH, Stosor V, Kempf MC, Friedman MR. Association of PTSD with longitudinal COVID-19 burden in a mixed-serostatus cohort of men and women: Weathering the storm. J Acquir Immune Defic Syndr. 2022;90(5):567-575. doi: 10.1097/QAI.0000000000003006.	Unrelated
330	Lysaught MT, Reece B, Grand Ortega MA, Guizado AV, Bustamante-Pixa C. Building caregiver resiliency in global health: Embodying the Catholic social tradition in the face of COVID-19. Linacre Q. 2022;89(2):184-205. doi: 10.1177/00243639221085041. Epub 2022 Mar 28.	Unrelated
331	Thomson G, Cook J, Nowland R, Donnellan WJ, Topalidou A, Jackson L, Fallon V. Resilience and post-traumatic growth in the transition to motherhood during the COVID-19 pandemic: A qualitative exploratory study. Scand J Caring Sci. 2022;36(4):1143-1155. doi: 10.1111/scs.13087. Epub 2022 May 27.	Unrelated
332	Piras I, Piazza MF, Piccolo C, Azara A, Piana A, Finco G, Galletta M. Experiences, emotions, and health consequences among COVID-19 survivors after intensive care unit hospitalization. Int J Environ Res Public Health. 2022;19(10):6263. doi: 10.3390/ijerph19106263.	Unrelated
333	Lersy F, Bund C, Anheim M, Mondino M, Noblet V, Lazzara S, Phillipps C, Collange O, Oulehri W, Mertes PM, Helms J, Merdji H, Schenck M, Schneider F, Pottecher J, Giraudeau C, Chammas A, Ardellier FD, Baloglu S, Ambarki K, Namer IJ, Kremer S. Evolution of neuroimaging findings in severe COVID-19 patients with initial neurological impairment: An observational study. Viruses. 2022;14(5):949. doi: 10.3390/v14050949.	**Included**
334	Miranda A, Bertoglio D, Stroobants S, Staelens S, Verhaeghe J. Spatiotemporal kernel reconstruction for linear parametric neurotransmitter PET kinetic modeling in motion correction brain PET of awake rats. Front Neurosci. 2022;16:901091. doi: 10.3389/fnins.2022.901091. eCollection 2022 May 12.	Animal
335	Keenan BE, Lacan F, Cooper A, Evans SL, Evans J. MRI safety, imaging artefacts, and grid distortion evaluated for FFP3 respiratory masks worn throughout the COVID-19 pandemic. Clin Radiol. 2022;77(8):e660-e666. doi: 10.1016/j.crad.2022.05.001. Epub 2022 May 16.	Unfocused
336	Zhou Q, Rao Q, Li H, Zhang M, Zhao X, Shi L, Ye C, Zhou X. Evaluation of injuries caused by coronavirus disease 2019 using multi-nuclei magnetic resonance imaging. Magn Reson Lett. 2021;1(1):2-10. doi: 10.1016/j.mrl.2021.100009. Epub 2021 Aug 8.	Review
337	Lee ZH, Chen IH. The association between problematic internet use, psychological distress, and sleep problems during COVID-19. Sleep Epidemiol. 2021;1:100005. doi: 10.1016/j.sleepe.2021.100005. Epub 2021 Sep 10.	Unrelated
338	Stram MN, Seifert AC, Cortes E, Akyatan A, Woodoff-Leith E, Borukhov V, Tetlow A, Alyemni D, Greenberg M, Gupta A, Krausert A, Mecca L, Rodriguez S, Stahl-Herz J, Guzman MA, Delman B, Crary JF, Dams-O'Connor K, Folkerth RD. Neuropathology of pediatric SARS-CoV-2 infection in the forensic setting: Novel application of *ex vivo* imaging in analysis of brain microvasculature. Front Neurol. 2022;13:894565. doi: 10.3389/fneur.2022.894565. eCollection 2022 May 24.	Unfocused
339	Wang J, Huang X, Wang Y, Wang M, Xu J, Li X. COVID-19 information overload, negative emotions and posttraumatic stress disorder: A cross-sectional study. Front Psychiatry. 2022;13:894174. doi: 10.3389/fpsyt.2022.894174. eCollection 2022 May 13.	Unrelated
340	de Paula JJ, Paiva RERP, Souza-Silva NG, Rosa DV, Duran FLS, Coimbra RS, Costa DS, Dutenhefner PR, Oliveira HSD, Camargos ST, Vasconcelos HMM, de Oliveira Carvalho N, da Silva JB, Silveira MB, Malamut C, Oliveira DM, Molinari LC, de Oliveira DB, Januário JN, Silva LC, De Marco LA, Queiroz DMM, Meira W, Busatto G, Miranda DM, Romano-Silva MA. Selective visuoconstructional impairment following mild COVID-19 with inflammatory and neuroimaging correlation findings. Mol Psychiatry. 2023;28(2):553-563. doi: 10.1038/s41380-022-01632-5. Epub 2022 Jun 14.	**Included**
341	Braule Pinto ALC, Guatimosim RF, Loureiro FF, Serpa ALO, Costa DS, Diaz AP, Joaquim RM, Baldaçara LR, Alves Maria GDS, da Silva AG, de Miranda DM, Malloy-Diniz LF. Longitudinal invariance and validity of the Impact of Event Scale-Revised under COVID-19 pandemic in a Brazilian sample. Psychol Assess. 2022;34(8):791-802. doi: 10.1037/pas0001152. Epub 2022 Jun 16.	Unrelated
342	Özümerzifon Y, Ross A, Brinza T, Gibney G, Garber CE. Exploring a dance/movement program on mental health and well-being in survivors of intimate partner violence during a pandemic. Front Psychiatry. 2022;13:887827. doi: 10.3389/fpsyt.2022.887827. eCollection 2022 May 26.	Unrelated
343	Ochnik D, Rogowska AM, Arzenšek A, Benatov J. Longitudinal predictors of coronavirus-related PTSD among young adults from Poland, Germany, Slovenia, and Israel. Int J Environ Res Public Health. 2022;19(12):7207. doi: 10.3390/ijerph19127207.	No neuroim.
344	Kim SH, Kang HS. Health promotion behaviors of pregnant couples in the COVID-19 pandemic: Actor-partner interdependence model. Int J Environ Res Public Health. 2022;19(12):7501. doi: 10.3390/ijerph19127501.	Unrelated
345	Jennings G, Monaghan A, Xue F, Duggan E, Romero-Ortuño R. Comprehensive clinical characterisation of brain fog in adults reporting long COVID symptoms. J Clin Med. 2022;11(12):3440. doi: 10.3390/jcm11123440.	No neuroim.
346	Li D, Xiong L, Pan G, Wang T, Li R, Zhu L, Tong Q, Yang Q, Peng Y, Zuo C, Wang C, Li X. Molecular imaging on ACE2-dependent transocular infection of coronavirus. J Med Virol. 2022;94(10):4878-4889. doi: 10.1002/jmv.27958. Epub 2022 Jul 5.	Unrelated

347	Morales Chainé S, Robles García R, López Montoya A, Bosch Maldonado A, Beristain Aguirre AG, Treviño Santa Cruz CL, Palafox GP, Lira Chávez IA, Barragán Torres L, Rangel Gómez MG. Screening tool for mental health problems during COVID-19 pandemic: Psychometrics and associations with sex, grieving, contagion, and seeking psychological care. Front Psychol. 2022;13:882573. doi: 10.3389/fpsyg.2022.882573. eCollection 2022 Jun 10.	Unrelated
348	Dagher GA, Ghanem AA, Haidar S, Kattouf N, Assaf M, Khdhir M, Chahine R, Rizk J, Makki M, Tamim H, Chebl RB. The prognostic value of biomarker levels and chest imaging in patients with COVID-19 presenting to the emergency department. Am J Emerg Med. 2022;59:15-23. doi: 10.1016/j.ajem.2022.06.043. Epub 2022 Jun 26.	No neuroim.
349	McCormack L, Lowe B. Making meaning of irreconcilable destruction of innocence: National humanitarian professionals exposed to cybercrime child sexual exploitation in the Philippines. Child Abuse Negl. 2022;131:105770. doi: 10.1016/j.chiabu.2022.105770. Epub 2022 Jun 27.	Unrelated
350	Araújo ST, Araújo CT, Silva E Castro R, de Oliveira Brügger LM, Souza E Silva NG, de Miranda DM, Simões E Silva AC. Imaging markers of neurologic damage in COVID-19: A systematic review. Curr Med Chem. 2023;30(9):1086-1106. doi: 10.2174/0929867329666220701124945.	Review
351	Slone M, Pe'er A, Mor F. Previous trauma exposure and self-mastery as moderators of psychiatric effects of home isolation during the Covid-19 pandemic: a field study. BMC Psychiatry. 2022;22(1):450. doi: 10.1186/s12888-022-04087-8.	Unrelated
352	Ferrajão P. Attachment mediates the impact of previous trauma on posttraumatic symptoms during the COVID-19 pandemic. Psychol Trauma. 2022. doi: 10.1037/tra0001312. Online ahead of print 2022 Jul 11.	Unrelated
353	Bradwell H, Edwards KJ, Winnington R, Thill S, Allgar V, Jones RB. Implementing affordable socially assistive pet robots in care homes before and during the COVID-19 pandemic: Stratified cluster randomized controlled trial and mixed methods study. JMIR Aging. 2022;5(3):e38864. doi: 10.2196/38864.	Unrelated
354	Zainudin NA, Zulkifli N, Hamid K, Hashim H, Mansor S. Experimental evaluation of absolute quantification in ^{99m} Tc-TRODAT-1 SPECT/CT brain dopamine transporter (DAT) studies. J Appl Clin Med Phys. 2022;23(8):e13723. doi: 10.1002/acm2.13723. Epub 2022 Jul 14.	Unrelated
355	Verger A, Barthel H, Tolboom N, Fraioli F, Cecchin D, Albert NL, van Berckel B, Boellaard R, Brendel M, Ekmekcioglu O, Semah F, Traub-Weidinger T, van de Weehaeghe D, Morbelli S, Guedj E. 2-[^{18}F]-FDG PET for imaging brain involvement in patients with long COVID: perspective of the EANM Neuroimaging Committee. Eur J Nucl Med Mol Imaging. 2022;49(11):3599-3606. doi: 10.1007/s00259-022-05913-7.	Review
356	Held ML, First JM, Huslage M. Effects of COVID-19, discrimination, and social support on Latinx adult mental health. J Immigr Minor Health. 2022;24(6):1446-1458. doi: 10.1007/s10903-022-01382-0. Epub 2022 Jul 16.	Unrelated
357	Bataineh MF, Mohamad MN, Al Dhaheri AS, Rawashdeh M, Al-Nawaiseh AM, Asali FF, Hashim M, Jarrar A, Abu Jamous DO, Stojanovska L, Daour RA, Saleh ST, Osaili TM, Cheikh Ismail L. Impact of coronavirus 2019 pandemic on post-traumatic stress disorder symptoms among pregnant women in Jordan. Womens Health (Lond). 2022;18:17455057221112935. doi: 10.1177/17455057221112935.	Unrelated
358	Morgan CA, Roberts RP, Chaffey T, Tahara-Eckl L, van der Meer M, Günther M, Anderson TJ, Cutfield NJ, Dalrymple-Alford JC, Kirk IJ, Rose Addis D, Tippett LJ, Melzer TR. Reproducibility and repeatability of magnetic resonance imaging in dementia. Phys Med. 2022;101:8-17. doi: 10.1016/j.ejmp.2022.06.012. Epub 2022 Jul 16.	No COVID-19
359	Sagherian K, Cho H, Steege LM. The insomnia, fatigue, and psychological well-being of hospital nurses 18 months after the COVID-19 pandemic began: A cross-sectional study. J Clin Nurs. 2022:10.1111/jocn.16451. doi: 10.1111/jocn.16451. Online ahead of print 2022 Jul 22.	Unrelated
360	Jeong J, Kim AR, Hilton C, Hong I. Impact of Event Scale-6 (IES-6) for U.S. adults who experienced the COVID-19 pandemic. BMC Psychiatry. 2022;22(1):490. doi: 10.1186/s12888-022-04136-2.	Unrelated
361	Shevorykin A, Carl E, Mahoney MC, Hanlon CA, Liskiewicz A, Rivard C, Alberico R, Belal A, Bensch L, Vantucci D, Thorner H, Marion M, Bickel WK, Sheffer CE. Transcranial magnetic stimulation for long-term smoking cessation: Preliminary examination of delay discounting as a therapeutic target and the effects of intensity and duration. Front Hum Neurosci. 2022;16:920383. doi: 10.3389/fnhum.2022.920383. eCollection 2022 Jul 5.	Unrelated
362	Goldstein E, Topitzes J, Brown RL, Jussel AB. Mental health among university employees during the COVID-19 pandemic: The role of previous life trauma and current posttraumatic stress symptoms. Psychol Trauma. 2023;15(3):536-545. doi: 10.1037/tra0001332. Epub 2022 Jul 25.	Unrelated
363	Salluzzi M, McCreary CR, Gobbi DG, Lauzon ML, Frayne R. Short-term repeatability and long-term reproducibility of quantitative MR imaging biomarkers in a single centre longitudinal study. Neuroimage. 2022;260:119488. doi: 10.1016/j.neuroimage.2022.119488. Epub 2022 Jul 22.	No COVID-19
364	He Z, Chiu JW, Lin Y, Akinwunmi B, Wong TH, Zhang CJP, Ming WK. The construct structures of psychological and behavioral responses to COVID-19 pandemic in pregnant women. Front Psychiatry. 2022;13:796567. doi: 10.3389/fpsyt.2022.796567. eCollection 2022 Jul 12.	Unrelated
365	Guedj E, Horowitz T. Brain metabolic PET findings on the long-term effects of COVID-19. J Nucl Med. 2022;63(9):1452-1453. doi: 10.2967/jnumed.122.264179. Epub 2022 Jul 29.	Opinion
366	Koliouli F, Issari P, Andrianakou M. Lived experiences, perceived positive outcomes, and post-traumatic growth among parents with children with epilepsy: A qualitative study. Epilepsy Behav. 2022;134:108862. doi: 10.1016/j.yebeh.2022.108862. Epub 2022 Jul 29.	Unrelated
367	Håkansson A, Cronhjort M, Lidin-Darlington P, Lilja G, Nilsson A, Schandl A, Friberg H. Cognitive behavioral therapy and acceptance and commitment therapy (CBT-ACT) vs. standard care after critical illness due to COVID-19: Protocol for a pilot randomized controlled trial. Front Psychiatry. 2022;13:907215. doi: 10.3389/fpsyt.2022.907215. eCollection 2022 Jul 15.	Unrelated
368	Nichter M. From idioms of distress, concern, and care to moral distress leading to moral injury in the time of Covid. Transcult Psychiatry. 2022;59(4):551-567. doi: 10.1177/13634615221115540. Epub 2022 Aug 8.	Opinion
369	El Tantawi M, Folayan MO, Nguyen AL, Aly NM, Ezechi O, Uzochukwu BSC, Alaba OA, Brown B. Validation of a COVID-19 mental health and wellness survey questionnaire. BMC Public Health. 2022;22(1):1509. doi: 10.1186/s12889-022-13825-2.	Unrelated
370	Sharma S, Qian W, Acharya BK, Kunwar D. Psychological status of Nepalese young adults during the end of first wave of COVID-19 pandemic. J Nepal Health Res Counc. 2022;20(1):166-172. doi: 10.33314/jnhrc.v20i01.3922.	Unrelated
371	Hsieh KY, Li DJ, Chou FH, Hsu ST, Wu HC, Chou LS, Wu PJ, Lin GG, Chen WJ, Liu CL, Huang JJ. Relationship of societal adaptation with vaccine worries among healthcare workers during the COVID-19 pandemic: The mediating effects of posttraumatic stress disorder. Int J Environ Res Public Health. 2022;19(15):9498. doi: 10.3390/ijerph19159498.	Unrelated
372	Giusti EM, Veronesi G, Callegari C, Castelnuovo G, Iacoviello L, Ferrario MM. The North Italian Longitudinal Study Assessing the Mental Health Effects of SARS-CoV-2 Pandemic Health Care Workers-Part II: Structural validity of scales assessing mental health. Int J Environ Res Public Health. 2022;19(15):9541. doi: 10.3390/ijerph19159541.	Unrelated
373	Bhattacharjee A, Purohit P, Roy PK. Neuroprotective drug discovery from phytochemicals and metabolites for CNS viral infection: A systems biology approach with clinical and imaging validation. Front Neurosci. 2022;16:917867. doi: 10.3389/fnins.2022.917867. eCollection 2022 Jul 25.	No COVID-19

374	Carvalho-Alves MO, Petrilli-Mazon VA, Brunoni AR, Malbergier A, Fukuti P, Polanczyk GV, Miguel EC, Corchs F, Wang YP. Dimensions of emotional distress among Brazilian workers in a COVID-19 reference hospital: A factor analytical study. World J Psychiatry. 2022;12(6):843-859. doi: 10.5498/wjp.v12.i6.843. eCollection 2022 Jun 19.	Unrelated
375	Callen AL, Tanabe J, Thaker AA, Pollard R, Sauer B, Jones W, Pattee J, Steach B, Timpone VM. Evaluation of cerebrovascular reactivity and vessel wall imaging in patients with prior COVID-19: A prospective case-control MRI study. AJR Am J Roentgenol. 2023;220(2):257-264. doi: 10.2214/AJR.22.28267. Epub 2022 Aug 24.	**Included**
376	Lucignani G, Guarnera A, Rossi-Espagnet MC, Moltoni G, Antonelli A, Figà Talamanca L, Carducci C, Calo Carducci FI, Napolitano A, Gandolfo C, Campi F, Auriti C, Parazzini C, Longo D. From fetal to neonatal neuroimaging in TORCH infections: A pictorial review. Children (Basel). 2022;9(8):1210. doi: 10.3390/children9081210.	Review
377	Smith CJ, Renshaw P, Yurgelun-Todd D, Sheth C. Acute and chronic neuropsychiatric symptoms in novel coronavirus disease 2019 (COVID-19) patients: A qualitative review. Front Public Health. 2022;10:772335. doi: 10.3389/fpubh.2022.772335. eCollection 2022 Aug 8.	Review
378	Bova SM, Serafini L, Capetti P, Dallapiccola AR, Doneda C, Gadda A, Lonoce L, Vittorini A, Mannarino S, Veggiotti P; Milan MIS-C Study Group. Neurological involvement in multisystem inflammatory syndrome in children: Clinical, electroencephalographic and magnetic resonance imaging peculiarities and therapeutic implications. An Italian single-center experience. Front Pediatr. 2022;10:932208. doi: 10.3389/fped.2022.932208. eCollection 2022 Aug 12.	No COVID-19
379	Cena L, Trainini A, Tralli N, Nodari LS, Iacona E, Ronconi L, Testoni I. The impact of the COVID-19 pandemic on perinatal loss experienced by the parental couple: Protocol for a mixed methods study. JMIR Res Protoc. 2022;11(9):e38866. doi: 10.2196/38866.	Protocol
380	Pan N, Qin K, Yu Y, Long Y, Zhang X, He M, Suo X, Zhang S, Sweeney JA, Wang S, Gong Q. Pre-COVID brain functional connectome features prospectively predict emergence of distress symptoms after onset of the COVID-19 pandemic. Psychol Med. 2022:1-12. doi: 10.1017/S0033291722002173. Online ahead of print 2022 Jul 26.	Unfocused
381	Lucidi S, Iannattone S, Mallardo F, Spoto A, Lorio R. Psychological distress in healthcare workers during the Covid-19 pandemic: Development and validation of the Italian version of the Healthcare Workers Emergency Distress Questionnaire (HEDQ). Clin Neuropsychiatry. 2022;19(4):206-219. doi: 10.36131/cnfioritieditore20220403.	Unrelated
382	Jakhar K, Lone KY, Gupta R, Srivastava A, Solanki HK. A cross-sectional study on the psychological impact of stress, anxiety and depressive symptoms in COVID-19 patients. Cureus. 2022;14(8):e27724. doi: 10.7759/cureus.27724. eCollection 2022 Aug 5.	Unrelated
383	Liu X, Wen X, Zhang Q, Xu W. "Buffering traumatic reactions to COVID-19: Mindfulness moderates the relationship between the severity of the pandemic and posttraumatic stress symptoms": Correction. Psychol Trauma. 2023;15(3):482. doi: 10.1037/tra0001384. Epub 2022 Sep 15.	Duplicate of #298
384	Bae S, Cho OH. Prevalence and risk factors of depression in patients with rheumatic disease in South Korea during the COVID-19 pandemic. Healthcare (Basel). 2022;10(9):1758. doi: 10.3390/healthcare10091758.	Unrelated
385	Stanizzo MR, Castelli L, Di Nardo C, Brunetti M, De Sanctis C, Ghiggia A. Psychological distress in breast cancer patients during the Italian COVID-19 pandemic. Int J Environ Res Public Health. 2022;19(18):11433. doi: 10.3390/ijerph191811433.	Unrelated
386	Smith PD, Groves AK, Langellier BA, Keene DE, Rosenberg A, Blankenship KM. Eviction, post-traumatic stress, and emergency department use among low-income individuals in New Haven, CT. Prev Med Rep. 2022;29:101956. doi: 10.1016/j.pmedr.2022.101956. eCollection 2022 Oct; Aug 17.	Unrelated
387	Dai Z, Wang H, Xiao W, Huang Y, Si M, Fu J, Chen X, Jia M, Leng Z, Cui D, Dong L, Mak WWS, Su X. The association of mindfulness and psychological well-being among individuals who have recovered from COVID-19 in Jianghan District, Wuhan, China: A cross-sectional study. J Affect Disord. 2022;319:437-445. doi: 10.1016/j.jad.2022.09.062. Epub 2022 Sep 23.	Unrelated
388	Feizi S, Elgar F, Lonergan M, Eisenberg K, Rahmouni N, Brunet A. Commentary: Pandemic exposure, post-traumatic stress disorder, conflict behaviors, and online aggressive behaviors among college students during the COVID-19 pandemic: Examining the moderating role of gender. Front Psychiatry. 2022;13:1006330. doi: 10.3389/fpsyt.2022.1006330. eCollection 2022 Sep 12.	Opinion
389	Dixon KE, Mahoney CT, Samuelson KW. Ongoing traumatic stress during a global pandemic. Psychol Trauma. 2022. doi: 10.1037/tra0001360. Online ahead of print 2022 Sep 29.	Unrelated
390	Chen S, Bi K, Lyu S, Sun P, Bonanno GA. Depression and PTSD in the aftermath of strict COVID-19 lockdowns: a cross-sectional and longitudinal network analysis. Eur J Psychotraumatol. 2022;13(2):2115635. doi: 10.1080/20008066.2022.2115635. eCollection 2022 Sep 22.	Unrelated
391	Wertheimer GSO, Brandão MB, Reis F. COVID-19-related acute necrotizing encephalopathy with new spectroscopy features. Rev Soc Bras Med Trop. 2022;55:e02752022. doi: 10.1590/0037-8682-0275-2022. eCollection 2022 Sep 30.	Case
392	Villarreal-Zegarra D, Paredes-Angeles R, Mayo-Puchoc N, Vilela-Estada AL, Copez-Lonzoy A, Huarcaya-Victoria J. An explanatory model of depressive symptoms from anxiety, post-traumatic stress, somatic symptoms, and symptom perception: the potential role of inflammatory markers in hospitalized COVID-19 patients. BMC Psychiatry. 2022;22(1):638. doi: 10.1186/s12888-022-04277-4.	Unfocused
393	Mediavilla R, McGreevy KR, Felez-Nobrega M, Monistrol-Mula A, Bravo-Ortiz MF, Bayón C, Rodríguez-Vega B, Nicaise P, Delaire A, Sijbrandij M, Witteveen AB, Purgato M, Barbui C, Tedeschi F, Melchior M, van der Waerden J, McDaid D, Park AL, Kalisch R, Petri-Romão P, Underhill J, Bryant RA, Haro JM, Ayuso-Mateos JL; RESPOND Consortium. Effectiveness of a stepped-care programme of internet-based psychological interventions for healthcare workers with psychological distress: Study protocol for the RESPOND healthcare workers randomised controlled trial. Digit Health. 2022;8:20552076221129084. doi: 10.1177/20552076221129084. eCollection 2022 Jan-Dec; Oct 5.	Protocol
394	Xie JQ, Zhang H, Zhang X, Yin MZ, Yang J, Chen K, Xiong JR, Chen YQ. The mediating role of personal values between COVID-19-related posttraumatic growth and life satisfaction among Chinese college students: A two-wave longitudinal study. Front Psychol. 2022;13:926375. doi: 10.3389/fpsyg.2022.926375. eCollection 2022 Sep 23.	Unrelated
395	Büttiker P, Stefano GB, Weissenberger S, Ptacek R, Anders M, Raboch J, Kream RM. HIV, HSV, SARS-CoV-2 and Ebola share long-term neuropsychiatric sequelae. Neuropsychiatr Dis Treat. 2022;18:2229-2237. doi: 10.2147/NDT.S382308. eCollection 2022 Oct 5.	Review
396	Elgendy M, Balaha HM, Shehata M, Alksas A, Ghoneim M, Sherif F, Mahmoud A, Elgarayhi A, Taher F, Sallah M, Ghazal M, El-Baz A. Role of Imaging and AI in the Evaluation of COVID-19 Infection: A Comprehensive Survey. Front Biosci (Landmark Ed). 2022;27(9):276. doi: 10.31083/j.fbl2709276.	Review
397	Morales Chainé S, Robles García R, Bosch A, Treviño Santa Cruz CL. Depressive, anxious, and post-traumatic stress symptoms related to violence during the COVID-19 pandemic, by sex, COVID-19 status, and intervention-seeking conditions among the general population. Int J Environ Res Public Health. 2022;19(19):12559. doi: 10.3390/ijerph191912559.	Unrelated
398	Ippolito A, Urban H, Ghoroghi K, Rosbach N, Lingwal N, Adam EH, Friedrichson B, Steinbicker AU, Hattingen E, Wenger KJ. Prevalence of acute neurological complications and pathological neuroimaging findings in critically ill COVID-19 patients with and without VV-ECMO treatment. Sci Rep. 2022;12(1):17423. doi: 10.1038/s41598-022-21475-y.	Unfocused
399	Shen R, Shen D, Zhou Q, Zhang M, Chen S. Antibody-mediated autoimmune encephalitis evaluated by ^{18}F-DPA714 PET/MRI. Brain Behav Immun Health. 2022;26:100535. doi: 10.1016/j.bbih.2022.100535. eCollection 2022 Dec; Oct 13.	Case
400	Scorza CA, Finsterer J, Scorza FA, de Almeida AG. Workup for cerebral involvement in COVID-19 requires cerebral imaging, electroencephalography, and cerebrospinal fluid studies. Int J Infect Dis. 2022;125:285-286. doi: 10.1016/j.ijid.2022.10.025. Epub 2022 Oct 21.	Opinion

401	Hegarty S, Lamb D, Stevelink SAM, Bhundia R, Raine R, Doherty MJ, Scott HR, Marie Rafferty A, Williamson V, Dorrington S, Hotopf M, Razavi R, Greenberg N, Wessely S. 'It hurts your heart': frontline healthcare worker experiences of moral injury during the COVID-19 pandemic. Eur J Psychotraumatol. 2022;13(2):2128028. doi: 10.1080/20008066.2022.2128028. eCollection 2022 Oct 18.	Unrelated
402	Liu Y, Zou L, Yan S, Zhang P, Zhang J, Wen J, Mao J, Li L, Wang Y, Fu W. Burnout and post-traumatic stress disorder symptoms among medical staff two years after the COVID-19 pandemic in Wuhan, China: Social support and resilience as mediators. J Affect Disord. 2023;321:126-133. doi: 10.1016/j.jad.2022.10.027. Epub 2022 Oct 21.	Unrelated
403	Ryk J, Simpson R, Hosseiny F, Notarianni M, Provencher MD, Rudnick A, Upshur R, Sud A. Virtually-delivered Sudarshan Kriya Yoga (SKY) for Canadian veterans with PTSD: A study protocol for a nation-wide effectiveness and implementation evaluation. PLoS One. 2022;17(10):e0275774. doi: 10.1371/journal.pone.0275774. eCollection 2022 Oct 26.	No neuroim.
404	Díez-Cirarda M, Yus M, Gómez-Ruiz N, Polidura C, Gil-Martínez L, Delgado-Alonso C, Jorquera M, Gómez-Pinedo U, Matias-Guiu J, Arrazola J, Matias-Guiu JA. Multimodal neuroimaging in post-COVID syndrome and correlation with cognition. Brain. 2022:awac384. doi: 10.1093/brain/awac384. Online ahead of print 2022 Oct 26.	**Included**
405	Bak S, Jeong Y, Yeu M, Jeong J. Brain-computer interface to predict impulse buying behavior using functional near-infrared spectroscopy. Sci Rep. 2022;12(1):18024. doi: 10.1038/s41598-022-22653-8.	No COVID-19
406	Sivanesan U, Wu K, McInnes MDF, Dhindsa K, Salehi F, van der Pol CB. Checklist for Artificial Intelligence in Medical Imaging reporting adherence in peer-reviewed and preprint manuscripts with the highest altmetric attention scores: A meta-research study. Can Assoc Radiol J. 2023;74(2):334-342. doi: 10.1177/08465371221134056. Epub 2022 Oct 27.	Unrelated
407	Jiang J, Han P, Huang X, Liu Y, Shao H, Zeng L, Duan X. Post-traumatic growth experience of first-line emergency nurses infected with COVID-19 during the epidemic period-A qualitative study in Shanghai, China. Front Public Health. 2022;10:1015316. doi: 10.3389/fpubh.2022.1015316. eCollection 2022 Oct 12.	Unrelated
408	Faretta E, Maslovaric G, Garau MI, Marmondi G, Piras L, Rezzola S, Incerti A, Nardoni A, Pagani M, Gallina E. The psychological impact of the COVID emergency on Italian nursing homes staff and the effectiveness of eye movement desensitization and reprocessing. Front Psychol. 2022;13:969028. doi: 10.3389/fpsyg.2022.969028. eCollection 2022 Oct 12.	Unrelated
409	Annam S, Fleming MF, Gulraiz A, Zafar MT, Khan S, Oghomitse-Omene PT, Saleemuddin S, Patel P, Ahsan Z, Qavi MSS. The impact of COVID-19 on the behaviors and attitudes of children and adolescents: A cross-sectional study. Cureus. 2022;14(9):e29719. doi: 10.7759/cureus.29719. eCollection 2022 Sep 28.	No neuroim.
410	Goehringer F, Bruyere A, Doyen M, Bevilacqua S, Charmillon A, Heyer S, Verger A. Brain ^{18}F-FDG PET imaging in outpatients with post-COVID-19 conditions: findings and associations with clinical characteristics. Eur J Nucl Med Mol Imaging. 2023;50(4):1084-1089. doi: 10.1007/s00259-022-06013-2. Epub 2022 Nov 2.	**Included**
411	Zawilska JB, Kuczyńska K. Psychiatric and neurological complications of long COVID. J Psychiatr Res. 2022;156:349-360. doi: 10.1016/j.jpsychires.2022.10.045. Epub 2022 Oct 20.	Review
412	Rogier G, Zobel SB, Rizzi D, Velotti P. Post-traumatic stress disorder and alcohol use disorder during the COVID-19 outbreak: Do dissociation and emotional metacognitive beliefs mediate the role of emotion dysregulation? Psychiatry Investig. 2022;19(10):803-813. doi: 10.30773/pi.2022.0013. Epub 2022 Oct 21.	Unrelated
413	Dai B, Zhang X, Meng G, Zheng Y, Hu K, Li Q, Liu X. The mechanism of governments' and individuals' influence on protective behaviours during the second wave of COVID-19: a multiple mediation model. Eur J Psychotraumatol. 2022;13(2):1-12. doi: 10.1080/20008066.2022.2135196. eCollection 2022 Nov 1.	Unrelated
414	Kalvesmaki AF, Gonzales E, George RT, Nguyen H, Pugh MJ. Post-9/11 veterans perceptions of the pandemic: Areas of greatest impact on health and well-being. PEC Innov. 2022;1:100096. doi: 10.1016/j.pecinn.2022.100096. Epub 2022 Nov 4.	Unrelated
415	Aljaberi MA, Alareqe NA, Alsalahi A, Qasem MA, Noman S, Uzir MUH, Mohammed LA, Fares ZEA, Lin CY, Abdallah AM, Hamat RA, Mohd Rani MD. A cross-sectional study on the impact of the COVID-19 pandemic on psychological outcomes: Multiple indicators and multiple causes modeling. PLoS One. 2022;17(11):e0277368. doi: 10.1371/journal.pone.0277368. eCollection 2022 Nov 9.	Unrelated
416	Nascimento MCD, Fava SMCL, Dázio EMR, Silva SAD, Silva KL, Oliveira AB, Delpino FM, Fronteira I, Arcêncio RA, Sawada NO. Association between biopsychosocial factors and self-reported COVID-19 clinical complications in a Brazilian city. Rev Bras Epidemiol. 2022;25:e220033. doi: 10.1590/1980-549720220033. eCollection 2022 Nov 7.	Unrelated
417	Saggu S, Chen Y, Cottingham C, Rehman H, Wang H, Zhang S, Augelli-Szafran C, Lu S, Lambert N, Jiao K, Lu XY, Wang Q. Activation of a novel α2AAR-spinophilin-cofilin axis determines the effect of α2 adrenergic drugs on fear memory reconsolidation. Mol Psychiatry. 2023;28(2):588-600. doi: 10.1038/s41380-022-01851-w. Epub 2022 Nov 10.	Animal
418	Carola V, Vincenzo C, Morale C, Cecchi V, Rocco M, Nicolais G. Psychological health in intensive care unit health care workers after the COVID-19 pandemic. Healthcare (Basel). 2022;10(11):2201. doi: 10.3390/healthcare10112201.	Unrelated
419	Wu CY, Huang SM, Lin YH, Hsieh HH, Chu LWL, Yang HC, Chiu SC, Peng SL. Reproducibility of diffusion tensor imaging-derived parameters: implications for the streptozotocin-induced type 1 diabetic rats. MAGMA. 2022. doi: 10.1007/s10334-022-01048-w. Online ahead of print 2022 Nov 15.	Animal
420	Zelter PM, Zeleva OV, Sidorov EA, Solovov DV, Surovtsev EN. Multimodal imaging in rhinoorbitocerebral mucormycosis associated with type 2 diabetes after COVID-19. Rambam Maimonides Med J. 2022;13(4):e0024. doi: 10.5041/RMMJ.10483.	Case
421	Kumar I, Verma A, Dangwal J, Singh PK, Chandra Shukla R, Chakravarty J. Magnetic resonance imaging spectrum of COVID-associated rhino-orbital-cerebral mucormycosis and assessment of anatomical severity. Neuroradiol J. 2022:19714009221114442. doi: 10.1177/19714009221114442. Online ahead of print 2022 Nov 21.	Unfocused
422	Çetin H, Ateş AŞ, Taydaş O, Elmas B, Güçlü E. Magnetic resonance imaging findings in COVID-19-related anosmia. Turk J Med Sci. 2022;52(5):1506-1512. doi: 10.55730/1300-0144.5490. Epub 2022 Oct 19.	Unfocused
423	Tsouvelas G, Kalaitzaki A, Tamiolaki A, Rovithis M, Konstantakopoulos G. Secondary traumatic stress and dissociative coping strategies in nurses during the COVID-19 pandemic: The protective role of resilience. Arch Psychiatr Nurs. 2022;41:264-270. doi: 10.1016/j.apnu.2022.08.010. Epub 2022 Aug 31.	Unrelated
424	Rodríguez-Martín B, Ramírez-Moreno JM, Caro-Alonso PÁ, Novo A, Martínez-Andrés M, Clavijo-Chamorro MZ, Rodríguez-Almagro J, López-Espuela F. The psychological impact on frontline nurses in Spain of caring for people with COVID-19. Arch Psychiatr Nurs. 2022;41:27-34. doi: 10.1016/j.apnu.2022.07.022. Epub 2022 Jul 19.	Unrelated
425	Jacobs J, Vu P, Liu AK. A patient with myelin oligodendrocyte glycoprotein positive encephalitis with ring enhancing lesions on magnetic resonance imaging (MRI) after COVID-19 exposure. Cureus. 2022;14(11):e31844. doi: 10.7759/cureus.31844. eCollection 2022 Nov 23.	Case
426	Tepmongkol S, Suwanwela NC, Hirankarn N, Tangwongchai S, Vorasayan P, Sukprakun C, Siritaranon N, Kijpaisalratana N, Akarathanawat W, Chutinet A, Tantivatana J. Brain perfusion single photon emission computed tomography abnormality in MRI-negative stroke-like patients post COVID-19 vaccination. Medicine (Baltimore). 2022;101(47):e31965. doi: 10.1097/MD.0000000000031965.	Unfocused
427	Tam CC, Qiao S, Garrett C, Zhang R, Aghaei A, Aggarwal A, Li X. Substance use, psychiatric symptoms, personal mastery, and social support among COVID-19 long haulers: A compensatory model. medRxiv. 2022:2022.11.23.22282679. doi: 10.1101/2022.11.23.22282679. Preprint 2022 Nov 24.	No neuroim.
428	Flaus A, Deddah T, Reilhac A, Leiris N, Janier M, Merida I, Grenier T, McGinnity CJ, Hammers A, Lartizien C, Costes N. PET image enhancement using artificial intelligence for better characterization of epilepsy lesions. Front Med (Lausanne). 2022;9:1042706. doi: 10.3389/fmed.2022.1042706. eCollection 2022 Nov 16.	No COVID-19

429	Meneguzzo P, De Mico A, Gori P, Ajello A, Ceccato E, Consolati MD, Vita A, Sala A, Santonastaso P. A multicenter network analysis examining the psychological effects of the COVID-19 pandemic on patients with eating disorders versus their healthy siblings. J Clin Med. 2022;11(23):7187. doi: 10.3390/jcm11237187.	No neuroim.
430	England AE, Lietzau S, Vu J, Hebert C, Banayan D, Greenberg JA, Gerhart J. Piloting group-based behavioral activation therapy for families of deceased COVID-19 patients. Crit Care Explor. 2022;4(12):e0803. doi: 10.1097/CCE.0000000000000803. eCollection 2022 Dec 5.	Unrelated
431	Kaya AT, Akman B. Is a high chest CT severity score a risk factor for an increased incidence of long-term neuroimaging findings after COVID-19? J Stroke Cerebrovasc Dis. 2023;32(2):106920. doi: 10.1016/j.jstrokecerebrovasdis.2022.106920. Epub 2022 Nov 30.	Unsuitable
432	Najafi MA, Zandifar A, Ramezani N, Paydari H, Kheradmand M, Ansari B, Najafi MR, Hajiahmadi S, Khorvash F, Saadatnia M, Vossough A. Clinical and neuroimaging characteristics of ischemic stroke in rhino-orbito-cerebral mucormycosis associated with COVID-19. Clin Neuroradiol. 2022:1-9. doi: 10.1007/s00062-022-01238-y. Online ahead of print 2022 Dec 15.	Unfocused
433	Rossi AA, Panzeri A, Taccini F, Parola A, Mannarini S. The Rising of the Shield hero. Development of the Post-Traumatic Symptom Questionnaire (PTSQ) and Assessment of the Protective Effect of self-esteem from trauma-related Anxiety and Depression. J Child Adolesc Trauma. 2022:1-19. doi: 10.1007/s40653-022-00503-z. Online ahead of print 2022 Dec 7.	Unrelated
434	Laksmita OD, Chung MH, Shieh YY, Chang PC. Psychometric Validation of the Indonesian Version of Children's Revised Impact of Event Scale 13. Int J Environ Res Public Health. 2022;19(24):17069. doi: 10.3390/ijerph192417069.	Unrelated
435	Shan D, Li S, Xu R, Nie G, Xie Y, Han J, Gao X, Zheng Y, Xu Z, Dai Z. Post-COVID-19 human memory impairment: A PRISMA-based systematic review of evidence from brain imaging studies. Front Aging Neurosci. 2022;14:1077384. doi: 10.3389/fnagi.2022.1077384. eCollection 2022 Dec 9.	Review
436	Riaz BK, Islam MZ, Ahmed HU, Akhtar K, Haque A, Amin KMB, Mahmood F, Refat MNH, Islam F. Post-traumatic stress disorders and coping strategies of health professionals during COVID-19 pandemic in Bangladesh: findings of a countrywide cross-sectional study. Lancet Reg Health Southeast Asia. 2023;11:100131. doi: 10.1016/j.lansea.2022.100131. Epub 2022 Dec 23.	Unrelated
437	Solmi M, Thompson T, Estradé A, Agorastos A, Radua J, Cortese S, Dragioti E, Leisch F, Vancampfort D, Thygesen LC, Aschauer H, Schlögelhofer M, Aschauer E, Schneeberger A, Huber CG, Hasler G, Conus P, Do Cuénod KQ, von Känel R, Arrondo G, Fusar-Poli P, Gorwood P, Llorca PM, Krebs MO, Scanferla E, Kishimoto T, Rabbani G, Skonieczna-Żydecka K, Brambilla P, Favaro A, Takamiya A, Zoccante L, Colizzi M, Bourgin J, Kamiński K, Moghadasin M, Seedat S, Matthews E, Wells J, Vassilopoulou E, Gadelha A, Su KP, Kwon JS, Kim M, Lee TY, Papsuev O, Manková D, Boscutti A, Gerunda C, Saccon D, Righi E, Monaco F, Croatto G, Cereda G, Demurtas J, Brondino N, Veronese N, Enrico P, Politi P, Ciappolino V, Pfennig A, Bechdolf A, Meyer-Lindenberg A, Kahl KG, Domschke K, Bauer M, Koutsouleris N, Winter S, Borgwardt S, Bitter I, Balazs J, Czobor P, Unoka Z, Mavridis D, Tsamakis K, Bozikas VP, Tunvirachaisakul C, Maes M, Rungnirundorn T, Supasitthumrong T, Haque A, Brunoni AR, Costardi CG, Schuch FB, Polanczyk G, Luiz JM, Fonseca L, Aparicio LV, Valvassori SS, Nordentoft M, Vendsborg P, Hoffmann SH, Sehli J, Sartorius N, Heuss S, Guinart D, Hamilton J, Kane J, Rubio J, Sand M, Koyanagi A, Solanes A, Andreu-Bernabeu A, Cáceres ASJ, Arango C, Díaz-Caneja CM, Hidalgo-Mazzei D, Vieta E, Gonzalez-Peñas J, Fortea L, Parellada M, Fullana MA, Verdolini N, Andrlíková E, Janků K, Millan MJ, Honciuc M, Moniuszko-Malinowska A, Łoniewski I, Samochowiec J, Kiszkiel Ł, Marlicz M, Sowa P, Marlicz W, Spies G, Stubbs B, Firth J, Sullivan S, Darcin AE, Aksu H, Dilbaz N, Noyan O, Kitazawa M, Kurokawa S, Tazawa Y, Anselmi A, Cracco C, Machado AI, Estrade N, De Leo D, Curtis J, Berk M, Ward P, Teasdale S, Rosenbaum S, Marx W, Horodnic AV, Oprea L, Alexinschi O, Ifteni P, Turliuc S, Ciuhodaru T, Bolos A, Matei V, Nieman DH, Sommer I, van Os J, van Amelsvoort T, Sun CF, Guu TW, Jiao C, Zhang J, Fan J, Zou L, Yu X, Chi X, de Timary P, van Winkel R, Ng B, Pena E, Arellano R, Roman R, Sanchez T, Movina L, Morgado P, Brissos S, Aizberg O, Mosina A, Krinitski D, Mugisha J, Sadeghi-Bahmani D, Sheybani F, Sadeghi M, Hadi S, Brand S, Errazuriz A, Crossley N, Ristic DI, López-Jaramillo C, Efthymiou D, Kuttichira P, Kallivayalil RA, Javed A, Afridi MI, James B, Seb-Akahomen OJ, Fiedorowicz J, Carvalho AF, Daskalakis J, Yatham LN, Yang L, Okasha T, Dahdouh A, Gerdle B, Tiihonen J, Shin JI, Lee J, Mhalla A, Gaha L, Brahim T, Altynbekov K, Negay N, Nurmagambetova S, Jamei YA, Weiser M, Correll CU. Validation of the Collaborative Outcomes study on Health and Functioning during Infection Times (COH-FIT) questionnaire for adults. J Affect Disord. 2023;326:249-261. doi: 10.1016/j.jad.2022.12.022. Epub 2022 Dec 28. PMID: 36586617; PMCID: PMC9794522.	Unrelated
438	Hallaç S, Kaya B. İşbirliğine dayali, entegre bir ruh sağliği yaklaşimi: “Kademeli Bakim Modeli” [A collaborative, integrative mental health approach: "the Stepped Care Model"]. Türk Psikiyatri Derg. 2022;33(4):293-294. doi: 10.5080/u27236. English, Turkish.	Opinion
439	Park CL, Wilt JA, Russell BS, Fendrich M. Does perceived post-traumatic growth during the COVID-19 pandemic reflect actual positive changes? Anxiety Stress Coping. 2023:1-13. doi: 10.1080/10615806.2022.2157821. Online ahead of print 2023 Jan 2.	Unrelated
440	Li X, Yin W, Li A, Li D, Gao X, Wang R, Cui B, Qiu S, Li R, Jia L, Zuo C, Zhang L, Li M. ACE2 PET to reveal the dynamic patterns of ACE2 recovery in an infection model with pseudocorona virus. J Med Virol. 2023;95(2):e28470. doi: 10.1002/jmv.28470.	Animal
441	Noda Y, Sato A, Shichi M, Sato A, Fujii K, Iwasa M, Nagano Y, Kitahata R, Osawa R. Real world research on transcranial magnetic stimulation treatment strategies for neuropsychiatric symptoms with long-COVID in Japan. Asian J Psychiatr. 2023;81:103438. doi: 10.1016/j.ajp.2022.103438. Epub 2022 Dec 28.	Unfocused
442	Lobanov-Rostovsky S, Kiss L. The mental health and well-being of internally displaced female Yazidis in the Kurdistan Region of Iraq: a realist review of psychosocial interventions and the impact of COVID-19. Glob Ment Health (Camb). 2022;9:508-520. doi: 10.1017/gmh.2022.55. eCollection 2022 Nov 4.	Review
443	Wang S, Zhao Y, Li J. True grit and brain: Trait grit mediates the connection of DLPFC functional connectivity density to posttraumatic growth following COVID-19. J Affect Disord. 2023;325:313-320. doi: 10.1016/j.jad.2023.01.022. Epub 2023 Jan 7.	FC
444	Paolini M, Palladini M, Mazza MG, Colombo F, Vai B, Rovere-Querini P, Falini A, Poletti S, Benedetti F. Brain correlates of subjective cognitive complaints in COVID-19 survivors: A multimodal magnetic resonance imaging study. Eur Neuropsychopharmacol. 2023;68:1-10. doi: 10.1016/j.euroneuro.2022.12.002. Epub 2022 Dec 12.	**Included**
445	Goodman ML, Molldrem S, Elliott A, Robertson D, Keiser P. Long COVID and mental health correlates: a new chronic condition fits existing patterns. Health Psychol Behav Med. 2023;11(1):2164498. doi: 10.1080/21642850.2022.2164498. eCollection 2023 Jan 8.	No neuroim.
446	Tanwar M, Singhal A, Alizadeh M, Sotoudeh H. Magnetic Resonance Imaging (MRI) findings in COVID-19 associated encephalitis. Neurol Int. 2023;15(1):55-68. doi: 10.3390/neurolint15010005.	Unfocused
447	Malan-Müller S, Valles-Colomer M, Palomo T, Leza JC. The gut-microbiota-brain axis in a Spanish population in the aftermath of the COVID-19 pandemic: microbiota composition linked to anxiety, trauma, and depression profiles. Gut Microbes. 2023;15(1):2162306. doi: 10.1080/19490976.2022.2162306.	Unrelated
448	Aktan Ç, Toptan T, Utku Ç, Bolay H. Female migraine patients had lower COVID-19 phobia and PCL-5 scores during the lockdown period. Acta Neurol Belg. 2023:1-8. doi: 10.1007/s13760-023-02175-4. Online ahead of print 2023 Jan 19.	Unrelated
449	Tang YM, Wu TL, Liu HT. Causal model analysis of the effect of formalism, fear of infection, COVID-19 stress on firefighters' post-traumatic stress syndrome and insomnia. Int J Environ Res Public Health. 2023;20(2):1097. doi: 10.3390/ijerph20021097.	Unrelated
450	Gray SAO, Moberg SA, Obus EA, Parker V, Rosenblum KL, Muzik M, Zeanah CH, Drury SS. Harnessing virtual mom power: Process and outcomes of a pilot telehealth adaptation of a multifamily, attachment-based intervention. J Infant Child Adolesc Psychother. 2022;21(1):6-18. doi: 10.1080/15289168.2022.2045464. Epub 2022 Mar 14.	Unrelated
451	Ferrucci R, Cuffaro L, Capozza A, Rosci C, Maiorana N, Groppo E, Reitano MR, Poletti B, Ticozzi N, Tagliabue L, Silani V, Priori A. Brain positron emission tomography (PET) and cognitive abnormalities one year after COVID-19. J Neurol. 2023;270(4):1823-1834. doi: 10.1007/s00415-022-11543-8. Epub 2023 Jan 24.	Case

452	Yang F, Huang N, Zhang B, Fu M, Guo J. Network analysis of COVID-19-related PTSD and depressive symptoms among Chinese general population and comorbidity subgroup. Psychol Trauma. 2023;15(3):431-442. doi: 10.1037/tra0001434. Epub 2023 Jan 30.	Unrelated
453	Jungmann SM, Piefke M, Nin V, Asmundson GJG, Witthöft M. COVID-19 stress syndrome in the German general population: Validation of a German version of the COVID Stress Scales. PLoS One. 2023;18(2):e0279319. doi: 10.1371/journal.pone.0279319. eCollection 2023 Feb 2.	Unrelated
454	Horn M, Wathelet M, Amad A, Martignène N, Lathiere T, Khelfaoui K, Rousselle M, El Qaoubii O, Vuotto F, Faure K, Creupelandt C, Vaiva G, Fovet T, D'Hondt F. Persistent physical symptoms after COVID-19 infection and the risk of Somatic Symptom Disorder. J Psychosom Res. 2023;166:111172. doi: 10.1016/j.jpsychores.2023.111172. Epub 2023 Jan 25.	No neuroim.
455	Purgato M, Turrini G, Tedeschi F, Serra R, Tarsitani L, Compri B, Muriago G, Cadorin C, Ostuzzi G, Nicaise P, Lorant V, Sijbrandij M, Witteveen AB, Ayuso-Mateos JL, Mediavilla R, Haro JM, Felez-Nobrega M, Figueiredo N, Pollice G, McDaid D, Park AL, Kalisch R, Petri-Romão P, Underhill J, Bryant RA, Nosè M, Barbui C. Effectiveness of a stepped-care programme of WHO psychological interventions in migrant populations resettled in Italy: Study protocol for the RESPOND randomized controlled trial. Front Public Health. 2023;11:1100546. doi: 10.3389/fpubh.2023.1100546. eCollection 2023 Jan 25.	Protocol
456	Papp L, Rasul S, Spielvogel CP, Krajnc D, Poetsch N, Woehrer A, Patronas EM, Ecsedi B, Furtner J, Mitterhauser M, Rausch I, Widhalm G, Beyer T, Hacker M, Traub-Weidinger T. Sex-specific radiomic features of L-[S-methyl-^{11}C] methionine PET in patients with newly-diagnosed gliomas in relation to IDH1 predictability. Front Oncol. 2023;13:986788. doi: 10.3389/fonc.2023.986788. eCollection 2023 Feb 3.	Unrelated
457	García FE, Vergara-Barra P, Concha-Ponce P, Andrades M, Rincón P, Valdivia-Devia M. The Emotion Regulation Questionnaire: Psychometric properties and prediction of posttraumatic consequences during the COVID-19 pandemic in Chilean adults. Int J Environ Res Public Health. 2023;20(4):3452. doi: 10.3390/ijerph20043452.	Unrelated
458	Okrzeja J, Garkowski A, Kubas B, Moniuszko-Malinowska A. Imaging and neuropathological findings in patients with Post COVID-19 Neurological Syndrome-A review. Front Neurol. 2023;14:1136348. doi: 10.3389/fneur.2023.1136348. eCollection 2023 Feb 9.	Review
459	Su P, Yi J, Chen X, Xiao Y. Visual analysis of psychological resilience research based on Web of Science database. Psychol Res Behav Manag. 2023;16:465-481. doi: 10.2147/PRBM.S394693. eCollection 2023 Feb 19.	Unrelated
460	Calkins K, Guttormson J, McAndrew NS, Losurdo H, Loonsfoot D, Schmitz S, Fitzgerald J. The early impact of COVID-19 on intensive care nurses' personal and professional well-being: A qualitative study. Intensive Crit Care Nurs. 2023;76:103388. doi: 10.1016/j.iccn.2023.103388. Online ahead of print 2023 Jan 16.	Unrelated
461	Hwang S, Kwon KT, Lee SH, Kim SW, Chang HH, Kim Y, Bae S, Cheong HS, Park SY, Kim B, Lee S, Park J, Heo ST, Oh WS, Kim Y, Park KH, Kang CK, Oh N, Lim SJ, Yun S, Son JW, Ryoo HW, Chung US, Lee JY, Park HY, Shin JY, Bae SG, Lee JY. Correlates of burnout among healthcare workers during the COVID-19 pandemic in South Korea. Sci Rep. 2023;13(1):3360. doi: 10.1038/s41598-023-30372-x.	Unrelated
462	Ohtake M, Suenaga J, Akimoto T, Ikeuchi H, Muroya A, Ohata H, Kubota Y, Chiku M, Hamano T, Yamamoto T. Magnetic resonance imaging scan of the brain after mild COVID-19 infection. Cureus. 2023;15(1):e34229. doi: 10.7759/cureus.34229. eCollection 2023 Jan 26.	Unfocused
463	Martin JL, DeViva J, McCarthy E, Gehrman P, Josephson K, Mitchell M, Beer C, Runnals J. In-person and telehealth treatment of veterans with insomnia disorder using cognitive behavioral therapy for insomnia during the COVID-19 pandemic. J Clin Sleep Med. 2023. doi: 10.5664/jcsm.10540. Online ahead of print 2023 Mar 2.	No neuroim.
464	Sasaki N, Yamatoku M, Tsuchida T, Sato H, Yamaguchi K. Effect of repetitive transcranial magnetic stimulation on long coronavirus disease 2019 with fatigue and cognitive dysfunction. Prog Rehabil Med. 2023;8:20230004. doi: 10.2490/prm.20230004. eCollection Feb 28 2023.	Unfocused
465	Mahajan A, Nayak MK, Gaikwad SB, Sharma K, Padma Srivastava MV, Anand P, Oinam R, Mishra BM. Post-vaccination/post-COVID immune-mediated demyelination of the brain and spinal cord: A novel neuroimaging finding. Neurol India. 2023;71(1):86-91. doi: 10.4103/0028-3886.370449.	Case
466	Zhao YJ, Zhang C, Guo T, Sha S, Su Z, Cheung T, Jackson T, An FR, Xiang YT. Associations between post-traumatic stress symptoms and quality of life among psychiatric healthcare personnel in China during the COVID-19 pandemic: A network approach. Front Psychiatry. 2023;14:975443. doi: 10.3389/fpsyt.2023.975443. eCollection 2023 Feb 15.	No neuroim.
467	Kamasak B, Ulcay T, Nisari M, Gorgulu O, Akca V, Alpaslan M, Yetis A, Hizmali L, Karahocagil MK, Aycan K. Effects of COVID-19 on brain and cerebellum: a voxel based morphometrical analysis. Bratisl Lek Listy. 2023. doi: 10.4149/BLL_2023_068. Online ahead of print 2023 Feb 28.	**Included**
468	Young P, Appel L, Tolf A, Kosmidis S, Burman J, Rieckmann A, Schöll M, Lubberink M. Image-derived input functions from dynamic ^{15}O-water PET scans using penalised reconstruction. EJNMMI Phys. 2023;10(1):15. doi: 10.1186/s40658-023-00535-w. Online 2023 Mar 7.	No COVID-19
469	Carmassi C, Pedrinelli V, Antonini C, Dell'Oste V, Gravina D, Nardi B, Bertelloni CA, Massimetti G, Nieto-Munuera J, Dell'Osso L. Validation of the Spanish Version of the Trauma and Loss Spectrum Self-Report (TALS-SR): A study on healthcare workers facing the COVID-19 pandemic. Neuropsychiatr Dis Treat. 2023;19:495-506. doi: 10.2147/NDT.S396540. eCollection 2023 Mar 3.	Unrelated
470	Patel H, Easterbrook B, D'Alessandro-Lowe AM, Andrews K, Ritchie K, Hosseiny F, Rodrigues S, Malain A, O'Connor C, Schielke H, McCabe RE, Nicholson AA, Lanius R, McKinnon MC. Associations between trauma and substance use among healthcare workers and public safety personnel during the SARS-CoV-2 (COVID-19) pandemic: the mediating roles of dissociation and emotion dysregulation. Eur J Psychotraumatol. 2023;14(1):2180706. doi: 10.1080/20008066.2023.2180706.	Unrelated
471	Doukas A, DePierro J, Starkweather S, Sharma V, Marin DB, Charney DS. Symptom characteristics of health care workers seeking outpatient psychiatric care during the COVID-19 pandemic. Am J Ind Med. 2023. doi: 10.1002/ajim.23478. Online ahead of print 2023 Mar 24.	Unrelated
472	McCarthy L, Khegai O, Goldstein J, Belani P, Pawha P, Kihira S, Mathew B, Gururangan K, Hao Q, Singh A, Navis A, Delman BN, Jette N, Balchandani P. Neuroimaging findings and neurological manifestations in hospitalized COVID-19 patients: Impact of cancer and ventilatory support status. PLoS One. 2023;18(3):e0283614. doi: 10.1371/journal.pone.0283614. eCollection 2023 Mar 24.	Unrelated
473	Xiao W, Liu X, Wang H, Huang Y, Dai Z, Si M, Fu J, Chen X, Jia M, Leng Z, Cui D, Mak WWS, Dong L, Su X. Mediating role of resilience in the relationship between COVID-19 related stigma and mental health among COVID-19 survivors: a cross-sectional study. Infect Dis Poverty. 2023;12(1):27. doi: 10.1186/s40249-023-01074-3.	Unrelated
474	Ozery-Flato M, Ein-Dor L, Pinchasov O, Dabush-Kasa M, Hexter E, Chodick G, Rosen-Zvi M, Guindy M. The impact of COVID-19 pandemic on clinical findings in medical imaging exams: An observational study in a nationwide Israeli health organization. JMIR Form Res. 2023. doi: 10.2196/42930. Online ahead of print 2023 Feb 28.	Unsuitable
475	Pountney J, Butcher I, Donnelly P, Morrison R, Shaw RL. How the COVID-19 crisis affected the well-being of nurses working in paediatric critical care: A qualitative study. Br J Health Psychol. 2023. doi: 10.1111/bjhp.12661. Online ahead of print 2023 Mar 30.	No neuroim.
476	Chang CH, Chen SJ, Chen YC, Tsai HC. A 30-year-old woman with an 8-week history of anxiety, depression, insomnia, and mild cognitive impairment following COVID-19 who responded to accelerated bilateral theta-burst transcranial magnetic stimulation over the prefrontal cortex. Am J Case Rep. 2023;24:e938732. doi: 10.12659/AJCR.938732.	Case

477	Klinkhammer S, Horn J, Duits AA, Visser-Meily JMA, Verwijk E, Slooter AJC, Postma AA, van Heugten CM; NeNeSCo study group. Neurological and (neuro)psychological sequelae in intensive care and general ward COVID-19 survivors. Eur J Neurol. 2023. doi: 10.1111/ene.15812. Online ahead of print 2023 Apr 3.	**Included**
478	Wischmann J, Bartos LM, Brendel M, Albert NL, Forbrig R, Straube A, Masouris I. Translocator protein (TSPO)-PET as diagnostic and monitoring tool in COVID-19 related MRI-negative brainstem encephalitis: a case report. J Neurol. 2023:1-4. doi: 10.1007/s00415-023-11691-5. Online ahead of print 2023 Apr 3.	Case
479	Bawand R, Ghiasian M, Samadyan M. Concurrent diffuse dural and leptomeningeal enhancements in brain Magnetic Resonance Imaging following a mild COVID-19 infection: A Novel Case Report and Review of Literature. Curr Med Imaging. 2023. doi: 10.2174/1573405620666230328110847. Online ahead of print 2023Mar 28.	Case
480	Patil C, Kumar A, Battula V, Kumar P, Kollu R, Kotamraju S, Nethi Balingari BL, Reddy S, Ravula S, Reddy AR. Radiological manifestations of rhino-orbito-cranial mucormycosis in COVID-19 patients correlated with pathological and clinical outcomes and emphasis on magnetic resonance imaging-based scoring system. Cureus. 2023;15(3):e35745. doi: 10.7759/cureus.35745. eCollection 2023 Mar.	Unfocused
481	Caille A, Allemang-Trivalle A, Blanchin M, Rebion A, Sauvaget A, Gohier B, Birmes P, Bui E, Fakra E, Krebs MO, Lemogne C, Prieto N, Jalenques I, Vidailhet P, Aouizerate B, Hingray C, El-Hage W. EMDR for symptoms of depression, stress and burnout in health care workers exposed to COVID-19 (HARD): A study protocol for a trial within a cohort study. Eur J Psychotraumatol. 2023;14(1):2179569. doi: 10.1080/20008066.2023.2179569.	Protocol
482	Burulday V, Bayar Muluk N, Akgül MH, Sayar MS. Diffusion-weighted imaging measurements of central smell regions in COVID-19 patients: insular gyrus, corpus amygdala, and thalamus. Eur Rev Med Pharmacol Sci. 2023;27(7):3201-3207. doi: 10.26355/eurrev_202304_31954.	**Included**
483	Gruenebach N, Abello Mercado MA, Grauhan NF, Sanner A, Kronfeld A, Groppa S, Schoeffling VI, Hilbert T, Brockmann MA, Othman AE. Clinical feasibility and validation of the accelerated T2 mapping sequence GRAPPATINI in brain imaging. Heliyon. 2023;9(4):e15064. doi: 10.1016/j.heliyon.2023.e15064.	No COVID-19
484	McGinty G, Fox R, Hyland P. Assessing prevalence, validity, and correlates of ICD-11 posttraumatic stress disorder and complex posttraumatic stress disorder in Ireland. Psychol Trauma. 2023. doi: 10.1037/tra0001472. Epub ahead of print 2023 Apr 24.	Unrelated
485	Debs P, Khalili N, Solnes L, Al-Zaghal A, Sair HI, Yedavalli V, Luna LP. Post-COVID-19 brain [18F] FDG-PET findings: A retrospective single-center study in the United States. AJNR Am J Neuroradiol. 2023. doi: 10.3174/ajnr.A7863. Epub ahead of print 2023 Apr 27.	**Included**
486	Rong J, Zhao C, Xia X, Li G, Haider A, Wei H, Chen J, Xiao Z, Li Y, Zhou X, Xu H, Collier TL, Wang L, Liang SH. Evaluation of [18F]favipiravir in rodents and nonhuman primates (NHP) with positron emission tomography. Pharmaceuticals (Basel). 2023;16(4):524. doi: 10.3390/ph16040524. Online 2023 Mar 31.	Animal
487	Douaud G, Lee S, Alfaro-Almagro F, Arthofer C, Wang C, McCarthy P, Lange F, Andersson JLR, Griffanti L, Duff E, Jbabdi S, Taschler B, Keating P, Winkler AM, Collins R, Matthews PM, Allen N, Miller KL, Nichols TE, Smith SM. SARS-CoV-2 is associated with changes in brain structure in UK Biobank. Nature. 2022;604(7907):697-707. doi: 10.1038/s41586-022-04569-5. Epub 2022 Mar 7.	**Included**
488	Nawabi J, Morotti A, Wildgruber M, Boulouis G, Kraehling H, Schlunk F, Can E, Kniep H, Thomalla G, Psychogios M, Hamm B, Fiehler J, Hanning U, Sporns P. Clinical and imaging characteristics in patients with SARS-CoV-2 infection and acute intracranial hemorrhage. J Clin Med. 2020;9(8):2543. doi: 10.3390/jcm9082543.	Unsuitable

Included (n = 36)
Excluded (n = 452)
- Unrelated (n = 199)
- Reviews (n = 68)
- Unfocused (n = 37)
- Case reports/series (n = 37)
- No COVID-19 (n = 29)
- No neuroimaging (n = 18)
- Unsuitable data presentation (n = 18)
- Opinion (n = 17)
- Protocols (n = 13)
- Animal (n = 10)
- Functional connectivity only (n = 3)
- fMRI only (n = 1)
- Duplicates (n = 2)
- *In vitro* (n = 0)
- Post-mortem (n = 0)

Supplementary Table S2. PRISMA Checklist.

PRISMA 2020 Checklist

Section and Topic	Item #	Checklist item	Location where item is reported
**TITLE**			1
Title	1	Identify the report as a systematic review.	1
**ABSTRACT**			
Abstract	2	See the PRISMA 2020 for Abstracts checklist.	2
**INTRODUCTION**			
Rationale	3	Describe the rationale for the review in the context of existing knowledge.	3
Objectives	4	Provide an explicit statement of the objective(s) or question(s) the review addresses.	3
**METHODS**			4
Eligibility criteria	5	Specify the inclusion and exclusion criteria for the review and how studies were grouped for the syntheses.	4
Information sources	6	Specify all databases, registers, websites, organisations, reference lists and other sources searched or consulted to identify studies. Specify the date when each source was last searched or consulted.	4, Fig. 1
Search strategy	7	Present the full search strategies for all databases, registers and websites, including any filters and limits used.	4, Fig. 1, 5
Selection process	8	Specify the methods used to decide whether a study met the inclusion criteria of the review, including how many reviewers screened each record and each report retrieved, whether they worked independently, and if applicable, details of automation tools used in the process.	4
Data collection process	9	Specify the methods used to collect data from reports, including how many reviewers collected data from each report, whether they worked independently, any processes for obtaining or confirming data from study investigators, and if applicable, details of automation tools used in the process.	4
Data items	10a	List and define all outcomes for which data were sought. Specify whether all results that were compatible with each outcome domain in each study were sought (e.g. for all measures, time points, analyses), and if not, the methods used to decide which results to collect.	4
	10b	List and define all other variables for which data were sought (e.g. participant and intervention characteristics, funding sources). Describe any assumptions made about any missing or unclear information.	Table 1, 5-16
Study risk of bias assessment	11	Specify the methods used to assess risk of bias in the included studies, including details of the tool(s) used, how many reviewers assessed each study and whether they worked independently, and if applicable, details of automation tools used in the process.	AXIS, 4, Suppl.
Effect measures	12	Specify for each outcome the effect measure(s) (e.g. risk ratio, mean difference) used in the synthesis or presentation of results.	N/A
Synthesis methods	13a	Describe the processes used to decide which studies were eligible for each synthesis (e.g. tabulating the study intervention characteristics and comparing against the planned groups for each synthesis (item #5)).	Table 1, 5-16
	13b	Describe any methods required to prepare the data for presentation or synthesis, such as handling of missing summary statistics, or data conversions.	N/A
	13c	Describe any methods used to tabulate or visually display results of individual studies and syntheses.	Table 1, 5-16
	13d	Describe any methods used to synthesize results and provide a rationale for the choice(s). If meta-analysis was performed, describe the model(s), method(s) to identify the presence and extent of statistical heterogeneity, and software package(s) used.	3-16
	13e	Describe any methods used to explore possible causes of heterogeneity among study results (e.g. subgroup analysis, meta-regression).	16, N/A
	13f	Describe any sensitivity analyses conducted to assess robustness of the synthesized results.	N/A
Reporting bias assessment	14	Describe any methods used to assess risk of bias due to missing results in a synthesis (arising from reporting biases).	AXIS, 4 Suppl.
Certainty assessment	15	Describe any methods used to assess certainty (or confidence) in the body of evidence for an outcome.	N/A
**RESULTS**			5-16
Study selection	16a	Describe the results of the search and selection process, from the number of records identified in the search to the number of studies included in the review, ideally using a flow diagram.	3-5
	16b	Cite studies that might appear to meet the inclusion criteria, but which were excluded, and explain why they were excluded.	Fig.1, 5, Suppl.
Study characteristics	17	Cite each included study and present its characteristics.	5-16
Risk of bias in	18	Present assessments of risk of bias for each included study.	N/A

Section and Topic	Item #	Checklist item	Location where item is reported
studies			
Results of individual studies	19	For all outcomes, present, for each study: (a) summary statistics for each group (where appropriate) and (b) an effect estimate and its precision (e.g. confidence/credible interval), ideally using structured tables or plots.	N/A
Results of syntheses	20a	For each synthesis, briefly summarise the characteristics and risk of bias among contributing studies.	N/A
	20b	Present results of all statistical syntheses conducted. If meta-analysis was done, present for each the summary estimate and its precision (e.g. confidence/credible interval) and measures of statistical heterogeneity. If comparing groups, describe the direction of the effect.	N/A
	20c	Present results of all investigations of possible causes of heterogeneity among study results.	N/A
	20d	Present results of all sensitivity analyses conducted to assess the robustness of the synthesized results.	N/A
Reporting biases	21	Present assessments of risk of bias due to missing results (arising from reporting biases) for each synthesis assessed.	N/A
Certainty of evidence	22	Present assessments of certainty (or confidence) in the body of evidence for each outcome assessed.	N/A
**DISCUSSION**			16-17
Discussion	23a	Provide a general interpretation of the results in the context of other evidence.	16-17
	23b	Discuss any limitations of the evidence included in the review.	16-17
	23c	Discuss any limitations of the review processes used.	N/A
	23d	Discuss implications of the results for practice, policy, and future research.	17
**OTHER INFORMATION**			
Registration and protocol	24a	Provide registration information for the review, including register name and registration number, or state that the review was not registered.	Not registered
	24b	Indicate where the review protocol can be accessed, or state that a protocol was not prepared.	N/A
	24c	Describe and explain any amendments to information provided at registration or in the protocol.	N/A
Support	25	Describe sources of financial or non-financial support for the review, and the role of the funders or sponsors in the review.	N/A
Competing interests	26	Declare any competing interests of review authors.	17
Availability of data, code and other materials	27	Report which of the following are publicly available and where they can be found: template data collection forms; data extracted from included studies; data used for all analyses; analytic code; any other materials used in the review.	N/A

From: Page MJ, McKenzie JE, Bossuyt PM, Boutron I, Hoffmann TC, Mulrow CD, et al. The PRISMA 2020 statement: an updated guideline for reporting systematic reviews. BMJ 2021;372:n71. doi: 10.1136/bmj.n71

For more information, visit: http://www.prisma-statement.org/

Supplementary Table S3. Quality assessment through AXIS. [Bracketed numbers] refer to papers' References; N/A, not applicable
Appraisal tool for Cross-Sectional Studies (AXIS) – Kremer et al. 2020 [30]

	Question	Yes	No	Don't know/Comment
Introduction				
1	Were the aims/objectives of the study clear?	×		
Methods				
2	Was the study design appropriate for the stated aim(s)?	×		
3	Was the sample size justified?	×		
4	Was the target/reference population clearly defined? (Is it clear who the research was about?)	×		
5	Was the sample frame taken from an appropriate population base so that it closely represented the target/reference population under investigation?			×
6	Was the selection process likely to select subjects/participants that were representative of the target/reference population under investigation?	×		
7	Were measures undertaken to address and categorise non-responders?			N/A
8	Were the risk factor and outcome variables measured appropriate to the aims of the study?	×		
9	Were the risk factor and outcome variables measured correctly using instruments/measurements that had been trialled, piloted or published previously?			×
10	Is it clear what was used to determined statistical significance and/or precision estimates? (e.g. p-values, confidence intervals)	×		
11	Were the methods (including statistical methods) sufficiently described to enable them to be repeated?	×		
Results				
12	Were the basic data adequately described?	×		
13	Does the response rate raise concerns about non-response bias?			N/A
14	If appropriate, was information about non-responders described?			N/A
15	Were the results internally consistent?	×		
16	Were the results presented for all the analyses described in the methods?	×		

Discussion				
17	Were the authors' discussions and conclusions justified by the results?	×		
18	Were the limitations of the study discussed?	×		
Other				
19	Were there any funding sources or conflicts of interest that may affect the authors' interpretation of the results?		×	
20	Was ethical approval or consent of participants attained?	×		

Appraisal tool for Cross-Sectional Studies (AXIS) – Klironomos et al. 2020 [31]

	Question	Yes	No	Don't know/Comment
Introduction				
1	Were the aims/objectives of the study clear?	×		
Methods				
2	Was the study design appropriate for the stated aim(s)?	×		
3	Was the sample size justified?	×		
4	Was the target/reference population clearly defined? (Is it clear who the research was about?)	×		
5	Was the sample frame taken from an appropriate population base so that it closely represented the target/reference population under investigation?	×		
6	Was the selection process likely to select subjects/participants that were representative of the target/reference population under investigation?	×		
7	Were measures undertaken to address and categorise non-responders?			N/A
8	Were the risk factor and outcome variables measured appropriate to the aims of the study?	×		
9	Were the risk factor and outcome variables measured correctly using instruments/measurements that had been trialled, piloted or published previously?	×		
10	Is it clear what was used to determined statistical significance and/or precision estimates? (e.g. p-values, confidence intervals)	×		
11	Were the methods (including statistical methods) sufficiently described to enable them to be repeated?	×		
Results				
12	Were the basic data adequately described?	×		
13	Does the response rate raise concerns about non-response bias?			N/A
14	If appropriate, was information about non-responders described?			N/A
15	Were the results internally consistent?	×		
16	Were the results presented for all the analyses described in the methods?	×		
Discussion				
17	Were the authors' discussions and conclusions justified by the results?	×		
18	Were the limitations of the study discussed?	×		
Other				
19	Were there any funding sources or conflicts of interest that may affect the authors' interpretation of the results?		×	
20	Was ethical approval or consent of participants attained?	×		

Appraisal tool for Cross-Sectional Studies (AXIS) – O'Shea et al. 2021 [32]

	Question	Yes	No	Don't know/Comment
Introduction				
1	Were the aims/objectives of the study clear?	×		
Methods				
2	Was the study design appropriate for the stated aim(s)?	×		
3	Was the sample size justified?	×		
4	Was the target/reference population clearly defined? (Is it clear who the research was about?)	×		
5	Was the sample frame taken from an appropriate population base so that it closely represented the target/reference population under investigation?	×		
6	Was the selection process likely to select subjects/participants that were representative of the target/reference population under investigation?	×		
7	Were measures undertaken to address and categorise non-responders?			N/A
8	Were the risk factor and outcome variables measured appropriate to the aims of the study?	×		
9	Were the risk factor and outcome variables measured correctly using instruments/measurements that had been trialled, piloted or published previously?	×		
10	Is it clear what was used to determined statistical significance and/or precision estimates? (e.g. p-values, confidence intervals)	×		
11	Were the methods (including statistical methods) sufficiently described to enable them to be repeated?	×		
Results				
12	Were the basic data adequately described?	×		
13	Does the response rate raise concerns about non-response bias?			N/A
14	If appropriate, was information about non-responders described?			N/A
15	Were the results internally consistent?	×		
16	Were the results presented for all the analyses described in the methods?			
Discussion				
17	Were the authors' discussions and conclusions justified by the results?	×		
18	Were the limitations of the study discussed?	×		
Other				
19	Were there any funding sources or conflicts of interest that may affect the authors' interpretation of the results?		×	
20	Was ethical approval or consent of participants attained?		×	

Appraisal tool for Cross-Sectional Studies (AXIS) – Lin et al. 2020 [33]

	Question	Yes	No	Don't know/Comment
Introduction				
1	Were the aims/objectives of the study clear?	×		
Methods				
2	Was the study design appropriate for the stated aim(s)?	×		

3	Was the sample size justified?	×		
4	Was the target/reference population clearly defined? (Is it clear who the research was about?)	×		
5	Was the sample frame taken from an appropriate population base so that it closely represented the target/reference population under investigation?	×		
6	Was the selection process likely to select subjects/participants that were representative of the target/reference population under investigation?	×		
7	Were measures undertaken to address and categorise non-responders?			N/A
8	Were the risk factor and outcome variables measured appropriate to the aims of the study?	×		
9	Were the risk factor and outcome variables measured correctly using instruments/measurements that had been trialled, piloted or published previously?	×		
10	Is it clear what was used to determined statistical significance and/or precision estimates? (e.g. p-values, confidence intervals)	×		
11	Were the methods (including statistical methods) sufficiently described to enable them to be repeated?	×		
Results				
12	Were the basic data adequately described?	×		
13	Does the response rate raise concerns about non-response bias?			N/A
14	If appropriate, was information about non-responders described?			N/A
15	Were the results internally consistent?	×		
16	Were the results presented for all the analyses described in the methods?	×		
Discussion				
17	Were the authors' discussions and conclusions justified by the results?	×		
18	Were the limitations of the study discussed?	×		
Other				
19	Were there any funding sources or conflicts of interest that may affect the authors’ interpretation of the results?		×	
20	Was ethical approval or consent of participants attained?		×	

Appraisal tool for Cross-Sectional Studies (AXIS) – Sawlani et al. 2021 [34]

	Question	Yes	No	Don’t know/Comment
Introduction				
1	Were the aims/objectives of the study clear?	×		
Methods				
2	Was the study design appropriate for the stated aim(s)?	×		
3	Was the sample size justified?	×		
4	Was the target/reference population clearly defined? (Is it clear who the research was about?)	×		
5	Was the sample frame taken from an appropriate population base so that it closely represented the target/reference population under investigation?	×		
6	Was the selection process likely to select subjects/participants that were representative of the target/reference population under investigation?	×		
7	Were measures undertaken to address and categorise non-responders?	×		
8	Were the risk factor and outcome variables measured appropriate to the aims of the study?	×		
9	Were the risk factor and outcome variables measured correctly using instruments/measurements that had been trialled, piloted or published previously?	×		
10	Is it clear what was used to determined statistical significance and/or precision estimates? (e.g. p-values, confidence intervals)	×		
11	Were the methods (including statistical methods) sufficiently described to enable them to be repeated?	×		
Results				
12	Were the basic data adequately described?	×		
13	Does the response rate raise concerns about non-response bias?			N/A
14	If appropriate, was information about non-responders described?			N/A
15	Were the results internally consistent?	×		
16	Were the results presented for all the analyses described in the methods?	×		
Discussion				
17	Were the authors' discussions and conclusions justified by the results?	×		
18	Were the limitations of the study discussed?	×		
Other				
19	Were there any funding sources or conflicts of interest that may affect the authors’ interpretation of the results?		×	
20	Was ethical approval or consent of participants attained?	×		

Appraisal tool for Cross-Sectional Studies (AXIS) – Lindan et al. 2021 [35]

	Question	Yes	No	Don’t know/Comment
Introduction				
1	Were the aims/objectives of the study clear?	×		
Methods				
2	Was the study design appropriate for the stated aim(s)?	×		
3	Was the sample size justified?	×		
4	Was the target/reference population clearly defined? (Is it clear who the research was about?)	×		
5	Was the sample frame taken from an appropriate population base so that it closely represented the target/reference population under investigation?	×		
6	Was the selection process likely to select subjects/participants that were representative of the target/reference population under investigation?	×		
7	Were measures undertaken to address and categorise non-responders?	×		
8	Were the risk factor and outcome variables measured appropriate to the aims of the study?	×		
9	Were the risk factor and outcome variables measured correctly using instruments/measurements that had been trialled, piloted or published previously?	×		
10	Is it clear what was used to determined statistical significance and/or precision estimates? (e.g. p-values, confidence intervals)	×		

11	Were the methods (including statistical methods) sufficiently described to enable them to be repeated?	×		
Results				
12	Were the basic data adequately described?	×		
13	Does the response rate raise concerns about non-response bias?			N/A
14	If appropriate, was information about non-responders described?			N/A
15	Were the results internally consistent?	×		
16	Were the results presented for all the analyses described in the methods?	×		
Discussion				
17	Were the authors' discussions and conclusions justified by the results?	×		
18	Were the limitations of the study discussed?	×		
Other				
19	Were there any funding sources or conflicts of interest that may affect the authors' interpretation of the results?		×	
20	Was ethical approval or consent of participants attained?	×		

Appraisal tool for Cross-Sectional Studies (AXIS) – Orman et al. 2021 [36]

	Question	Yes	No	Don't know/Comment
Introduction				
1	Were the aims/objectives of the study clear?	×		
Methods				
2	Was the study design appropriate for the stated aim(s)?	×		
3	Was the sample size justified?		×	
4	Was the target/reference population clearly defined? (Is it clear who the research was about?)	×		
5	Was the sample frame taken from an appropriate population base so that it closely represented the target/reference population under investigation?	×		
6	Was the selection process likely to select subjects/participants that were representative of the target/reference population under investigation?	×		
7	Were measures undertaken to address and categorise non-responders?			N/A
8	Were the risk factor and outcome variables measured appropriate to the aims of the study?	×		
9	Were the risk factor and outcome variables measured correctly using instruments/measurements that had been trialled, piloted or published previously?	×		
10	Is it clear what was used to determined statistical significance and/or precision estimates? (e.g. p-values, confidence intervals)	×		
11	Were the methods (including statistical methods) sufficiently described to enable them to be repeated?	×		
Results				
12	Were the basic data adequately described?	×		
13	Does the response rate raise concerns about non-response bias?			N/A
14	If appropriate, was information about non-responders described?			N/A
15	Were the results internally consistent?	×		
16	Were the results presented for all the analyses described in the methods?	×		
Discussion				
17	Were the authors' discussions and conclusions justified by the results?	×		
18	Were the limitations of the study discussed?	×		
Other				
19	Were there any funding sources or conflicts of interest that may affect the authors' interpretation of the results?		×	
20	Was ethical approval or consent of participants attained?	×		

Appraisal tool for Cross-Sectional Studies (AXIS) – Rapalino et al. 2021 [37]

	Question	Yes	No	Don't know/Comment
Introduction				
1	Were the aims/objectives of the study clear?	×		
Methods				
2	Was the study design appropriate for the stated aim(s)?	×		
3	Was the sample size justified?	×		
4	Was the target/reference population clearly defined? (Is it clear who the research was about?)	×		
5	Was the sample frame taken from an appropriate population base so that it closely represented the target/reference population under investigation?	×		
6	Was the selection process likely to select subjects/participants that were representative of the target/reference population under investigation?	×		
7	Were measures undertaken to address and categorise non-responders?			N/A
8	Were the risk factor and outcome variables measured appropriate to the aims of the study?	×		
9	Were the risk factor and outcome variables measured correctly using instruments/measurements that had been trialled, piloted or published previously?	×		
10	Is it clear what was used to determined statistical significance and/or precision estimates? (e.g. p-values, confidence intervals)	×		
11	Were the methods (including statistical methods) sufficiently described to enable them to be repeated?	×		
Results				
12	Were the basic data adequately described?	×		
13	Does the response rate raise concerns about non-response bias?			N/A
14	If appropriate, was information about non-responders described?			N/A
15	Were the results internally consistent?	×		
16	Were the results presented for all the analyses described in the methods?	×		
Discussion				
17	Were the authors' discussions and conclusions justified by the results?	×		
18	Were the limitations of the study discussed?	×		
Other				

19	Were there any funding sources or conflicts of interest that may affect the authors’ interpretation of the results?		×	
20	Was ethical approval or consent of participants attained?	×		

Appraisal tool for Cross-Sectional Studies (AXIS) – Kas et al. 2021 [38]

	Question	Yes	No	Don’t know/Comment
Introduction				
1	Were the aims/objectives of the study clear?	×		
Methods				
2	Was the study design appropriate for the stated aim(s)?	×		
3	Was the sample size justified?		×	
4	Was the target/reference population clearly defined? (Is it clear who the research was about?)	×		
5	Was the sample frame taken from an appropriate population base so that it closely represented the target/reference population under investigation?	×		
6	Was the selection process likely to select subjects/participants that were representative of the target/reference population under investigation?			×
7	Were measures undertaken to address and categorise non-responders?			N/A
8	Were the risk factor and outcome variables measured appropriate to the aims of the study?	×		
9	Were the risk factor and outcome variables measured correctly using instruments/measurements that had been trialled, piloted or published previously?			N/A
10	Is it clear what was used to determined statistical significance and/or precision estimates? (e.g. p-values, confidence intervals)		×	
11	Were the methods (including statistical methods) sufficiently described to enable them to be repeated?	×		
Results				
12	Were the basic data adequately described?	×		
13	Does the response rate raise concerns about non-response bias?			N/A
14	If appropriate, was information about non-responders described?			N/A
15	Were the results internally consistent?	×		
16	Were the results presented for all the analyses described in the methods?	×		
Discussion				
17	Were the authors' discussions and conclusions justified by the results?	×		
18	Were the limitations of the study discussed?		×	
Other				
19	Were there any funding sources or conflicts of interest that may affect the authors’ interpretation of the results?		×	
20	Was ethical approval or consent of participants attained?	×		

Appraisal tool for Cross-Sectional Studies (AXIS) – Conklin et al. 2021 [39]

	Question	Yes	No	Don’t know/Comment
Introduction				
1	Were the aims/objectives of the study clear?	×		
Methods				
2	Was the study design appropriate for the stated aim(s)?	×		
3	Was the sample size justified?	×		
4	Was the target/reference population clearly defined? (Is it clear who the research was about?)	×		
5	Was the sample frame taken from an appropriate population base so that it closely represented the target/reference population under investigation?	×		
6	Was the selection process likely to select subjects/participants that were representative of the target/reference population under investigation?	×		
7	Were measures undertaken to address and categorise non-responders?			N/A
8	Were the risk factor and outcome variables measured appropriate to the aims of the study?	×		
9	Were the risk factor and outcome variables measured correctly using instruments/measurements that had been trialled, piloted or published previously?	×		
10	Is it clear what was used to determined statistical significance and/or precision estimates? (e.g. p-values, confidence intervals)	×		
11	Were the methods (including statistical methods) sufficiently described to enable them to be repeated?	×		
Results				
12	Were the basic data adequately described?	×		
13	Does the response rate raise concerns about non-response bias?			N/A
14	If appropriate, was information about non-responders described?			N/A
15	Were the results internally consistent?	×		
16	Were the results presented for all the analyses described in the methods?	×		
Discussion				
17	Were the authors' discussions and conclusions justified by the results?	×		
18	Were the limitations of the study discussed?		×	
Other				
19	Were there any funding sources or conflicts of interest that may affect the authors’ interpretation of the results?		×	
20	Was ethical approval or consent of participants attained?	×		

Appraisal tool for Cross-Sectional Studies (AXIS) – Guedj et al. 2021 [40]

	Question	Yes	No	Don’t know/Comment
Introduction				
1	Were the aims/objectives of the study clear?	×		
Methods				
2	Was the study design appropriate for the stated aim(s)?	×		
3	Was the sample size justified?	×		
4	Was the target/reference population clearly defined? (Is it clear who the research was about?)	×		
5	Was the sample frame taken from an appropriate population base so that it closely represented the target/reference population under investigation?	×		

6	Was the selection process likely to select subjects/participants that were representative of the target/reference population under investigation?	×		
7	Were measures undertaken to address and categorise non-responders?			N/A
8	Were the risk factor and outcome variables measured appropriate to the aims of the study?	×		
9	Were the risk factor and outcome variables measured correctly using instruments/measurements that had been trialled, piloted or published previously?	×		
10	Is it clear what was used to determined statistical significance and/or precision estimates? (e.g. p-values, confidence intervals)	×		
11	Were the methods (including statistical methods) sufficiently described to enable them to be repeated?	×		
Results				
12	Were the basic data adequately described?	×		
13	Does the response rate raise concerns about non-response bias?			N/A
14	If appropriate, was information about non-responders described?			N/A
15	Were the results internally consistent?	×		
16	Were the results presented for all the analyses described in the methods?	×		
Discussion				
17	Were the authors' discussions and conclusions justified by the results?	×		
18	Were the limitations of the study discussed?	×		
Other				
19	Were there any funding sources or conflicts of interest that may affect the authors' interpretation of the results?		×	
20	Was ethical approval or consent of participants attained?	×		

Appraisal tool for Cross-Sectional Studies (AXIS) – Büttner et al. 2021 [41]

	Question	Yes	No	Don't know/Comment
Introduction				
1	Were the aims/objectives of the study clear?	×		
Methods				
2	Was the study design appropriate for the stated aim(s)?	×		
3	Was the sample size justified?	×		
4	Was the target/reference population clearly defined? (Is it clear who the research was about?)	×		
5	Was the sample frame taken from an appropriate population base so that it closely represented the target/reference population under investigation?	×		
6	Was the selection process likely to select subjects/participants that were representative of the target/reference population under investigation?	×		
7	Were measures undertaken to address and categorise non-responders?			N/A
8	Were the risk factor and outcome variables measured appropriate to the aims of the study?	×		
9	Were the risk factor and outcome variables measured correctly using instruments/measurements that had been trialled, piloted or published previously?	×		
10	Is it clear what was used to determined statistical significance and/or precision estimates? (e.g. p-values, confidence intervals)	×		
11	Were the methods (including statistical methods) sufficiently described to enable them to be repeated?	×		
Results				
12	Were the basic data adequately described?	×		
13	Does the response rate raise concerns about non-response bias?			N/A
14	If appropriate, was information about non-responders described?			N/A
15	Were the results internally consistent?	×		
16	Were the results presented for all the analyses described in the methods?	×		
Discussion				
17	Were the authors' discussions and conclusions justified by the results?	×		
18	Were the limitations of the study discussed?	×		
Other				
19	Were there any funding sources or conflicts of interest that may affect the authors' interpretation of the results?		×	
20	Was ethical approval or consent of participants attained?	×		

Appraisal tool for Cross-Sectional Studies (AXIS) – Thurnher et al. 2021 [42]

	Question	Yes	No	Don't know/Comment
Introduction				
1	Were the aims/objectives of the study clear?	×		
Methods				
2	Was the study design appropriate for the stated aim(s)?	×		
3	Was the sample size justified?	×		
4	Was the target/reference population clearly defined? (Is it clear who the research was about?)	×		
5	Was the sample frame taken from an appropriate population base so that it closely represented the target/reference population under investigation?	×		
6	Was the selection process likely to select subjects/participants that were representative of the target/reference population under investigation?	×		
7	Were measures undertaken to address and categorise non-responders?			N/A
8	Were the risk factor and outcome variables measured appropriate to the aims of the study?	×		
9	Were the risk factor and outcome variables measured correctly using instruments/measurements that had been trialled, piloted or published previously?	×		
10	Is it clear what was used to determined statistical significance and/or precision estimates? (e.g. p-values, confidence intervals)	×		
11	Were the methods (including statistical methods) sufficiently described to enable them to be repeated?	×		
Results				
12	Were the basic data adequately described?	×		
13	Does the response rate raise concerns about non-response bias?			N/A

14	If appropriate, was information about non-responders described?			N/A
15	Were the results internally consistent?	×		
16	Were the results presented for all the analyses described in the methods?	×		
Discussion				
17	Were the authors' discussions and conclusions justified by the results?	×		
18	Were the limitations of the study discussed?	×		
Other				
19	Were there any funding sources or conflicts of interest that may affect the authors' interpretation of the results?		×	
20	Was ethical approval or consent of participants attained?	×		

Appraisal tool for Cross-Sectional Studies (AXIS) – Agarwal et al. 2021 [43]

	Question	Yes	No	Don't know/Comment
Introduction				
1	Were the aims/objectives of the study clear?	×		
Methods				
2	Was the study design appropriate for the stated aim(s)?	×		
3	Was the sample size justified?	×		
4	Was the target/reference population clearly defined? (Is it clear who the research was about?)	×		
5	Was the sample frame taken from an appropriate population base so that it closely represented the target/reference population under investigation?	×		
6	Was the selection process likely to select subjects/participants that were representative of the target/reference population under investigation?	×		
7	Were measures undertaken to address and categorise non-responders?			N/A
8	Were the risk factor and outcome variables measured appropriate to the aims of the study?	×		
9	Were the risk factor and outcome variables measured correctly using instruments/measurements that had been trialled, piloted or published previously?	×		
10	Is it clear what was used to determined statistical significance and/or precision estimates? (e.g. p-values, confidence intervals)	×		
11	Were the methods (including statistical methods) sufficiently described to enable them to be repeated?	×		
Results				
12	Were the basic data adequately described?	×		
13	Does the response rate raise concerns about non-response bias?			N/A
14	If appropriate, was information about non-responders described?			N/A
15	Were the results internally consistent?	×		
16	Were the results presented for all the analyses described in the methods?	×		
Discussion				
17	Were the authors' discussions and conclusions justified by the results?	×		
18	Were the limitations of the study discussed?	×		
Other				
19	Were there any funding sources or conflicts of interest that may affect the authors' interpretation of the results?		×	
20	Was ethical approval or consent of participants attained?	×		

Appraisal tool for Cross-Sectional Studies (AXIS) – Mahammedi et al. 2021 [44]

	Question	Yes	No	Don't know/Comment
Introduction				
1	Were the aims/objectives of the study clear?	×		
Methods				
2	Was the study design appropriate for the stated aim(s)?	×		
3	Was the sample size justified?	×		
4	Was the target/reference population clearly defined? (Is it clear who the research was about?)	×		
5	Was the sample frame taken from an appropriate population base so that it closely represented the target/reference population under investigation?	×		
6	Was the selection process likely to select subjects/participants that were representative of the target/reference population under investigation?	×		
7	Were measures undertaken to address and categorise non-responders?			N/A
8	Were the risk factor and outcome variables measured appropriate to the aims of the study?	×		
9	Were the risk factor and outcome variables measured correctly using instruments/measurements that had been trialled, piloted or published previously?	×		
10	Is it clear what was used to determined statistical significance and/or precision estimates? (e.g. p-values, confidence intervals)	×		
11	Were the methods (including statistical methods) sufficiently described to enable them to be repeated?	×		
Results				
12	Were the basic data adequately described?	×		
13	Does the response rate raise concerns about non-response bias?			N/A
14	If appropriate, was information about non-responders described?			N/A
15	Were the results internally consistent?	×		
16	Were the results presented for all the analyses described in the methods?	×		
Discussion				
17	Were the authors' discussions and conclusions justified by the results?	×		
18	Were the limitations of the study discussed?	×		
Other				
19	Were there any funding sources or conflicts of interest that may affect the authors' interpretation of the results?		×	
20	Was ethical approval or consent of participants attained?	×		

Appraisal tool for Cross-Sectional Studies (AXIS) – Lambrecq et al. 2021 [45]

	Question	Yes	No	Don't know/Comment

Introduction				
1	Were the aims/objectives of the study clear?	×		
Methods				
2	Was the study design appropriate for the stated aim(s)?	×		
3	Was the sample size justified?	×		
4	Was the target/reference population clearly defined? (Is it clear who the research was about?)	×		
5	Was the sample frame taken from an appropriate population base so that it closely represented the target/reference population under investigation?	×		
6	Was the selection process likely to select subjects/participants that were representative of the target/reference population under investigation?	×		
7	Were measures undertaken to address and categorise non-responders?			N/A
8	Were the risk factor and outcome variables measured appropriate to the aims of the study?	×		
9	Were the risk factor and outcome variables measured correctly using instruments/measurements that had been trialled, piloted or published previously?	×		
10	Is it clear what was used to determined statistical significance and/or precision estimates? (e.g. p-values, confidence intervals)	×		
11	Were the methods (including statistical methods) sufficiently described to enable them to be repeated?	×		
Results				
12	Were the basic data adequately described?	×		
13	Does the response rate raise concerns about non-response bias?			N/A
14	If appropriate, was information about non-responders described?			N/A
15	Were the results internally consistent?	×		
16	Were the results presented for all the analyses described in the methods?	×		
Discussion				
17	Were the authors' discussions and conclusions justified by the results?	×		
18	Were the limitations of the study discussed?	×		
Other				
19	Were there any funding sources or conflicts of interest that may affect the authors' interpretation of the results?		×	
20	Was ethical approval or consent of participants attained?	×		

Appraisal tool for Cross-Sectional Studies (AXIS) – Yan et al. 2021 [46]

	Question	Yes	No	Don't know/Comment
Introduction				
1	Were the aims/objectives of the study clear?	×		
Methods				
2	Was the study design appropriate for the stated aim(s)?	×		
3	Was the sample size justified?		×	
4	Was the target/reference population clearly defined? (Is it clear who the research was about?)	×		
5	Was the sample frame taken from an appropriate population base so that it closely represented the target/reference population under investigation?	×		
6	Was the selection process likely to select subjects/participants that were representative of the target/reference population under investigation?	×		
7	Were measures undertaken to address and categorise non-responders?			N/A
8	Were the risk factor and outcome variables measured appropriate to the aims of the study?	×		
9	Were the risk factor and outcome variables measured correctly using instruments/measurements that had been trialled, piloted or published previously?	×		
10	Is it clear what was used to determined statistical significance and/or precision estimates? (e.g. p-values, confidence intervals)	×		
11	Were the methods (including statistical methods) sufficiently described to enable them to be repeated?	×		
Results				
12	Were the basic data adequately described?	×		
13	Does the response rate raise concerns about non-response bias?			N/A
14	If appropriate, was information about non-responders described?			N/A
15	Were the results internally consistent?	×		
16	Were the results presented for all the analyses described in the methods?	×		
Discussion				
17	Were the authors' discussions and conclusions justified by the results?	×		
18	Were the limitations of the study discussed?	×		
Other				
19	Were there any funding sources or conflicts of interest that may affect the authors' interpretation of the results?		×	
20	Was ethical approval or consent of participants attained?	×		

Appraisal tool for Cross-Sectional Studies (AXIS) – Uginet et al. 2022 [47]

	Question	Yes	No	Don't know/Comment
Introduction				
1	Were the aims/objectives of the study clear?	×		
Methods				
2	Was the study design appropriate for the stated aim(s)?	×		
3	Was the sample size justified?		×	
4	Was the target/reference population clearly defined? (Is it clear who the research was about?)	×		
5	Was the sample frame taken from an appropriate population base so that it closely represented the target/reference population under investigation?	×		
6	Was the selection process likely to select subjects/participants that were representative of the target/reference population under investigation?	×		
7	Were measures undertaken to address and categorise non-responders?			N/A
8	Were the risk factor and outcome variables measured appropriate to the aims of the study?	×		

9	Were the risk factor and outcome variables measured correctly using instruments/measurements that had been trialled, piloted or published previously?	×		
10	Is it clear what was used to determined statistical significance and/or precision estimates? (e.g. p-values, confidence intervals)	×		
11	Were the methods (including statistical methods) sufficiently described to enable them to be repeated?	×		
Results				
12	Were the basic data adequately described?	×		
13	Does the response rate raise concerns about non-response bias?			N/A
14	If appropriate, was information about non-responders described?			N/A
15	Were the results internally consistent?	×		
16	Were the results presented for all the analyses described in the methods?	×		
Discussion				
17	Were the authors' discussions and conclusions justified by the results?	×		
18	Were the limitations of the study discussed?	×		
Other				
19	Were there any funding sources or conflicts of interest that may affect the authors' interpretation of the results?		×	
20	Was ethical approval or consent of participants attained?	×		

Appraisal tool for Cross-Sectional Studies (AXIS) – Jegatheeswaran 2022 [48]

	Question	Yes	No	Don't know/Comment
Introduction				
1	Were the aims/objectives of the study clear?	×		
Methods				
2	Was the study design appropriate for the stated aim(s)?	×		
3	Was the sample size justified?	×		
4	Was the target/reference population clearly defined? (Is it clear who the research was about?)	×		
5	Was the sample frame taken from an appropriate population base so that it closely represented the target/reference population under investigation?	×		
6	Was the selection process likely to select subjects/participants that were representative of the target/reference population under investigation?	×		
7	Were measures undertaken to address and categorise non-responders?			N/A
8	Were the risk factor and outcome variables measured appropriate to the aims of the study?	×		
9	Were the risk factor and outcome variables measured correctly using instruments/measurements that had been trialled, piloted or published previously?	×		
10	Is it clear what was used to determined statistical significance and/or precision estimates? (e.g. p-values, confidence intervals)	×		
11	Were the methods (including statistical methods) sufficiently described to enable them to be repeated?	×		
Results				
12	Were the basic data adequately described?	×		
13	Does the response rate raise concerns about non-response bias?			N/A
14	If appropriate, was information about non-responders described?			N/A
15	Were the results internally consistent?	×		
16	Were the results presented for all the analyses described in the methods?	×		
Discussion				
17	Were the authors' discussions and conclusions justified by the results?	×		
18	Were the limitations of the study discussed?	×		
Other				
19	Were there any funding sources or conflicts of interest that may affect the authors' interpretation of the results?		×	
20	Was ethical approval or consent of participants attained?	×		

Appraisal tool for Cross-Sectional Studies (AXIS) – Tu et al. 2021 [49]

	Question	Yes	No	Don't know/Comment
Introduction				
1	Were the aims/objectives of the study clear?	×		
Methods				
2	Was the study design appropriate for the stated aim(s)?	×		
3	Was the sample size justified?	×		
4	Was the target/reference population clearly defined? (Is it clear who the research was about?)	×		
5	Was the sample frame taken from an appropriate population base so that it closely represented the target/reference population under investigation?	×		
6	Was the selection process likely to select subjects/participants that were representative of the target/reference population under investigation?	×		
7	Were measures undertaken to address and categorise non-responders?			N/A
8	Were the risk factor and outcome variables measured appropriate to the aims of the study?	×		
9	Were the risk factor and outcome variables measured correctly using instruments/measurements that had been trialled, piloted or published previously?	×		
10	Is it clear what was used to determined statistical significance and/or precision estimates? (e.g. p-values, confidence intervals)	×		
11	Were the methods (including statistical methods) sufficiently described to enable them to be repeated?	×		
Results				
12	Were the basic data adequately described?	×		
13	Does the response rate raise concerns about non-response bias?			N/A
14	If appropriate, was information about non-responders described?			N/A
15	Were the results internally consistent?	×		
16	Were the results presented for all the analyses described in the methods?	×		
Discussion				

17	Were the authors' discussions and conclusions justified by the results?	×		
18	Were the limitations of the study discussed?	×		
Other				
19	Were there any funding sources or conflicts of interest that may affect the authors’ interpretation of the results?		×	
20	Was ethical approval or consent of participants attained?	×		

Appraisal tool for Cross-Sectional Studies (AXIS) – Benedetti et al. 2021 [50]

	Question	Yes	No	Don’t know/Comment
Introduction				
1	Were the aims/objectives of the study clear?	×		
Methods				
2	Was the study design appropriate for the stated aim(s)?	×		
3	Was the sample size justified?	×		
4	Was the target/reference population clearly defined? (Is it clear who the research was about?)	×		
5	Was the sample frame taken from an appropriate population base so that it closely represented the target/reference population under investigation?	×		
6	Was the selection process likely to select subjects/participants that were representative of the target/reference population under investigation?	×		
7	Were measures undertaken to address and categorise non-responders?			N/A
8	Were the risk factor and outcome variables measured appropriate to the aims of the study?	×		
9	Were the risk factor and outcome variables measured correctly using instruments/measurements that had been trialled, piloted or published previously?	×		
10	Is it clear what was used to determined statistical significance and/or precision estimates? (e.g. p-values, confidence intervals)	×		
11	Were the methods (including statistical methods) sufficiently described to enable them to be repeated?	×		
Results				
12	Were the basic data adequately described?	×		
13	Does the response rate raise concerns about non-response bias?			N/A
14	If appropriate, was information about non-responders described?			N/A
15	Were the results internally consistent?	×		
16	Were the results presented for all the analyses described in the methods?	×		
Discussion				
17	Were the authors' discussions and conclusions justified by the results?	×		
18	Were the limitations of the study discussed?	×		
Other				
19	Were there any funding sources or conflicts of interest that may affect the authors’ interpretation of the results?		×	
20	Was ethical approval or consent of participants attained?	×		

Appraisal tool for Cross-Sectional Studies (AXIS) – Duarte et al. 2022 [51]

	Question	Yes	No	Don’t know/Comment
Introduction				
1	Were the aims/objectives of the study clear?	×		
Methods				
2	Was the study design appropriate for the stated aim(s)?	×		
3	Was the sample size justified?	×		
4	Was the target/reference population clearly defined? (Is it clear who the research was about?)	×		
5	Was the sample frame taken from an appropriate population base so that it closely represented the target/reference population under investigation?	×		
6	Was the selection process likely to select subjects/participants that were representative of the target/reference population under investigation?	×		
7	Were measures undertaken to address and categorise non-responders?			N/A
8	Were the risk factor and outcome variables measured appropriate to the aims of the study?	×		
9	Were the risk factor and outcome variables measured correctly using instruments/measurements that had been trialled, piloted or published previously?	×		
10	Is it clear what was used to determined statistical significance and/or precision estimates? (e.g. p-values, confidence intervals)	×		
11	Were the methods (including statistical methods) sufficiently described to enable them to be repeated?	×		
Results				
12	Were the basic data adequately described?	×		
13	Does the response rate raise concerns about non-response bias?			N/A
14	If appropriate, was information about non-responders described?			N/A
15	Were the results internally consistent?	×		
16	Were the results presented for all the analyses described in the methods?	×		
Discussion				
17	Were the authors' discussions and conclusions justified by the results?	×		
18	Were the limitations of the study discussed?		×	
Other				
19	Were there any funding sources or conflicts of interest that may affect the authors’ interpretation of the results?		×	
20	Was ethical approval or consent of participants attained?	×		

Appraisal tool for Cross-Sectional Studies (AXIS) – Nelson et al. 2022 [52]

	Question	Yes	No	Don’t know/Comment
Introduction				
1	Were the aims/objectives of the study clear?	×		
Methods				
2	Was the study design appropriate for the stated aim(s)?	×		

3	Was the sample size justified?		×	
4	Was the target/reference population clearly defined? (Is it clear who the research was about?)	×		
5	Was the sample frame taken from an appropriate population base so that it closely represented the target/reference population under investigation?	×		
6	Was the selection process likely to select subjects/participants that were representative of the target/reference population under investigation?	×		
7	Were measures undertaken to address and categorise non-responders?			N/A
8	Were the risk factor and outcome variables measured appropriate to the aims of the study?	×		
9	Were the risk factor and outcome variables measured correctly using instruments/measurements that had been trialled, piloted or published previously?	×		
10	Is it clear what was used to determined statistical significance and/or precision estimates? (e.g. p-values, confidence intervals)	×		
11	Were the methods (including statistical methods) sufficiently described to enable them to be repeated?	×		
Results				
12	Were the basic data adequately described?	×		
13	Does the response rate raise concerns about non-response bias?			N/A
14	If appropriate, was information about non-responders described?			N/A
15	Were the results internally consistent?	×		
16	Were the results presented for all the analyses described in the methods?	×		
Discussion				
17	Were the authors' discussions and conclusions justified by the results?	×		
18	Were the limitations of the study discussed?		×	
Other				
19	Were there any funding sources or conflicts of interest that may affect the authors' interpretation of the results?		×	
20	Was ethical approval or consent of participants attained?	×		

Appraisal tool for Cross-Sectional Studies (AXIS) – Andriuta et al. 2022 [53]

	Question	Yes	No	Don't know/Comment
Introduction				
1	Were the aims/objectives of the study clear?	×		
Methods				
2	Was the study design appropriate for the stated aim(s)?	×		
3	Was the sample size justified?	×		Retrospective male-only controls
4	Was the target/reference population clearly defined? (Is it clear who the research was about?)	×		
5	Was the sample frame taken from an appropriate population base so that it closely represented the target/reference population under investigation?	×		
6	Was the selection process likely to select subjects/participants that were representative of the target/reference population under investigation?	×		
7	Were measures undertaken to address and categorise non-responders?			N/A
8	Were the risk factor and outcome variables measured appropriate to the aims of the study?	×		
9	Were the risk factor and outcome variables measured correctly using instruments/measurements that had been trialled, piloted or published previously?	×		
10	Is it clear what was used to determined statistical significance and/or precision estimates? (e.g. p-values, confidence intervals)	×		
11	Were the methods (including statistical methods) sufficiently described to enable them to be repeated?	×		
Results				
12	Were the basic data adequately described?	×		
13	Does the response rate raise concerns about non-response bias?			N/A
14	If appropriate, was information about non-responders described?			N/A
15	Were the results internally consistent?	×		
16	Were the results presented for all the analyses described in the methods?	×		
Discussion				
17	Were the authors' discussions and conclusions justified by the results?	×		
18	Were the limitations of the study discussed?	×		
Other				
19	Were there any funding sources or conflicts of interest that may affect the authors' interpretation of the results?		×	
20	Was ethical approval or consent of participants attained?	×		

Appraisal tool for Cross-Sectional Studies (AXIS) – Widemon et al. 2022 [54]

	Question	Yes	No	Don't know/Comment
Introduction				
1	Were the aims/objectives of the study clear?	×		
Methods				
2	Was the study design appropriate for the stated aim(s)?	×		
3	Was the sample size justified?	×		
4	Was the target/reference population clearly defined? (Is it clear who the research was about?)	×		
5	Was the sample frame taken from an appropriate population base so that it closely represented the target/reference population under investigation?	×		
6	Was the selection process likely to select subjects/participants that were representative of the target/reference population under investigation?	×		
7	Were measures undertaken to address and categorise non-responders?			N/A
8	Were the risk factor and outcome variables measured appropriate to the aims of the study?	×		
9	Were the risk factor and outcome variables measured correctly using instruments/measurements that had been trialled, piloted or published previously?	×		

10	Is it clear what was used to determined statistical significance and/or precision estimates? (e.g. p-values, confidence intervals)	×		
11	Were the methods (including statistical methods) sufficiently described to enable them to be repeated?	×		
Results				
12	Were the basic data adequately described?	×		
13	Does the response rate raise concerns about non-response bias?			N/A
14	If appropriate, was information about non-responders described?			N/A
15	Were the results internally consistent?	×		
16	Were the results presented for all the analyses described in the methods?	×		
Discussion				
17	Were the authors' discussions and conclusions justified by the results?	×		
18	Were the limitations of the study discussed?	×		
Other				
19	Were there any funding sources or conflicts of interest that may affect the authors' interpretation of the results?		×	
20	Was ethical approval or consent of participants attained?			×, exemption obtained

Appraisal tool for Cross-Sectional Studies (AXIS) – Lersy et al. 2022 [55]

	Question	Yes	No	Don't know/Comment
Introduction				
1	Were the aims/objectives of the study clear?	×		
Methods				
2	Was the study design appropriate for the stated aim(s)?	×		
3	Was the sample size justified?	×		
4	Was the target/reference population clearly defined? (Is it clear who the research was about?)	×		
5	Was the sample frame taken from an appropriate population base so that it closely represented the target/reference population under investigation?	×		
6	Was the selection process likely to select subjects/participants that were representative of the target/reference population under investigation?	×		
7	Were measures undertaken to address and categorise non-responders?			N/A
8	Were the risk factor and outcome variables measured appropriate to the aims of the study?	×		
9	Were the risk factor and outcome variables measured correctly using instruments/measurements that had been trialled, piloted or published previously?	×		
10	Is it clear what was used to determined statistical significance and/or precision estimates? (e.g. p-values, confidence intervals)	×		
11	Were the methods (including statistical methods) sufficiently described to enable them to be repeated?	×		
Results				
12	Were the basic data adequately described?	×		
13	Does the response rate raise concerns about non-response bias?			N/A
14	If appropriate, was information about non-responders described?			N/A
15	Were the results internally consistent?	×		
16	Were the results presented for all the analyses described in the methods?	×		
Discussion				
17	Were the authors' discussions and conclusions justified by the results?	×		
18	Were the limitations of the study discussed?	×		
Other				
19	Were there any funding sources or conflicts of interest that may affect the authors' interpretation of the results?		×	
20	Was ethical approval or consent of participants attained?	×		

Appraisal tool for Cross-Sectional Studies (AXIS) – de Paula et al. 2023 [56]

	Question	Yes	No	Don't know/Comment
Introduction				
1	Were the aims/objectives of the study clear?	×		
Methods				
2	Was the study design appropriate for the stated aim(s)?	×		
3	Was the sample size justified?	×		
4	Was the target/reference population clearly defined? (Is it clear who the research was about?)	×		
5	Was the sample frame taken from an appropriate population base so that it closely represented the target/reference population under investigation?	×		
6	Was the selection process likely to select subjects/participants that were representative of the target/reference population under investigation?	×		
7	Were measures undertaken to address and categorise non-responders?			N/A
8	Were the risk factor and outcome variables measured appropriate to the aims of the study?	×		
9	Were the risk factor and outcome variables measured correctly using instruments/measurements that had been trialled, piloted or published previously?	×		
10	Is it clear what was used to determined statistical significance and/or precision estimates? (e.g. p-values, confidence intervals)	×		
11	Were the methods (including statistical methods) sufficiently described to enable them to be repeated?	×		
Results				
12	Were the basic data adequately described?	×		
13	Does the response rate raise concerns about non-response bias?			N/A
14	If appropriate, was information about non-responders described?			N/A
15	Were the results internally consistent?	×		
16	Were the results presented for all the analyses described in the methods?	×		
Discussion				
17	Were the authors' discussions and conclusions justified by the results?	×		

18	Were the limitations of the study discussed?			× Possible selection bias recognized
Other				
19	Were there any funding sources or conflicts of interest that may affect the authors' interpretation of the results?		×	
20	Was ethical approval or consent of participants attained?	×		

Appraisal tool for Cross-Sectional Studies (AXIS) – Callen et al. 2023 [57]

	Question	Yes	No	Don't know/Comment
Introduction				
1	Were the aims/objectives of the study clear?	×		
Methods				
2	Was the study design appropriate for the stated aim(s)?	×		
3	Was the sample size justified?		×	
4	Was the target/reference population clearly defined? (Is it clear who the research was about?)	×		
5	Was the sample frame taken from an appropriate population base so that it closely represented the target/reference population under investigation?	×		
6	Was the selection process likely to select subjects/participants that were representative of the target/reference population under investigation?	×		
7	Were measures undertaken to address and categorise non-responders?			N/A
8	Were the risk factor and outcome variables measured appropriate to the aims of the study?	×		
9	Were the risk factor and outcome variables measured correctly using instruments/measurements that had been trialled, piloted or published previously?	×		
10	Is it clear what was used to determined statistical significance and/or precision estimates? (e.g. p-values, confidence intervals)	×		
11	Were the methods (including statistical methods) sufficiently described to enable them to be repeated?	×		
Results				
12	Were the basic data adequately described?	×		
13	Does the response rate raise concerns about non-response bias?			N/A
14	If appropriate, was information about non-responders described?			N/A
15	Were the results internally consistent?	×		
16	Were the results presented for all the analyses described in the methods?	×		
Discussion				
17	Were the authors' discussions and conclusions justified by the results?	×		
18	Were the limitations of the study discussed?	×		
Other				
19	Were there any funding sources or conflicts of interest that may affect the authors' interpretation of the results?		×	
20	Was ethical approval or consent of participants attained?	×		

Appraisal tool for Cross-Sectional Studies (AXIS) – Díez-Cirarda et al. 2022 [58]

	Question	Yes	No	Don't know/Comment
Introduction				
1	Were the aims/objectives of the study clear?	×		
Methods				
2	Was the study design appropriate for the stated aim(s)?	×		
3	Was the sample size justified?	×		
4	Was the target/reference population clearly defined? (Is it clear who the research was about?)	×		
5	Was the sample frame taken from an appropriate population base so that it closely represented the target/reference population under investigation?	×		
6	Was the selection process likely to select subjects/participants that were representative of the target/reference population under investigation?	×		
7	Were measures undertaken to address and categorise non-responders?			N/A
8	Were the risk factor and outcome variables measured appropriate to the aims of the study?	×		
9	Were the risk factor and outcome variables measured correctly using instruments/measurements that had been trialled, piloted or published previously?	×		
10	Is it clear what was used to determined statistical significance and/or precision estimates? (e.g. p-values, confidence intervals)	×		
11	Were the methods (including statistical methods) sufficiently described to enable them to be repeated?	×		
Results				
12	Were the basic data adequately described?	×		
13	Does the response rate raise concerns about non-response bias?			N/A
14	If appropriate, was information about non-responders described?			N/A
15	Were the results internally consistent?	×		
16	Were the results presented for all the analyses described in the methods?	×		
Discussion				
17	Were the authors' discussions and conclusions justified by the results?	×		
18	Were the limitations of the study discussed?	×		
Other				
19	Were there any funding sources or conflicts of interest that may affect the authors' interpretation of the results?		×	
20	Was ethical approval or consent of participants attained?	×		

Appraisal tool for Cross-Sectional Studies (AXIS) – Goehringer et al. 2023 [59]

	Question	Yes	No	Don't know/Comment
Introduction				
1	Were the aims/objectives of the study clear?	×		
Methods				
2	Was the study design appropriate for the stated aim(s)?	×		
3	Was the sample size justified?		×	

4	Was the target/reference population clearly defined? (Is it clear who the research was about?)	×		
5	Was the sample frame taken from an appropriate population base so that it closely represented the target/reference population under investigation?	×		
6	Was the selection process likely to select subjects/participants that were representative of the target/reference population under investigation?	×		
7	Were measures undertaken to address and categorise non-responders?			N/A
8	Were the risk factor and outcome variables measured appropriate to the aims of the study?	×		
9	Were the risk factor and outcome variables measured correctly using instruments/measurements that had been trialled, piloted or published previously?	×		
10	Is it clear what was used to determined statistical significance and/or precision estimates? (e.g. p-values, confidence intervals)	×		
11	Were the methods (including statistical methods) sufficiently described to enable them to be repeated?	×		
Results				
12	Were the basic data adequately described?	×		
13	Does the response rate raise concerns about non-response bias?			N/A
14	If appropriate, was information about non-responders described?			N/A
15	Were the results internally consistent?	×		
16	Were the results presented for all the analyses described in the methods?	×		
Discussion				
17	Were the authors' discussions and conclusions justified by the results?	×		
18	Were the limitations of the study discussed?		×	
Other				
19	Were there any funding sources or conflicts of interest that may affect the authors' interpretation of the results?		×	
20	Was ethical approval or consent of participants attained?	×		

Appraisal tool for Cross-Sectional Studies (AXIS) – Paolini et al. 2023 [60]

	Question	Yes	No	Don't know/Comment
Introduction				
1	Were the aims/objectives of the study clear?	×		
Methods				
2	Was the study design appropriate for the stated aim(s)?	×		
3	Was the sample size justified?	×		
4	Was the target/reference population clearly defined? (Is it clear who the research was about?)	×		
5	Was the sample frame taken from an appropriate population base so that it closely represented the target/reference population under investigation?	×		
6	Was the selection process likely to select subjects/participants that were representative of the target/reference population under investigation?	×		
7	Were measures undertaken to address and categorise non-responders?			N/A
8	Were the risk factor and outcome variables measured appropriate to the aims of the study?	×		
9	Were the risk factor and outcome variables measured correctly using instruments/measurements that had been trialled, piloted or published previously?	×		
10	Is it clear what was used to determined statistical significance and/or precision estimates? (e.g. p-values, confidence intervals)	×		
11	Were the methods (including statistical methods) sufficiently described to enable them to be repeated?	×		
Results				
12	Were the basic data adequately described?	×		
13	Does the response rate raise concerns about non-response bias?			N/A
14	If appropriate, was information about non-responders described?			N/A
15	Were the results internally consistent?	×		
16	Were the results presented for all the analyses described in the methods?	×		
Discussion				
17	Were the authors' discussions and conclusions justified by the results?	×		
18	Were the limitations of the study discussed?	×		
Other				
19	Were there any funding sources or conflicts of interest that may affect the authors' interpretation of the results?		×	
20	Was ethical approval or consent of participants attained?	×		

Appraisal tool for Cross-Sectional Studies (AXIS) – Kamasak et al. 2023 [61]

	Question	Yes	No	Don't know/Comment
Introduction				
1	Were the aims/objectives of the study clear?	×		
Methods				
2	Was the study design appropriate for the stated aim(s)?	×		
3	Was the sample size justified?		×	
4	Was the target/reference population clearly defined? (Is it clear who the research was about?)	×		
5	Was the sample frame taken from an appropriate population base so that it closely represented the target/reference population under investigation?	×		
6	Was the selection process likely to select subjects/participants that were representative of the target/reference population under investigation?	×		
7	Were measures undertaken to address and categorise non-responders?			N/A
8	Were the risk factor and outcome variables measured appropriate to the aims of the study?	×		
9	Were the risk factor and outcome variables measured correctly using instruments/measurements that had been trialled, piloted or published previously?	×		
10	Is it clear what was used to determined statistical significance and/or precision estimates? (e.g. p-values, confidence intervals)	×		
11	Were the methods (including statistical methods) sufficiently described to enable them to be repeated?	×		

Results				
12	Were the basic data adequately described?	×		
13	Does the response rate raise concerns about non-response bias?			N/A
14	If appropriate, was information about non-responders described?			N/A
15	Were the results internally consistent?	×		
16	Were the results presented for all the analyses described in the methods?	×		
Discussion				
17	Were the authors' discussions and conclusions justified by the results?	×		
18	Were the limitations of the study discussed?		×	
Other				
19	Were there any funding sources or conflicts of interest that may affect the authors' interpretation of the results?		×	
20	Was ethical approval or consent of participants attained?	×		

Appraisal tool for Cross-Sectional Studies (AXIS) – Klinkhammer et al. 2023 [62]

	Question	Yes	No	Don't know/Comment
Introduction				
1	Were the aims/objectives of the study clear?	×		
Methods				
2	Was the study design appropriate for the stated aim(s)?	×		
3	Was the sample size justified?	×		
4	Was the target/reference population clearly defined? (Is it clear who the research was about?)	×		
5	Was the sample frame taken from an appropriate population base so that it closely represented the target/reference population under investigation?	×		
6	Was the selection process likely to select subjects/participants that were representative of the target/reference population under investigation?	×		
7	Were measures undertaken to address and categorise non-responders?			N/A
8	Were the risk factor and outcome variables measured appropriate to the aims of the study?	×		
9	Were the risk factor and outcome variables measured correctly using instruments/measurements that had been trialled, piloted or published previously?	×		
10	Is it clear what was used to determined statistical significance and/or precision estimates? (e.g. p-values, confidence intervals)	×		
11	Were the methods (including statistical methods) sufficiently described to enable them to be repeated?	×		
Results				
12	Were the basic data adequately described?	×		
13	Does the response rate raise concerns about non-response bias?			N/A
14	If appropriate, was information about non-responders described?			N/A
15	Were the results internally consistent?	×		
16	Were the results presented for all the analyses described in the methods?	×		
Discussion				
17	Were the authors' discussions and conclusions justified by the results?	×		
18	Were the limitations of the study discussed?	×		
Other				
19	Were there any funding sources or conflicts of interest that may affect the authors' interpretation of the results?		×	
20	Was ethical approval or consent of participants attained?	×		

Appraisal tool for Cross-Sectional Studies (AXIS) – Douaud et al. 2022 [63]

	Question	Yes	No	Don't know/Comment
Introduction				
1	Were the aims/objectives of the study clear?	×		
Methods				
2	Was the study design appropriate for the stated aim(s)?	×		
3	Was the sample size justified?	×		
4	Was the target/reference population clearly defined? (Is it clear who the research was about?)	×		
5	Was the sample frame taken from an appropriate population base so that it closely represented the target/reference population under investigation?	×		
6	Was the selection process likely to select subjects/participants that were representative of the target/reference population under investigation?	×		
7	Were measures undertaken to address and categorise non-responders?			N/A
8	Were the risk factor and outcome variables measured appropriate to the aims of the study?	×		
9	Were the risk factor and outcome variables measured correctly using instruments/measurements that had been trialled, piloted or published previously?	×		
10	Is it clear what was used to determined statistical significance and/or precision estimates? (e.g. p-values, confidence intervals)	×		
11	Were the methods (including statistical methods) sufficiently described to enable them to be repeated?	×		
Results				
12	Were the basic data adequately described?	×		
13	Does the response rate raise concerns about non-response bias?			N/A
14	If appropriate, was information about non-responders described?			N/A
15	Were the results internally consistent?	×		
16	Were the results presented for all the analyses described in the methods?	×		
Discussion				
17	Were the authors' discussions and conclusions justified by the results?	×		
18	Were the limitations of the study discussed?	×		
Other				
19	Were there any funding sources or conflicts of interest that may affect the authors' interpretation of the results?		×	

20	Was ethical approval or consent of participants attained?	×		

Appraisal tool for Cross-Sectional Studies (AXIS) – Burulday et al. 2023 [64]

	Question	Yes	No	Don't know/Comment
Introduction				
1	Were the aims/objectives of the study clear?	×		
Methods				
2	Was the study design appropriate for the stated aim(s)?	×		
3	Was the sample size justified?	×		
4	Was the target/reference population clearly defined? (Is it clear who the research was about?)	×		
5	Was the sample frame taken from an appropriate population base so that it closely represented the target/reference population under investigation?	×		
6	Was the selection process likely to select subjects/participants that were representative of the target/reference population under investigation?	×		
7	Were measures undertaken to address and categorise non-responders?			N/A
8	Were the risk factor and outcome variables measured appropriate to the aims of the study?	×		
9	Were the risk factor and outcome variables measured correctly using instruments/measurements that had been trialled, piloted or published previously?	×		
10	Is it clear what was used to determined statistical significance and/or precision estimates? (e.g. p-values, confidence intervals)	×		
11	Were the methods (including statistical methods) sufficiently described to enable them to be repeated?	×		
Results				
12	Were the basic data adequately described?	×		
13	Does the response rate raise concerns about non-response bias?			N/A
14	If appropriate, was information about non-responders described?			N/A
15	Were the results internally consistent?	×		
16	Were the results presented for all the analyses described in the methods?	×		
Discussion				
17	Were the authors' discussions and conclusions justified by the results?	×		
18	Were the limitations of the study discussed?		×	
Other				
19	Were there any funding sources or conflicts of interest that may affect the authors' interpretation of the results?		×	
20	Was ethical approval or consent of participants attained?	×		

Appraisal tool for Cross-Sectional Studies (AXIS) – Debs et al. 2023 [65]

	Question	Yes	No	Don't know/Comment
Introduction				
1	Were the aims/objectives of the study clear?	×		
Methods				
2	Was the study design appropriate for the stated aim(s)?	×		
3	Was the sample size justified?	×		
4	Was the target/reference population clearly defined? (Is it clear who the research was about?)	×		
5	Was the sample frame taken from an appropriate population base so that it closely represented the target/reference population under investigation?	×		
6	Was the selection process likely to select subjects/participants that were representative of the target/reference population under investigation?	×		
7	Were measures undertaken to address and categorise non-responders?			N/A
8	Were the risk factor and outcome variables measured appropriate to the aims of the study?	×		
9	Were the risk factor and outcome variables measured correctly using instruments/measurements that had been trialled, piloted or published previously?	×		
10	Is it clear what was used to determined statistical significance and/or precision estimates? (e.g. p-values, confidence intervals)	×		
11	Were the methods (including statistical methods) sufficiently described to enable them to be repeated?	×		
Results				
12	Were the basic data adequately described?	×		
13	Does the response rate raise concerns about non-response bias?			N/A
14	If appropriate, was information about non-responders described?			N/A
15	Were the results internally consistent?	×		
16	Were the results presented for all the analyses described in the methods?	×		
Discussion				
17	Were the authors' discussions and conclusions justified by the results?	×		
18	Were the limitations of the study discussed?	×		
Other				
19	Were there any funding sources or conflicts of interest that may affect the authors' interpretation of the results?		×	
20	Was ethical approval or consent of participants attained?	×		

Supplementary Table S4. Locations of the eligible studies.

Location	Study
11 centers, France	Kremer, S.; Lersy, F.; Anheim, M.; Merdji, H.; Schenck, M.; Oesterlé, H.; Bolognini, F.; Messie, J.; Khalil, A.; Gaudemer, A.; Carré, S.; Alleg, M.; Lecocq, C.; Schmitt, E.; Anxionnat, R.; Zhu, F.; Jager, L.; Nesser, P.; Mba, Y.T.; Hmeydia, G.; Benzakoun, J.; Oppenheim, C.; Ferré, J.C.; Maamar, A.; Carsin-Nicol, B.; Comby, P.O.; Ricolfi, F.; Thouant, P.; Boutet, C.; Fabre, X.; Forestier, G.; de Beaurepaire, I.; Bornet, G.; Desal, H.; Boulouis, G.; Berge, J.; Kazémi, A.; Pyatigorskaya, N.; Lecler, A.; Saleme, S.; Edjlali-Goujon, M.; Kerleroux, B.; Constans, J.M.; Zorn, P.E.; Mathieu, M.; Baloglu, S.; Ardellier, F.D.; Willaume, T.; Brisset, J.C.; Caillard, S.; Collange, O.; Mertes, P.M.; Schneider, F.; Fafi-Kremer, S.; Ohana, M.; Meziani, F.; Meyer, N.; Helms, J.; Cotton, F. Neurologic and neuroimaging findings in patients with COVID-

	19: A retrospective multicenter study. *Neurology* **2020**, *95*(13), e1868-e1882. doi: 10.1212/WNL.0000000000010112. Epub 2020 Jul 17.
2 centers, Stockholm, Sweden	Klironomos, S.; Tzortzakakis, A.; Kits, A.; Öhberg, C.; Kollia, E.; Ahoromazdae, A.; Almqvist, H.; Aspelin, Å.; Martin, H.; Ouellette, R.; Al-Saadi, J.; Hasselberg, M.; Haghgou, M.; Pedersen, M.; Petersson, S.; Finnsson, J.; Lundberg, J.; Falk Delgado, A.; Granberg, T. Nervous system involvement in Coronavirus Disease 2019: Results from a retrospective consecutive neuroimaging cohort. *Radiology* **2020**, *297*(3):E324-E334. doi: 10.1148/radiol.2020202791. Epub 2020 Jul 30.
1 center, MGH, Boston, Mass., USA	O'Shea, A.; Parakh, A.; Hedgire, S.; Lee, SI. Multisystem assessment of the imaging manifestations of coagulopathy in hospitalized patients with coronavirus disease (COVID-19). *AJR Am. J. Roentgenol.* **2021**, *216*(4), 1088-1098. doi: 10.2214/AJR.20.24132. Epub 2021 Feb 3.
2 centers, New York City, NY, USA	Lin, E.; Lantos, J.E.; Strauss, S.B.; Phillips, C.D.; Campion, T.R, Jr.; Navi, B.B.; Parikh, N.S.; Merkler, A.E.; Mir, S.; Zhang, C.; Kamel, H.; Cusick, M.; Goyal, P.; Gupta, A. Brain imaging of patients with COVID-19: Findings at an academic institution during the height of the outbreak in New York City. *AJNR Am. J. Neuroradiol.* **2020**, *41*(11), 2001-2008. doi: 10.3174/ajnr.A6793. Epub 2020 Aug 20.
1 center, Birmingham, UK	Sawlani, V.; Scotton, S.; Nader, K.; Jen, J.P.; Patel, M.; Gokani, K.; Denno, P.; Thaller, M.; Englezou, C.; Janjua, U.; Bowen, M.; Hoskote, C.; Veenith, T.; Hassan-Smith, G.; Jacob, S. COVID-19-related intracranial imaging findings: a large single-centre experience. *Clin. Radiol.* **2021**, *76*(2), 108-116. doi: 10.1016/j.crad.2020.09.002. Epub 2020 Sep 15.
International call by the American Society of Pediatric Neuroradiology, 10 countries	Lindan, C.E.; Mankad, K.; Ram, D.; Kociolek, L.K.; Silvera, V.M.; Boddaert, N.; Stivaros, S.M.; Palasis, S; ASPNR PECOBIG Collaborator Group. Neuroimaging manifestations in children with SARS-CoV-2 infection: a multinational, multicentre collaborative study. *Lancet Child Adolesc. Health* **2021**, *5*(3), 167-177. doi: 10.1016/S2352-4642(20)30362-X. Epub 2020 Dec 16.
1 center, Houston, Tx, USA	Orman, G.; Desai, N.K.; Kralik, S.F.; Meoded, A.; Seghers, V.J.; Annapragada, A.V.; Huisman, T.A.G.M. Neuroimaging offers low yield in children positive for SARS-CoV-2. *AJNR Am. J. Neuroradiol.* **2021**, *42*(5), 951-954. doi: 10.3174/ajnr.A7022. Epub 2021 Jan 7.
1 center, MGH, Boston, Mass., USA	Rapalino, O.; Pourvaziri, A.; Maher, M.; Jaramillo-Cardoso, A.; Edlow, B.L.; Conklin, J.; Huang, S.; Westover, B.; Romero, J.M.; Halpern, E.; Gupta, R.; Pomerantz, S.; Schaefer, P.; Gonzalez, R.G.; Mukerji, S.S.; Lev, M.H. Clinical, imaging, and lab correlates of severe COVID-19 leukoencephalopathy. *AJNR Am. J. Neuroradiol.* **2021**, *42*(4), 632-638. doi: 10.3174/ajnr.A6966. Epub 2021 Jan 7.
1 center, Pitié-Salpêtrière, Paris, France	Kas, A.; Soret, M.; Pyatigoskaya, N.; Habert, M.O.; Hesters, A.; Le Guennec, L.; Paccoud, O.; Bombois, S.; Delorme, C; on the behalf of CoCo-Neurosciences study group and COVID SMIT PSL study group. The cerebral network of COVID-19-related encephalopathy: a longitudinal voxel-based ^{18}F-FDG-PET study. *Eur. J. Nucl. Med. Mol. Imaging* **2021**, *48*(8), 2543-2557. doi: 10.1007/s00259-020-05178-y. Epub 2021 Jan 15.
1 center, MGH, Boston, Mass., USA	Conklin, J.; Frosch, M.P.; Mukerji, S.S.; Rapalino, O.; Maher, M.D.; Schaefer, P.W.; Lev, M.H.; Gonzalez, R.G.; Das, S.; Champion, S.N.; Magdamo, C.; Sen, P.; Harrold, G.K.; Alabsi, H.; Normandin, E.; Shaw, B.; Lemieux, J.E.; Sabeti, P.C.; Branda, J.A.; Brown, E.N.; Westover, M.B.; Huang, S.Y.; Edlow, B.L. Susceptibility-weighted imaging reveals cerebral microvascular injury in severe COVID-19. *J. Neurol. Sci.* **2021**, *421*, 117308. doi: 10.1016/j.jns.2021.117308. Epub 2021 Jan 15.
1 center, Marseille, France	Guedj, E.; Campion, J.Y.; Dudouet, P.; Kaphan, E.; Bregeon, F.; Tissot-Dupont, H.; Guis, S.; Barthelemy, F.; Habert, P.; Ceccaldi, M.; Million, M.; Raoult, D.; Cammilleri, S.; Eldin, C. ^{18}F-FDG brain PET hypometabolism in patients with long COVID. *Eur. J. Nucl. Med. Mol. Imaging* **2021**, *48*(9), 2823-2833. doi: 10.1007/s00259-021-05215-4. Epub 2021 Jan 26.
1 center, Homboldt-Charité, Berlin, Germany	Büttner, L.; Bauknecht, H.C.; Fleckenstein, F.N.; Kahn, J.; Tietze, A.; Bohner, G.; Siebert, E. Neuroimaging findings in conjunction with severe COVID-19. *Rofo* **2021**, *193*(7), 822-829. doi: 10.1055/a-1345-9784. Epub 2021 Feb 3.
1 center, Vienna, Austria	Thurnher, M.M.; Boban, J.; Röggla, M.; Staudinger, T. Distinct pattern of microsusceptibility changes on brain magnetic resonance imaging (MRI) in critically ill patients on mechanical ventilation/oxygenation. *Neuroradiology* **2021**, *63*(10), 1651-1658. doi: 10.1007/s00234-021-02663-5. Epub 2021 Mar 1.
3-center, New York City, NY, USA	Agarwal, S.; Melmed, K.; Dogra, S.; Jain, R.; Conway, J.; Galetta, S.; Lewis, A. Increase in ventricle size and the evolution of white matter changes on serial imaging in critically ill patients with COVID-19. *Neurocrit. Care* **2021**, *35*(2), 491-500. doi: 10.1007/s12028-021-01207-2. Epub 2021 Mar 5.
5-center, University of Cincinnati Medical Center, Cincinnati, Ohio, USA, Hospital 12 de Octubre, Madrid, Spain; Hospital Clínic, Barcelona, Spain; University of Cagliari, Cagliari, Italy; and Federal University of Sao Paulo, Sao Paulo, Brazil	Mahammedi, A.; Ramos, A.; Bargalló, N.; Gaskill, M.; Kapur, S.; Saba, L.; Carrete, H, Jr.; Sengupta, S.; Salvador, E.; Hilario, A.; Revilla, Y.; Sanchez, M.; Perez-Nuñez, M.; Bachir, S.; Zhang, B.; Oleaga, L.; Sergio, J.; Koren, L.; Martin-Medina, P.; Wang, L.; Benegas, M.; Ostos, F.; Gonzalez-Ortega, G.; Calleja, P.; Udstuen, G.; Williamson, B.; Khandwala, V.; Chadalavada, S.; Woo, D.; Vagal, A. Brain and lung imaging correlation in patients with COVID-19: Could the severity of lung disease reflect the prevalence of acute abnormalities on neuroimaging? A global multicenter observational study. *AJNR Am. J. Neuroradiol.* **2021**, *42*(6), 1008-1016. doi: 10.3174/ajnr.A7072. Epub 2021 Mar 11.
1 center, Pitié-Salpêtrière, Paris, France	Lambrecq, V.; Hanin, A.; Munoz-Musat, E.; Chougar, L.; Gassama, S.; Delorme, C.; Cousyn, L.; Borden, A.; Damiano, M.; Frazzini, V.; Huberfeld, G.; Landgraf, F.; Nguyen-Michel, V.H.; Pichit, P.; Sangare, A.; Chavez, M.; Morélot-Panzini, C.; Morawiec, E.; Raux, M.; Luyt, C.E.; Rufat, P.; Galanaud, D.; Corvol, J.C.; Lubetzki, C.; Rohaut, B.; Demeret, S.; Pyatigorskaya, N.; Naccache, L.; Navarro, V; Cohort COVID-19 Neurosciences (CoCo Neurosciences) Study Group. Association of clinical, biological, and brain magnetic resonance imaging findings with electroencephalographic findings for patients with COVID-19. *JAMA Netw. Open* **2021**, *4*(3), e211489. doi: 10.1001/jamanetworkopen.2021.1489.
4-center, 2 Shanghai, 1 Chengdu, 1 Wuhan, China	Yan, K.; Xiao, F.-F.; Jiang, Y.-W.; Xiao, T.-T.; Zhang, D.-J.; Yuan, W.-H.; Shao, J.-B.; Cheng, G.-Q.; Zeng, L.-K. Effects of SARS-CoV-2 infection on neuroimaging and neurobehavior in neonates. *World J. Pediatr.* **2021**, *17*(2), 171-179. doi: 10.1007/s12519-021-00423-2. Epub 2021 Mar 19.
1-center, Geneva, Switzerland	Uginet, M.; Breville, G.; Hofmeister, J.; Machi, P.; Lalive, P.H.; Rosi, A.; Fitsiori, A.; Vargas, M.I.; Assal, F.; Allali, G.; Lovblad, K.O. Cerebrovascular complications and vessel wall imaging in COVID-19 encephalopathy-A pilot study. *Clin. Neuroradiol.* **2022**, *32*(1), 287-293. doi: 10.1007/s00062-021-01008-2. Epub 2021 Mar 26.
3-center, Ontario, Canada	Jegatheeswaran, V.; Chan, M.W.K.; Chakrabarti, S.; Fawcett, A.; Chen, Y.A. Neuroimaging findings of hospitalized Covid-19 patients: A Canadian retrospective observational study. *Can. Assoc. Radiol. J.* **2022**, *73*(1), 179-186. doi: 10.1177/08465371211002815. Epub 2021 Apr 21.
2-center, Beijing, Shanghai, China	Tu, Y.; Zhang, Y.; Li, Y.; Zhao, Q.; Bi, Y.; Lu, X.; Kong, Y.; Wang, L.; Lu, Z.; Hu, L. Post-traumatic stress symptoms in COVID-19 survivors: a self-report and brain imaging follow-up study. *Mol. Psychiatry* **2021**, *26*(12), 7475-7480. doi: 10.1038/s41380-021-01223-w. Epub 2021 Jul 20.
1-center, San Raffaele, Milan, Italy	Benedetti, F.; Palladini, M.; Paolini, M.; Melloni, E.; Vai, B.; De, Lorenzo, R.; Furlan, R.; Rovere-Querini, P.; Falini, A.; Mazza, MG. Brain correlates of depression, post-traumatic distress, and inflammatory biomarkers in COVID-19 survivors: A multimodal magnetic resonance imaging study. *Brain Behav. Immun. Health* **2021**, *18*, 100387. doi: 10.1016/j.bbih.2021.100387. Epub 2021 Nov 2.

2-center, São Paulo, SP, and Serra, Espírito Santo, Brazil	Duarte AC, Fujiki RHM, Glória LFP, Fragoso DC, Pacheco FT, Calvi C, Rosa-Júnior M, Maia Junior ACM, Rocha AJD. Neurological imaging findings in hospitalized COVID-19 patients: a retrospective observational study in two Brazilian reference centers. *Arq. Neuropsiquiatr.* **2022**, *80*(5), 490-496. doi: 10.1590/0004-282X-ANP-2021-0103.
1-center, Karolinska Institutet, Stockholm, Sweden	Nelson DW, Granberg T, Andersen P, Jokhadar E, Kåhlin J, Granström A, Hallinder H, Schening A, Thunborg C, Walles H, Hagman G, Shams-Latifi R, Yu J, Petersson S, Tzortzakakis A, Levak N, Aspö M, Piehl F, Zetterberg H, Kivipelto M, Eriksson LI. The Karolinska NeuroCOVID study protocol: Neurocognitive impairment, biomarkers and advanced imaging in critical care survivors. Acta Anaesthesiol Scand. 2022;66(6):759-766. doi: 10.1111/aas.14062. Epub 2022 Apr 2.
1-center, Amiens, France	Andriuta D, Si-Ahmed C, Roussel M, Constans JM, Makki M, Aarabi A, Basille D, Andrejak C, Godefroy O. Clinical and imaging determinants of neurocognitive disorders in post-acute COVID-19 patients with cognitive complaints. J Alzheimers Dis. 2022;87(3):1239-1250. doi: 10.3233/JAD-215506.
1-center, Columbia University Irving Medical Center, New York, NY, USA	Widemon RS, Huang S, Capaccione KM, Mitchell RP, Salvatore MM, Lignelli A, Nguyen P. The impact of the COVID-19 pandemic on neuroimaging volume in New York City. Neuroradiol J. 2022;35(6):713-717. doi: 10.1177/19714009221096828. Epub 2022 May 3.
1-Center, Strasbourg, France	Lersy, F.; Bund, C.; Anheim, M.; Mondino, M.; Noblet, V.; Lazzara, S.; Phillipps, C.; Collange, O.; Oulehri, W.; Mertes, P.M.; Helms, J.; Merdji, H.; Schenck, M.; Schneider, F.; Pottecher, J.; Giraudeau, C.; Chammas, A.; Ardellier, F.D.; Baloglu, S.; Ambarki, K.; Namer, I.J.; Kremer, S. Evolution of neuroimaging findings in severe COVID-19 patients with initial neurological impairment: An observational study. *Viruses* **2022**, *14*(5), 949. doi: 10.3390/v14050949.
1-center, Universidade Federal de Minas Gerais, Belo Horizonte-MG, Brazil	de Paula, J.J.; Paiva, R.E.R.P.; Souza-Silva, N.G.; Rosa, D.V.; Duran, F.L.S.; Coimbra, R.S.; Costa, D.S.; Dutenhefner, P.R.; Oliveira, H.S.D.; Camargos, S.T.; Vasconcelos, H.M.M.; de Oliveira, Carvalho, N.; da Silva, J.B.; Silveira, M.B.; Malamut, C.; Oliveira, D.M.; Molinari, L.C, de Oliveira, D.B.; Januário, J.N.; Silva, L.C, De Marco, L.A.; Queiroz, D.M.M.; Meira, W.; Busatto, G.; Miranda, D.M.; Romano-Silva, M.A. Selective visuoconstructional impairment following mild COVID-19 with inflammatory and neuroimaging correlation findings. *Mol. Psychiatry* **2023**, *28*(2), 553-563. doi: 10.1038/s41380-022-01632-5. Epub 2022 Jun 14.
1 center, Aurora, Colorado, USA	Callen, A.L.; Tanabe, J.; Thaker, A.A.; Pollard, R.; Sauer, B.; Jones, W.; Pattee, J.; Steach, B.; Timpone, V.M. Evaluation of cerebrovascular reactivity and vessel wall imaging in patients with prior COVID-19: A prospective case-control MRI study. *AJR Am. J. Roentgenol.* **2023**, *220*(2), 257-264. doi: 10.2214/AJR.22.28267. Epub 2022 Aug 24.
1-center, Universidad Complutense, Madrid, Spain	Díez-Cirarda, M.; Yus, M.; Gómez-Ruiz, N.; Polidura, C.; Gil-Martínez, L.; Delgado-Alonso, C.; Jorquera, M.; Gómez-Pinedo, U.; Matias-Guiu, J.; Arrazola, J.; Matias-Guiu, J.A. Multimodal neuroimaging in post-COVID syndrome and correlation with cognition. *Brain* **2022**:awac384. doi: 10.1093/brain/awac384. Online ahead of print 2022 Oct 26.
1-center, Nancy, France	Goehringer, F.; Bruyere, A.; Doyen, M.; Bevilacqua, S.; Charmillon, A.; Heyer, S.; Verger, A. Brain 18F-FDG PET imaging in outpatients with post-COVID-19 conditions: findings and associations with clinical characteristics. *Eur. J. Nucl. Med. Mol. Imaging* **2023**, *50*(4), 1084-1089. doi: 10.1007/s00259-022-06013-2. Epub 2022 Nov 2.
1-center, San Raffaele, Milan, Italy	Paolini, M.; Palladini, M.; Mazza, M.G.; Colombo, F.; Vai, B.; Rovere-Querini, P.; Falini, A.; Poletti, S.; Benedetti, F. Brain correlates of subjective cognitive complaints in COVID-19 survivors: A multimodal magnetic resonance imaging study. *Eur. Neuropsychopharmacol*. **2023**, *68*, 1-10. doi: 10.1016/j.euroneuro.2022.12.002. Epub 2022 Dec 12.
1-center, Kirsehir, Turkey	Kamasak, B.; Ulcay, T.; Nisari, M.; Gorgulu, O.; Akca, V.; Alpaslan, M.; Yetis, A.; Hizmali, L.; Karahocagil, M.K.; Aycan, K. Effects of COVID-19 on brain and cerebellum: a voxel based morphometrical analysis. *Bratisl. Lek. Listy*. **2023**. doi: 10.4149/BLL_2023_068. Online ahead of print 2023 Feb 28.
6-center, Amsterdam, Maastricht, Utrecht, Zuyderland, Onze Lieve Vrouwe Gasthuis, and Diakonessenhuis Utrecht	Klinkhammer, S.; Horn, J.; Duits, A.A.; Visser-Meily, J.M.A.; Verwijk, E.; Slooter, A.J.C.; Postma, A.A.; van Heugten, C.M; NeNeSCo study group. Neurological and (neuro)psychological sequelae in intensive care and general ward COVID-19 survivors. *Eur. J. Neurol.* **2023**. doi: 10.1111/ene.15812. Online ahead of print 2023 Apr 3.
UK BioBank from all over UK	Douaud, G.; Lee, S.; Alfaro-Almagro, F.; Arthofer, C.; Wang, C.; McCarthy, P.; Lange, F.; Andersson, J.L.R.; Griffanti, L.; Duff, E.; Jbabdi, S.; Taschler, B.; Keating, P.; Winkler, A.M.; Collins, R.; Matthews, P.M.; Allen, N.; Miller, K.L.; Nichols, T.E.; Smith, S.M. SARS-CoV-2 is associated with changes in brain structure in UK Biobank. *Nature* 2022, *604*(7907), 697-707. doi: 10.1038/s41586-022-04569-5. Epub 2022 Mar 7.
2-center, Van and Kırıkkale, Turkey	Burulday, V.; Bayar, Muluk, N.; Akgül, MH.; Sayar, MS. Diffusion-weighted imaging measurements of central smell regions in COVID-19 patients: insular gyrus, corpus amygdala, and thalamus. *Eur Rev Med Pharmacol Sci.* 2023;*27*(7):3201-3207. doi: 10.26355/eurrev_202304_31954.
1-center, , Johns Hopkins Hospital, Baltimore, USA	Debs, P.; Khalili, N.; Solnes, L.; Al-Zaghal, A.; Sair, HI.; Yedavalli, V.; Luna, LP. Post-COVID-19 brain [18F] FDG-PET findings: A retrospective single-center study in the United States. *AJNR Am J Neuroradiol.* 2023. doi: 10.3174/ajnr.A7863. Epub ahead of print 2023 Apr 27.
